# How data analysis affects power, reproducibility and biological insight of RNA-seq studies in complex datasets

**DOI:** 10.1093/nar/gkv736

**Published:** 2015-07-21

**Authors:** Lucia Peixoto, Davide Risso, Shane G. Poplawski, Mathieu E. Wimmer, Terence P. Speed, Marcelo A. Wood, Ted Abel

**Affiliations:** 1 Department of Biology, University of Pennsylvania, Smilow Center for Translational Research, Room 10-170, Building 421, 3400 Civic Center Boulevard, Philadelphia, PA 19104-6168, USA; 2 Division of Biostatistics, School of Public Health, University of California, Berkeley, 344 Li Ka Shing Center, #3370, Berkeley, CA 94720-3370, USA; 3 Department of Statistics, University of California, Berkeley, Department of Mathematics and Statistics, The University of Melbourne, Bioinformatics Division, The Walter and Eliza Hall Institute of Medical Research, Australia; 4 University of California, Irvine, Department of Neurobiology and Behavior, USA

## Abstract

The sequencing of the full transcriptome (RNA-seq) has become the preferred choice for the measurement of genome-wide gene expression. Despite its widespread use, challenges remain in RNA-seq data analysis. One often-overlooked aspect is normalization. Despite the fact that a variety of factors or ‘batch effects’ can contribute unwanted variation to the data, commonly used RNA-seq normalization methods only correct for sequencing depth. The study of gene expression is particularly problematic when it is influenced simultaneously by a variety of biological factors in addition to the one of interest. Using examples from experimental neuroscience, we show that batch effects can dominate the signal of interest; and that the choice of normalization method affects the power and reproducibility of the results. While commonly used global normalization methods are not able to adequately normalize the data, more recently developed RNA-seq normalization can. We focus on one particular method, RUVSeq and show that it is able to increase power and biological insight of the results. Finally, we provide a tutorial outlining the implementation of RUVSeq normalization that is applicable to a broad range of studies as well as meta-analysis of publicly available data.

## INTRODUCTION

The sequencing of the full transcriptome (RNA-seq) has become the preferred choice for the measurement of genome-wide gene expression. Despite its widespread use, several challenges remain in RNA-seq data analysis. One often overlooked aspect is normalization, which is the transformation of values that allows comparisons between samples in a way that eliminates the effects of sources of variability that are not of interest. We refer to those effects as ‘unwanted variation’. A variety of technical and biological factors, collectively known as ‘batch effects’, contribute unwanted variation to genome-wide gene expression data. These factors include differences in amount of RNA, library preparation, equipment, operators, and procedures for sample extraction, preservation, or storage. Proper normalization, or removal of these factors, has been shown to critically impact the analysis of high-throughput data (1–3). In spite of this, commonly used methods for RNA-seq normalization, such as upper quartile scaling (UQ)(2), trimmed mean of M values (TMM)(4) and FPKM (5), account only for global differences in sequencing depth between libraries (6).

The use of RNA-seq to study gene expression is particularly problematic when it is influenced simultaneously by a variety of biological factors in addition to the one of interest, such as: genetic background, time of day, differences in responsiveness between individuals and cell-type heterogeneity. Proper experimental design is highly beneficial but may not be enough when factors contributing unwanted variation are unknown. In this study we look at recently published studies applying RNA-seq technology in the context of experimental neuroscience. This type of study represents a good middle ground to study the effect of unwanted variables in RNA-seq experiments. Unlike other experimental systems, many unwanted variables cannot be controlled when studying gene expression in the brain *in vivo*, because their influence is usually unknown. Nonetheless experimental neuroscience studies will be less influenced by unwanted variables than non-experimental systems, such as studies in human samples.

Our results show that batch effects are prevalent. We demonstrate that commonly used global normalization methods are inadequate. Normalization methods that can account for batch effects have been recently developed. Focusing on one particular method, RUV (6), we show that it can remove unwanted variation and lead to a more accurate and reproducible picture of gene expression changes. Finally, we provide tutorials on RUV normalization that allows the reader to reproduce the figures of this article and are applicable to a broad range of studies.

## MATERIALS AND METHODS

Publicly available data were downloaded from GEO (see Supplementary Table S1 for details). Data generated in this article are available through GEO (GSE63412).

### Subjects

C57BL/6J adult male mice (2 months of age) were obtained from Jackson Laboratories and housed individually for a week on a 12 h/12 h light/dark schedule with lights on at 7 a.m. (Zeitgeber time (ZT) 0). Food and water were available *ad libitum* throughout the experiment. Each animal was handled daily for 3 days prior to contextual fear conditioning (FC). Handling consisted of manipulation of the animals for 1–2 min per mouse in the same room as the experimental setting without exposure to the context. The conditioning protocol entailed a single 2-s, 1.5-mA foot shock, terminating at 2.5 min after placement of the mouse in the chamber, starting at 10 a.m. (ZT3) daily. Hippocampal dissections were performed immediately following the behavioral treatment, and alternated between FC and control animals. Tissue was collected at 30 min after FC (FC) as well as 30 min after testing for retrieval of the memory (RT). Testing was performed at 24 h after training over a 5-min interval, which is sufficient to induce reconsolidation (7,8). The average freezing was 55 ± 10%. Tissue was immersed in RNAlater (Qiagen) and immediately frozen. Animals that were handled but not trained were dissected at the same time of day (CC). The protocol was repeated over the course of 2 weeks to obtain 5 animals (2 hippocampi) per group (FC, RT, CC) each representing an independent FC experiment, so that all animals for each group were dissected at the same time of day on different days. Each sequencing library was prepared from RNA extracted from two pooled hippocampi from one mouse. All experiments were approved by the Institution of Animal Care and Use Committee of the University of Pennsylvania and were carried out in accordance with all National Institutes of Health guidelines.

### RNA sequencing, mapping and filtering

RNA extraction was performed using Qiagen RNAeasy Microarray Tissue kit. All RNA extractions were performed the same day within a week of tissue collection. Induction of positive controls after FC *Arc*, *Fos* and *Dusp1* was confirmed by qPCR. Two microgram of RNA was used for library preparation using the TruSeq RNA Sample Prep Kit (Illumina, San Diego, CA, USA) with Poly A selection according to the manufacturer's instructions. Completed libraries were size-selected (200–400 bp) on an agarose gel to remove any high basepair fragments, quantified by qPCR (KAPA Biosystems, Boston, MA, USA), and submitted to the Penn Genome Frontiers Institute (PGFI) sequencing core at UPENN for sequencing. Three libraries were multiplexed per lane (one biological replicate of each three conditions) and sequenced on an Illumina HiSeq 2000 using Type 3 chemistry, resulting in an average of 64 million 100 bp pair-end reads per sample. Data are publicly available through GEO (GSE63412). Sequencing data from RNA obtained from hippocampus of six animals sacrificed 30 minutes following object-location memory (OLM) and their corresponding controls (HC) were generated in the Wood lab at the University of California Irvine and are publicly available through GEO (GSE44229) (9). Reads were mapped to the mouse genome (mm9) using GMAP/GSNAP(10). Only unique and concordant mapped reads were subsequently used for feature quantification. Ensembl (release 65) gene counts were obtained using HTSeq 0.6.1 (11). Only genes with at least 10 reads in at least 5 samples were considered for further analysis (Supplementary Table S2).

### Statistical analysis

All analyses were performed using open source software freely available through the R/Bioconductor project (12). RLE and PCA Plots for exploratory analysis as well as upper-quantile normalization (UQ) were performed using EDASeq (v. 2.0.0) (13). RUV normalization was performed using RUVSeq (v. 1.0.0) (6) after the data was normalized by UQ using EDASeq. Differential expression analysis was performed using EdgeR (v. 3.8.2) (14). 625 negative controls for normalization were obtained as genes with an uncorrected *P*-value >0.8 in all pairwise comparisons between RT, FC and CC in microarray data available through GEO (GSE50423) (15). The R code to reproduce all the main figures and tables of the article is available as tutorials in the supplementary material and downloadable form GitHub (github.com/drisso/peixoto2015_tutorial).

### Functional annotation analysis

Enrichment of functional annotation on differentially expressed gene sets was assessed using the Database for Visualization and Integrative Discovery (DAVID) (16). Functional annotation was limited to KEGG pathways. Enrichment for each term was defined relative to all mouse genes with at least 10 reads in at least five samples, and was defined as an EASE score <0.1 with at least three genes per term per dataset.

## RESULTS

### Global scaling normalization methods do not correct for unwanted variation in the data

To assess whether unwanted variation is a problem within RNA-seq studies in experimental neuroscience, we re-analyzed studies of the mouse hippocampus *in vivo*, available in GEO (http://www.ncbi.nlm.nih.gov/geo/). We required that the studies met minimal quality criteria: have an associated PMID, were sequenced with Illumina HiSeq technology at a depth of at least 10 million reads per sample, include at least six samples with a minimum of 2 biological replicates per condition and include gene-level read summaries. A summary of the studies can be found in Supplementary Table S1. We also included our own previously published study following object location memory (OLM, GSE44229). All studies used established methodology for RNA-seq data analysis and normalization methods that only correct for sequencing depth, which is standard practice. The studies cover a variety of experimental manipulations used in neuroscience research, such as comparison between knock-out versus wild-type animals, injection of shRNAs to inhibit the expression of a gene, age, induction of neurodegeneration, and learning and memory paradigms.

**Figure 1.**
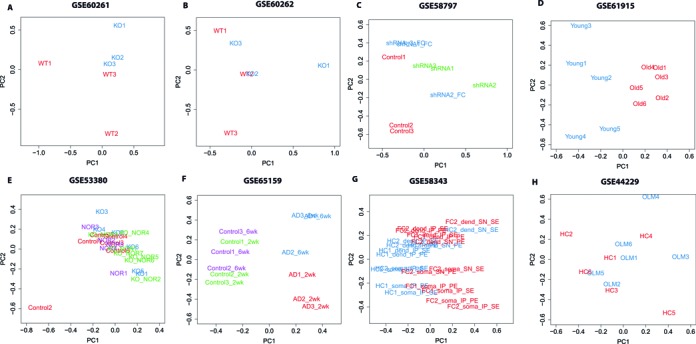
Unwanted variation dominates the signal in RNA-seq studies in experimental neuroscience. PCA plots of gene counts normalized using either upper-quantile (UQ) or FPKM from publicly available datasets from the mouse hippocampus. (**A**) GSE0261, mRNA-Seq of wild-type (in red) versus knock-out mice (in blue). A severe batch effect is observed in the WT samples (40). (**B**) GSE0262, small RNA-Seq of wild-type (in red) versus knock-out mice (in blue). A severe batch effect is observed in the WT and KO samples (40). (**C**) GSE58797, mRNA-seq of mice injected with shRNA to knock down expression of a gene (green), scrambled shRNA (red, controls) and injected with shRNA to knock down expression of a gene and submitted to contextual fear conditioning (FC, blue). A batch effect can be observed in the controls, and there's no separation between FC and naïve injected animals (41). (**D**) GSE61915, mRNA-seq of young (3 weeks, blue) versus old (24 weeks, red) animals. Proper grouping of treatment samples is observed (42). (**E**) GSE53380, mRNA-seq of wild-type (control, in red), KO animals (in blue), WT animals following novel-object recognition (NOR, purple) and KO animals following NOR (green). One control sample is an outlier, no separation is observed among all other samples (43). (**F**) GSE65159, mRNA-seq of animals 2 weeks (2wk,red) and 6 weeks (6wk, blue) following the induction of p25 expression (mouse model of Alzheimer's disease, AD) an their respective controls (green and purple). As expected no difference is observed in time without induction of neurodegeneration, proper separation of samples by treatment is observed in the AD mouse model (44). (**G**) GSE58343, mRNA-seq of home cage (HC, blue) and fear-conditioned animals (FC, red). Includes pair-end (PE) and single-end (SE) technical replicates, RNA obtained from neuronal dendrites (dend) vs. soma, and RNA following ribosome imuno-precipitation (IP) versus supernatant of the same sample (SN). There is no separation between HC and FC samples, or IP and SN samples (45). (**H**) GSE44229. mRNA-seq of home-cage (HC, red) versus animals obtained following object location memory (OLM, blue). There's no separation between HC and OLM samples (24).

The general assumption of most studies is that the experimental manipulation of interest is the main source of variation in the data. The main problem when studying the brain is that a lot of variables cannot be controlled, so this assumption may not hold true. One way to visualize the sources of variation in the data is to use principal component analysis (PCA). The use of PCA for data exploration and quality control is an established practice in genome-wide expression studies. PCA is a statistical procedure that looks for a small set of linear combinations of the original variables to summarize the data losing as little information as possible (17). These linear combinations are called principal components (PCs): the first PC is the weighted average of the gene expression measures that gives the highest variance across all samples. Each succeeding component in turn has the highest variance possible under the constraint that it is uncorrelated with the preceding components. The clustering of samples by treatment in the space of the first two principal components is a good indicator of the quality of the data. Since the samples differ only in the treatment of interest, provided that appropriate normalization has been carried out, we expect this to be the main driver of the clustering. If the samples fail to cluster by treatment, the main source of variation is not the treatment of interest and this could lead to false positives or false negatives among the differentially expressed genes. The majority of the publicly available studies that we obtained from GEO do not show proper grouping according to treatment in a PCA plot following standard normalization procedures (Figure 1). With the exception of a big difference in age (Figure 1D) or a potent induction of neurodegeneration (Figure 1F), the effect size of the experimental manipulations was not enough to overcome the unwanted variability.

To further investigate how normalization of RNA-seq affects the detection of differential expression in the brain, we focused on long-term memory formation, since learning and memory paradigms are particularly problematic (Figure 1). One of the fundamental questions in neuroscience is how memories are stored and retrieved in the brain. It has long been known that long-term memory formation requires transcription (18,19). There are published findings of genome-wide studies of gene expression following memory acquisition using microarrays (20–22), however only a small number of genes are observed to be consistently regulated across studies. We examined genome-wide changes of gene expression for two commonly used paradigms for hippocampus-dependent long-term memory formation: object location memory (OLM) (9) and contextual fear conditioning (FC)(23). Sequencing data from RNA obtained from hippocampus 30 minutes following object-location memory (OLM) and their corresponding controls (HC) were generated in the Wood lab (24) and are publicly available (GSE44229) (Figure 1H). Sequencing data from RNA obtained from hippocampus 30 minutes following contextual fear conditioning (FC), 30 minutes following retrieval of memory (RT) and their corresponding controls (CC) were obtained in the Abel lab and are available through this article (GSE63412) (see ‘Materials and Methods’ for details on data analysis).

**Figure 2.**
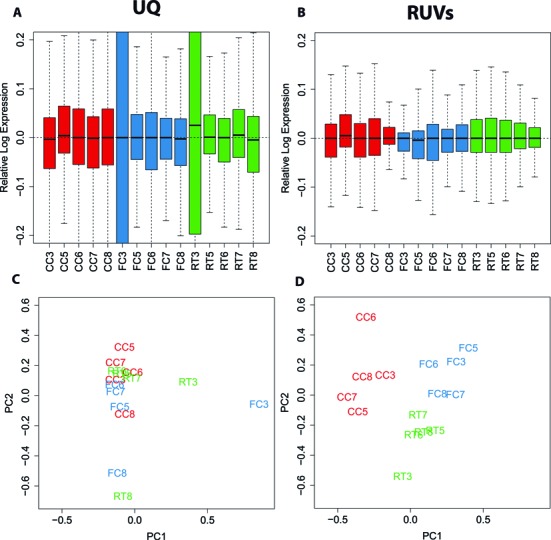
RUV normalization corrects for unwanted variation in FC data. In red control samples matched for time of day (CC), in blue samples obtained 30 min after memory acquisition (FC), in green samples obtained 30 min after memory retrieval (RT). (**A**) Relative log expression (RLE) plot of all samples following traditional upper-quartile normalization (UQ). (**B**) RLE plots following normalization with RUV using negative controls and samples (RUVs). (**C**) Scatterplot of first two principal components (log-scaled, centered counts) following UQ normalization. The first two PCs explained 66% and 6% of the variance, respectively. (**D**) Scatterplot of first two principal components following RUVs normalization. The first two PCs explained 19.9% and 13.1% of the variance, respectively. Samples do not cluster according to treatment following UQ normalization but do so after applying RUVs. UQ normalization and RLE and PCA plots were performed using the R/Bioconductor package EDASeq (v. 2.0.0). RUVs normalization was performed using the R/Bioconductor package RUVSeq (v. 1.0.0).

Figure 2A shows box plots of relative log expression (RLE) (25) among FC, RT and CC replicates. RLE plots should be centered on 0 and as similar as possible to each other when samples are properly normalized. A commonly used global normalization method such as Upper-Quartile (UQ) centers the means on 0, but is not able to normalize the higher levels of variation present in replicates FC3 and RT3 (Figure 2A). Similar results were obtained using TMM, while FPKM is the worst performing normalization method as previously shown (2,3) (Supplementary Figure S1A). The presence of unwanted variation that is not removed using global normalization methods is also evident by the samples failure to cluster by treatment following principal component analysis (PCA). The PCA plots obtained using UQ, TMM and FPKM normalization do not constitute an improvement over raw counts (Figure 2C and Supplementary Figure S1B). Lack of clustering of biological replicates in the PCA plots indicates that unwanted variation dominates the signal. This in turn will lead to false negatives (lack of power) or false positives (inaccurate results) and limit the reproducibility of the differential expression analysis. These results parallel what we observed in publicly available datasets (Figure 1). RNA-seq normalization methods that are able to correct for factors other than sequencing depth are available and include: RUV, a normalization method we have recently published that uses factor analysis to remove systematic artifacts (6,26), PEER (27,28) and SVA (29,30). Figure 2B and D show the results of applying RUV to our FC dataset. RUV is based on the use of negative control genes or samples, that is, genes or samples that are not expected to be influenced by the biological covariates of interest. We obtained 625 negative control genes using microarray data that contained CC, FC and RT samples (GSE50423) (15). We defined a negative control gene as one whose *P*-value of differential expression between CC, FC and RT was >0.8. A full list of negative control genes can be found in Supplementary Table S3. Negative control samples were constructed by computing differences of biological replicates within the same treatment condition as detailed in (6). RUV normalization using negative control genes and samples (RUVs), modeling *k* = 5 factors of unwanted variation, restores the expected distribution to the RLE and PCA plots (Figure 2B and D).

The ability of RUVs to restore the expected distribution to RLE and PCA plots is also seen in the OLM data (Supplementary Figures S2 and S3). We also evaluated the ability of SVA and PEER to remove unwanted variation from the FC data (Supplementary Figure S4). Both methods constitute an improvement over global normalization methods, but only SVA is effective. PEER needs the specification of the number of factors of unwanted variation; both *k* = 5 and *k* = 1 lead to unsatisfactory results. When SVA is run on default mode, it infers only one factor of unwanted variation (*k* = 1) and is not sufficient to normalize the samples (both in supervised and unsupervised mode). When manually including 5 surrogate variables in the SVA model, the results are similar to those observed for RUV. Thus, the choice of *k* is a key factor in achieving proper normalization, whether RUV or SVA are used.

### RUVs normalization is robust to the choice of negative controls

**Figure 3.**
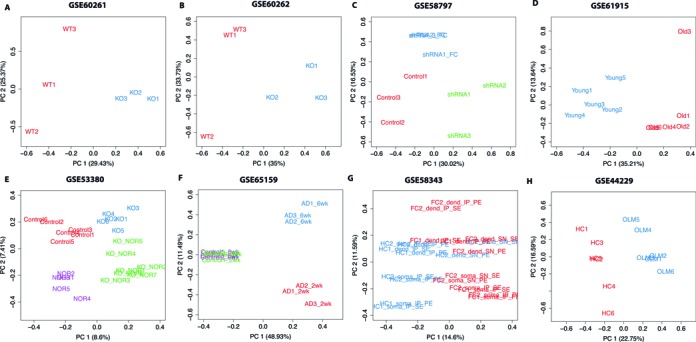
RUV normalization corrects for unwanted variation in GEO datasets. PCA plots of RUVs normalized gene counts (using all genes as negative controls) from publicly available datasets from the mouse hippocampus. (**A**) GSE0261, mRNA-Seq of wild-type (in red) versus knock-out mice (in blue). Batch effect no longer evident (40). (**B**) GSE0262, small RNA-Seq of wild-type (in red) versus knock-out mice (in blue). Batch effect no longer evident (40). (**C**) GSE58797, mRNA-seq of mice injected with shRNA to knock down expression of a gene (green), scrambled shRNA (red, controls) and injected with shRNA to knock down expression of a gene and submitted to contextual fear conditioning (FC, blue). Batch effect no longer evident (41). (**D**) GSE61915, mRNA-seq of young (3 weeks, blue) versus old (24 weeks, red) animals. Proper grouping of treatment samples is mantained (42). (**E**) GSE53380, mRNA-seq of wild-type (control, in red), KO animals (in blue), WT animals following novel-object recognition (NOR, purple) and KO animals following NOR (green). Proper grouping of experimental conditions is improved (43). (**F**) GSE65159, mRNA-seq of animals 2 weeks (2wk,red) and 6 weeks (6wk, blue) following the induction of p25 expression (mouse model of Alzheimer's disease, AD) an their respective controls (green and purple). As expected no difference is observed in time without induction of neurodegeneration, proper separation of samples by treatment is improved (44). (**G**) GSE58343, mRNA-seq of home cage (HC, blue) and fear-conditioned animals (FC, red). Includes pair-end (PE) and single-end (SE) technical replicates, RNA obtained from neuronal dendrites (dend) versus soma, and RNA following ribosome imuno-precipitation (IP) versus supernatant of the same sample (SN). Separation separation between HC and FC samples, as well as IP and SN samples is improved (45). (**H**) GSE44229. mRNA-seq of home-cage (HC, red) versus animals obtained following object location memory (OLM, blue). Batch effect no longer present (24).

An important issue regarding the applicability of RUV normalization to a wide variety of datasets is how well the method performs when negative controls are not available. Results of RUV normalization of FC data are similar when using only negative control genes (RUVg, Supplementary Figure S5) or when using negative control samples and considering all genes as negative controls (RUVall, Supplementary Figure S6). We have previously demonstrated that in fact RUV (and RUVs in particular) is quite robust to the choice of negative control genes (6). Without knowing what genes will be appropriate negative controls for the publicly available datasets in Figure 1, we can implement RUVs assuming all genes as negative controls (Figure 3). The degree to which this strategy is effective depends on the effect size of the treatment, or in other words in the proportion of the total genes that is in fact differentially expressed, as well as the number of biological replicates. Figure 3 shows that normalization is greatly improved using this strategy. If using all genes as negative controls, proper randomization of samples is essential for RUVs to be effective. RUVs will only be able to remove the unwanted variation observed within replicate samples, and it will not be effective when there is perfect confounding between the biological effect of interest and batch effects (e.g. if all the knock-out samples are prepared in a different day or by a different technician than the wild-type samples). The use of negative control genes that have been obtained empirically from an independent dataset in conjunction with the biological replicates is preferable and gives better results than using only the replicate samples. Negative control genes can be obtained from publicly available datasets of similar experimental conditions, by using either *P*-values (as we did in this article) or entropy (31).

### Removal of unwanted variation leads to quantitative and qualitative improvements on differential expression analysis

**Figure 4.**
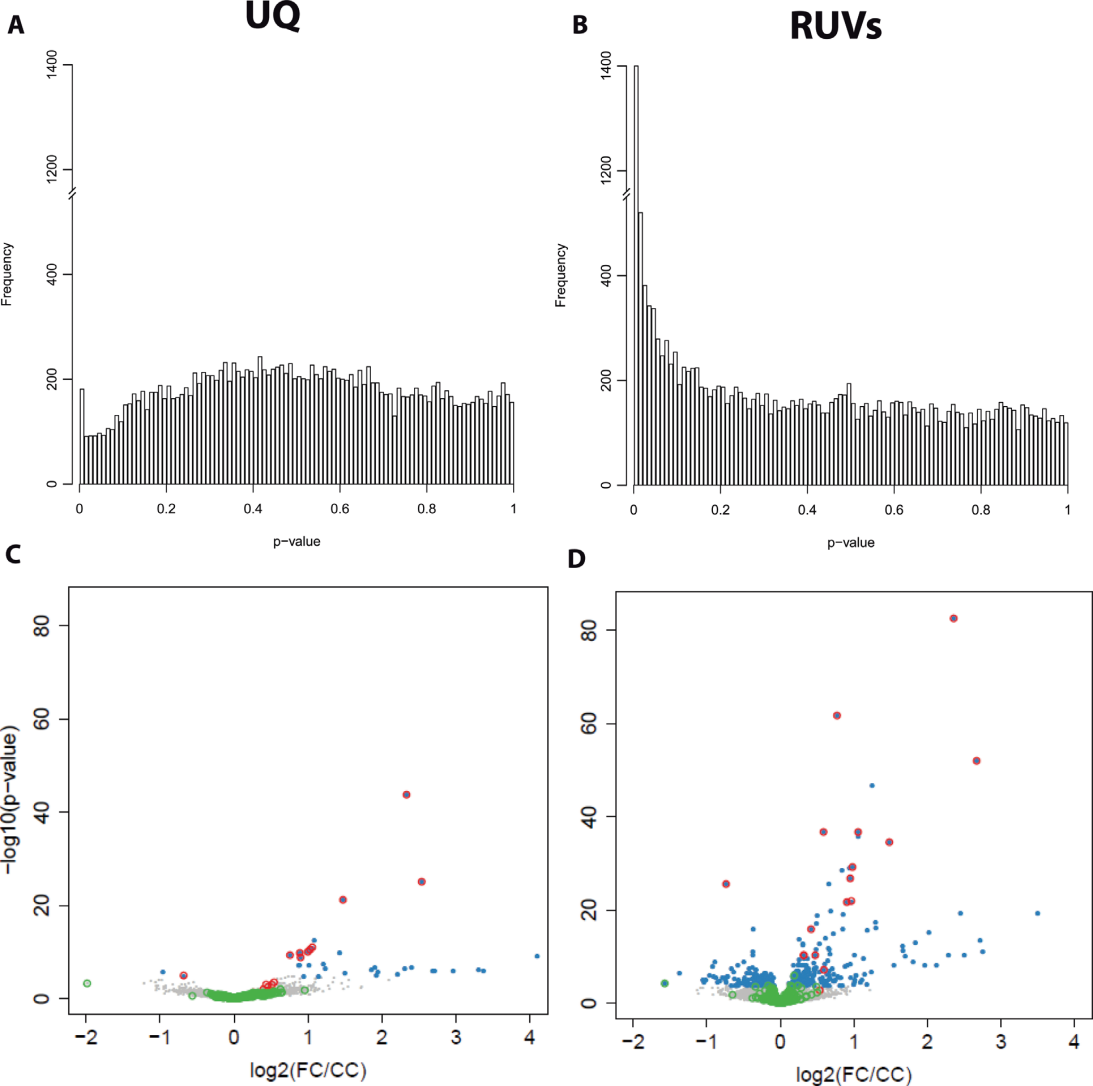
Normalization impacts differential expression after contextual fear conditioning. (**A**) Distribution of unadjusted edgeR p-values for tests of differential expression between FC and CC samples following UQ normalization. (**B**) Distribution of unadjusted edgeR *P*-values for tests of differential expression between FC and CC samples following UQ normalization. The distribution of *P*-values following UQ normalization is far from the expected uniform. RUV returns uniformity to the p-value distribution and increases discovery of differentially expressed genes (genes that have a low *P*-value). (**C**) Volcano plot of differential expression (−log_10_*P*-value versus log fold change) of UQ normalized samples. (**D**) Volcano plot of differential expression of RUVs normalized samples. Genes with and FDR <0.01 are highlighted in blue. Positive controls are circled in red, negative controls are circled in green (Table S2). RUV increases the detection of known differentially expressed genes from 60% to 94%. Differential expression analysis was performed using R/Bioconductor package edgeR (v. 3.8.5).

To evaluate the impact of normalization methods on differential expression (DE), we analyzed UQ and RUVs normalized data using edgeR (14,32). Figure 4A and B shows the unadjusted *P*-value histograms of DE between CC and FC samples following UQ and RUVs normalization. A satisfactory *P*-value histogram should contain a sharp peak at zero representing genes with strong DE and a ‘floor’ of values that is approximately uniform in the interval [0, 1], corresponding to genes that are not DE. Lack of uniformity in the p-value distribution, such as the one observed after UQ, suggests the presence of confounding variables not accounted for in the model. RUVs restores uniformity to the p-value distribution and increases the number of genes identified as DE (the height of the peak at zero). Figure 4C and D depicts volcano plots of p-value versus expression fold-change between CC and FC samples following UQ and RUV normalization. UQ leads to the discovery of 34 DE genes (32 up, 2 down), while RUV increases the detection power, detecting 403 DE genes (237 up, 166 down).

**Figure 5.**
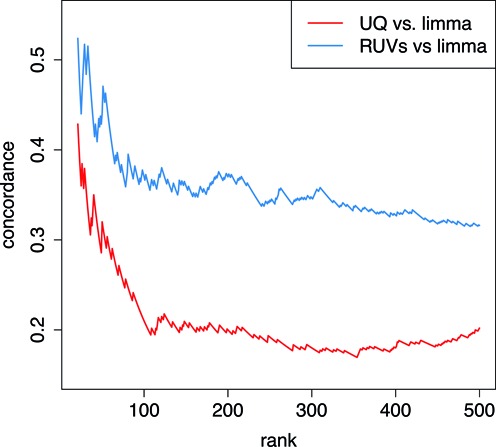
RUV increases concordance of RNA-seq and microarray differential expression following fear conditioning. Y-axis: number of genes in agreement between microarray and RNA-seq data at any given rank. X-axis: differential expression rank (low to high *P*-value). In red: differentially expressed genes obtained using edgeR for UQ normalized RNA-seq data relative to those detected by microarrays using limma. In blue: differentially expressed genes obtained using edgeR for RUVs normalized RNA-seq data relative to those detected by microarrays using limma. The agreement between technologies on the top 100 differentially expressed genes doubles with RUVs normalization.

To evaluate the impact of normalization on the quality of DE genes, we collected a set of positive control genes whose expression changes have been previously validated (Supplementary Table S3). Figure 4D shows that RUV increases detection of positive controls on the FC dataset. While only 60% of the positive controls are detected as differentially expressed after UQ normalization, 94% are detected as differentially expressed following RUVs. This is despite the fact that the controls are biased towards high fold-changes, which may explain why the estimated fold-change of the positive controls is not greatly affected by the choice of normalization method. Similar results were obtained for RT versus CC samples (Supplementary Figure S7) and OLM versus HC samples (Supplementary Figure S8). A list of genes DE at FDR <0.01 for each pairwise comparison is available in Supplementary Table S4. To further assess the quality of the DE results we evaluated which KEGG pathways were significantly enriched in the DE genes relative to all genes detected by RNA-seq in our samples (Supplementary Figure S9). Enrichment of KEGG pathways was assessed using DAVID (16) and defined as an EASE score <0.1 with at least three genes per term per dataset. Only the MAPK pathway was enriched in the DE gene set detected following FC after UQ normalization. The RUVs normalized dataset showed enrichment of MAPK, T-cell and Toll-like receptor (NF-κB/cytokines, Jak/STAT), GnRH (cAMP/PKA,CREB, PKC) and Insulin signaling pathways in the upregulated genes following FC. This is consistent with the previously established role of these pathways in learning and memory (33–37). We also hypothesized that the increase of detection of true biological signal would lead to a higher agreement in DE across technologies. Figure 5 shows the concordance of the DE ranks following FC obtained using edgeR for UQ or RUVs normalized RNA-seq data relative to differential expression detected using limma (38) on microarray data without removal of unwanted variation (GSE50423). Removing unwanted variation in the RNA-seq dataset by RUVs improves consistency between platforms, doubling the concordance of the top 500 DE genes despite the fact that no unwanted variation was removed from the microarray data. These findings provide further evidence that RUVs is increasing detection of true biological signal.

### Removal of unwanted variation is necessary for cross-site data integration

**Figure 6.**
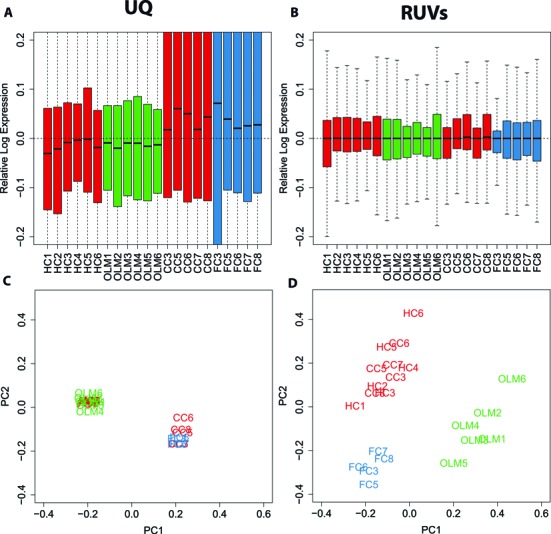
RUV allows removal of laboratory specific effects for combined analysis of gene expression changes following FC and OLM. In red control samples matched for time of day (CC), in blue samples obtained 30 min after memory acquisition (FC), in green samples obtained after object location memory (OLM). (**A**) Relative log expression (RLE) plot of all samples following upper-quartile normalization (UQ). (**B**) RLE plots following normalization with RUV using negative controls and samples (RUVs). (**C**) Scatterplot of first two principal components (log-scaled, centered counts) following UQ normalization. The first two PCs explained 73.4% and 9.6% of the variance, respectively. (**D**) Scatterplot of first two principal components following RUVs normalization. The first two PCs explained 15.5% and 9.4% of the variance, respectively. Samples cluster according to laboratory following UQ normalization but cluster according to treatment after applying RUVs.

**Figure 7.**
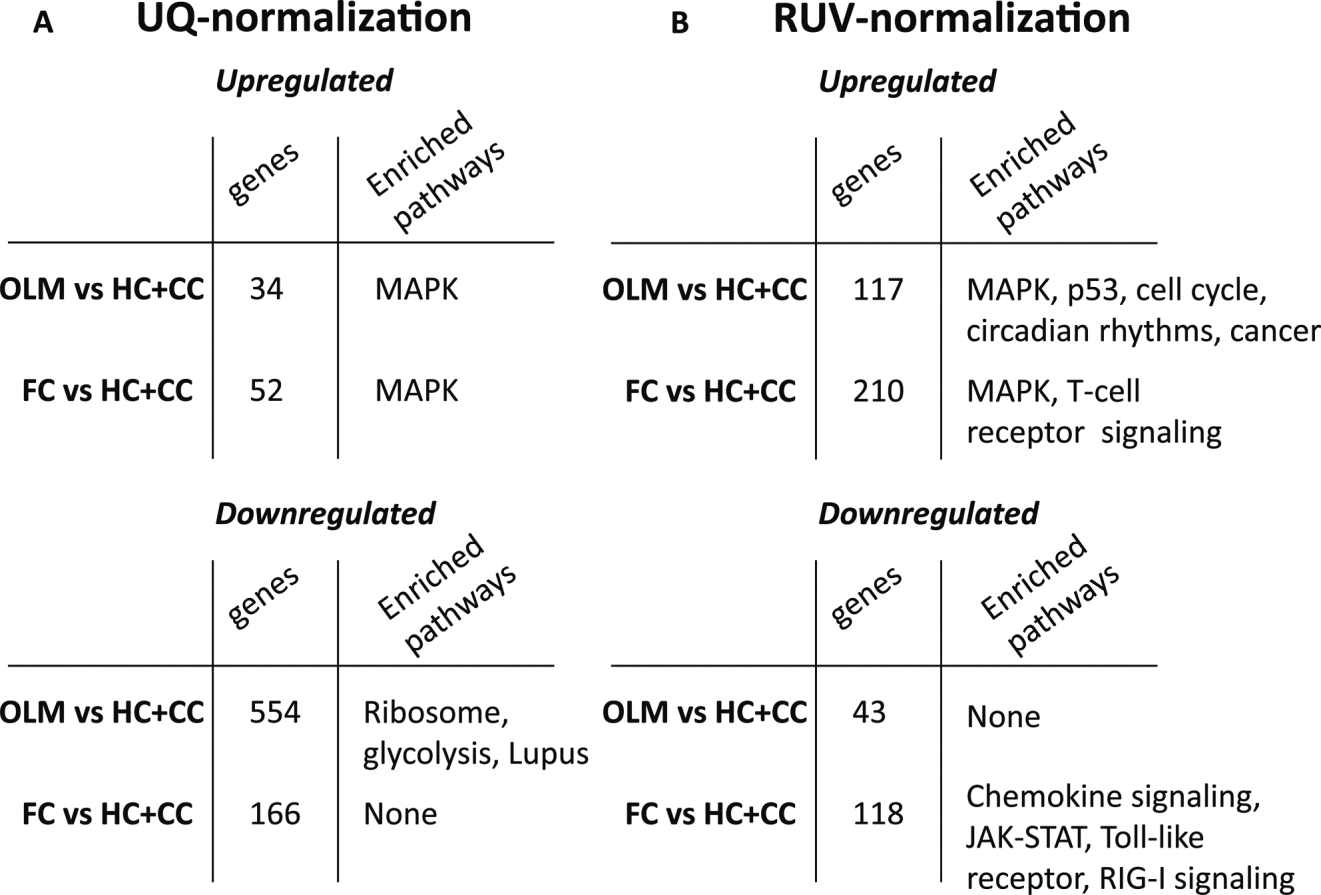
Quantitative and qualitative effects of the choice of normalization method in combined analysis of gene expression changes following FC and OLM. (**A**) Number of genes and enriched KEGG pathways for OLM and FC relative to combined controls following UQ normalization. UQ normalization leads to inferring housekeeping genes as differentially expressed. (**B**) Number of genes and enriched KEGG pathways for OLM and FC relative to combined controls following RUVs normalization. The apparent regulation of housekeeping genes has been removed.

The ability to integrate datasets, either within multi-site sequencing projects or to perform meta-analysis of publicly available data holds great promise as a way to increase biological insight while maximizing investment of resources. The challenge of cross-site data integration is that differences in protocols, reagents and operators will produce site-specific batch effects that can obscure the biological signal. To evaluate the ability of RNA-seq normalization methods to deal with multi-sites batch effects, we combined the FC and CC samples obtained in the Abel laboratory with the HC and OLM samples obtained in the Wood laboratory. Figure 6A and C shows the RLE and PCA plots following UQ, in which a site-specific batch effect is evident. We applied RUVs using the set of negative controls in Supplementary Table S3 and combining HC and CC samples as controls, since they both represent the same biological condition. The distribution of the samples in the RLE and PCA plots is noticeably improved following RUVs (Figure 6B and D). Next, we used edgeR to evaluate differences in gene expression of OLM or FC samples versus the combined CC+HC controls for UQ and RUVs normalized datasets. Figure 7 shows the number of genes regulated by OLM and FC (FDR < 0.01) and the corresponding enriched KEGG pathways. The lists of DE genes for the combined analysis (FDR < 0.01) are available in Supplementary Table S5. UQ causes a disproportionate increase in downregulated genes for both comparisons (Figure 7A). This is particularly evident for the OLM versus HC + CC comparison for which we observed an enrichment in ‘Ribosome’ and ‘Glycolysis’ KEGG pathways, often thought of as ‘housekeeping’ functions and likely false positives. RUVs removes this effect (Figure 7B). The resulting number of DE genes at FDR <0.01 following FC is slightly less than when the datasets are analyzed separately. Combining the datasets considerably increases the number of genes and pathways detected following OLM (Figure 7B). More importantly, it allows us to ask what are the genes and pathways that are regulated by both FC and OLM (FC + OLM versus HC + CC) as well as exclusively by FC or OLM (FC versus OLM). The number of genes DE following both tasks is 308 at FDR <0.01 while no genes are detected as DE between them (Supplementary Table S5). Combining the datasets identifies as upregulated the MAPK, Jak/STAT and Insulin signaling pathways, all of which are known to be involved in memory and synaptic plasticity (35,37,39). When we normalize FC or OLM datasets independently using RUVs only 46 genes overlap between the two lists (Supplementary Table S4), suggesting that independent analyses are not as powerful and illustrating the benefit of integrating data across sites. Analyzing the combined datasets using UQ results in 7000 genes identified as DE between FC and OLM, including the majority of ‘housekeeping’ genes (Supplementary Table S5).

### A primer to increase power and reproducibility of RNA-seq studies in neuroscience

**Figure 8.**
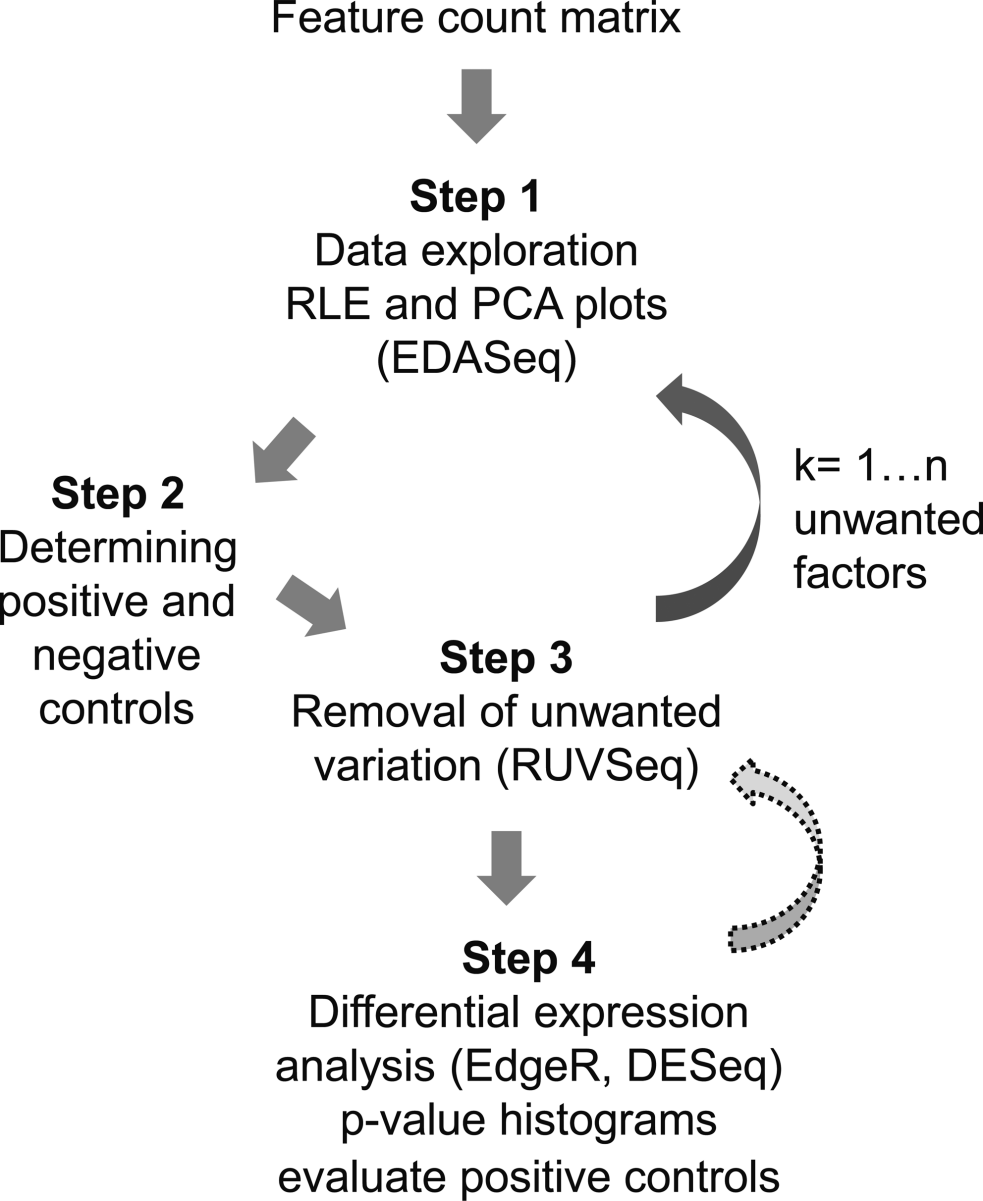
Step-by-step outline of the application of RUV to normalization of RNA-seq data.

Figure 8 presents a step-by-step guide to implement normalization of RNA-seq using RUVSeq (6), to be used by both authors and reviewers to guarantee high-quality RNA-seq data-analysis. We recommend that authors follow established guidelines for both alignment and feature counting (see (32)) as well as proper replication and randomization of experiments. **Step 1** is to explore the distribution of variation in the data both before normalization and following normalization for sequencing depth only (UQ, TMM), which can be done by constructing the RLE and PCA plots previously shown, using a publicly available package such as EDASeq (13). If the RLE plots are not evenly distributed and centered on 0 and the PCA plots do not display proper replicate sample clustering (Figure 2A and C), additional normalization is needed. **Step 2** is the collection of proper controls. Negative control genes can be extracted from publicly available data, as illustrated in our example. In practice, when only a small proportion of the genes in the genome is expected to be differentially expressed using all the genes as negative controls or using ‘housekeeping’ genes as negative controls are viable alternatives. Identifying a small subset of positive control genes or pathways is recommended as it provides a way to judge the results of the DE analysis. **Step 3** is the removal of unwanted variation through factor analysis. Iteratively account for *k* = 1, …, *n* factors of unwanted variation using RUV (6) or similar methods (such as SVA), checking RLE and PCA plots each time until proper distributions are restored (Figure 2B and D). **Step 4**. Perform DE analysis using a method that allows the addition of one or more terms that model the unwanted variation, such as edgeR or DESeq2 (32). Check *P*-value histograms and distribution of negative and positive controls in the sample (Figure 4) to evaluate performance. Return to **Step 3** if performance is not satisfactory. PCA plots and *P*-value histograms should be made available so that readers are able to judge the quality of the data analysis. Supplementary file 1 contains the tutorial that allows anyone to implement the suggested outline on the FC and OLM data. Supplementary file 2 contains a tutorial that allows readers to implement RUV on the publicly available datasets analyzed in this article. Collectively they allow for reproduction of all figures in this article. The source code and data necessary to run the tutorial can be downloaded through GitHub (github.com/drisso/peixoto2015_tutorial)

## DISCUSSION

We have illustrated the prevalence of batch effects in RNA-seq studies using examples from experimental neuroscience and shown that traditionally used methods for RNA-seq data normalization are not able to remove them. This problem likely extends to a variety of datasets for which sources of variation are hard to control. These limitations can lead to the identification of a small number of confident changes (a large proportion of false negatives) and/or to uncovering statistically significant changes that are not due to the treatment, which will not be reproducible (false positives). Here, we present a novel application of a recent method for RNA-seq normalization, RUV and show that it is better able to correct for unwanted sources of variation when proper controls are provided. We show that within laboratories, RUV considerably increases the number of genes discovered as differentially expressed. We demonstrate that this increase is indeed an improvement in the detection of true biological signal by showing that it increases the discovery of positive controls, known pathways involved in learning and memory and cross-platform concordance. When integrating data across different laboratories, commonly used normalization methods lead to artifacts that cause housekeeping genes to be inferred as differentially expressed. In contrast, RUV normalization is able to properly correct the biases introduced by integrating data from different sites, allowing for direct comparisons regarding differential gene expression following two different behavioral paradigms. These analyses suggest that both contextual fear conditioning and object location memory induce the same changes in gene expression. Finally, we outline a step-by-step guide on how to detect sources of unwanted variation in the data and apply RUV to remove this variation prior to differentially expression analysis. We hope these guidelines together with all the datasets generated in this article will serve as resource, for both authors and reviewers, to ensure that results obtained using high-throughput sequencing technologies are reproducible and thus truly contribute to the advance of knowledge in science.

## ACCESSION NUMBERS

Publicly available data was downloaded from GEO (9,40–45) (see Supplementary Table S1 for details). Data generated in this article is available through GEO (GSE63412).

## Supplementary Material

SUPPLEMENTARY DATA
