# Supplementary material for: How data analysis affects power, reproducibility and biological insight of RNA-seq studies in complex datasets
Source: Nucleic Acids Res. 2015 Jul 21;43(16):7664–74. doi: 10.1093/nar/gkv736 (PMC4652761; doi:10.1093/nar/gkv736)
Supplement: SUPPLEMENTARY DATA [file supp_43_16_7664__index.html]

How data analysis affects power, reproducibility and biological insight of RNA-seq studies in complex datasets — How data analysis affects power, reproducibility and biological insight of RNA-seq studies in complex datasets — SUPPLEMENTARY DATA 

# How data analysis affects power, reproducibility and biological insight of RNA-seq studies in complex datasets

## SUPPLEMENTARY DATA

- SUPPLEMENTARY DATA
- SUPPLEMENTARY DATA
- SUPPLEMENTARY DATA
- SUPPLEMENTARY DATA
- SUPPLEMENTARY DATA
- SUPPLEMENTARY DATA
- SUPPLEMENTARY DATA
