# Supplementary material for: How data analysis affects power, reproducibility and biological insight of RNA-seq studies in complex datasets
Source: Nucleic Acids Res. 2015 Jul 21;43(16):7664–74. doi: 10.1093/nar/gkv736 (PMC4652761; doi:10.1093/nar/gkv736)
Supplement: SUPPLEMENTARY DATA [file supp_gkv736_nar-01134-survey-d-2015-File010.pdf]

Additional material.

Additional Table 1. Details of publicly available datasets used in this article.

GEO accession	PUBMED ID/ phenotype assessed	Type of sequencing	Alignment algorithm	Feature counting algorithm	Data downloaded as	Normalization method used *
GSE60261	25595182/ Wild-type vs. KO mice	Illumina HiSeq 2500, SE, 50bp	Bowtie 2 (2.0.5) and TopHat (2.0.6)	Seqmonk (0.26.0)	DESeq2 normalized gene counts	DESeq2
GSE60262	25595182/ Wild-type vs. KO mice	Illumina HiSeq 2500, SE, 50bp	BWA	Seqmonk (0.26.0)	DESeq2 normalized gene counts	DESeq2
GSE58797	25219850/ Mice injected with shRNA, scrambled shRNA (controls) and injected with shRNA submitted to contextual fear conditioning	Illumina HiSeq 2000, PE, 50 bp	TopHat (1.4.0)	Cufflinks/Cuff Diff	FPKM normalized gene counts	FPKM+UQ
GSE61915	25431548/ Young vs. Old mice	Illumina HiSeq 2000, SE, 50 bp	GRCm38.p2, STAR 2.3.0	HTSeq	DESeq2 normalized gene counts	DESeq2
GSE53380	25024434/ Wild-type (WT), KO animals, WT animals following novel-object recognition (NOR) and KO animals following NOR	Illumina HiSeq 2000, SE, 50 bp	GRCm38.p2, STAR 2.3.0	HTSeq	HTSeq raw gene counts	UQ
GSE65159	25693568/ animals 2 weeks and 6 weeks following the induction of p25 expression (mouse	Illumina HiSeq 2000, PE, 76bp	Bowtie	HTSeq	HTSeq raw gene counts	UQ

	model of Alzheimer's disease) an their respective controls					
GSE58343	25072471/ mRNA-seq of home cage and fear-conditioned animals. Includes pair-end (PE) and single-end (SE) technical replicates, RNA obtained from neuronal dendrites vs. soma, and RNA following ribosome immuno-precipitation versus supernatant of the same sample	Illumina HiSeq 2000, PE,100 bp, SE, 50bp	STAR(v2.1.1d)	Cufflinks/Cuff Diff	FPKM normalized gene counts	FPKM

*When raw counts were provided, UQ normalization was performed using EDASeq.

Additional Table 2. Sequencing and mapping statistics for OLM and FC RNA-seq samples.

See accompanying excel spreadsheet

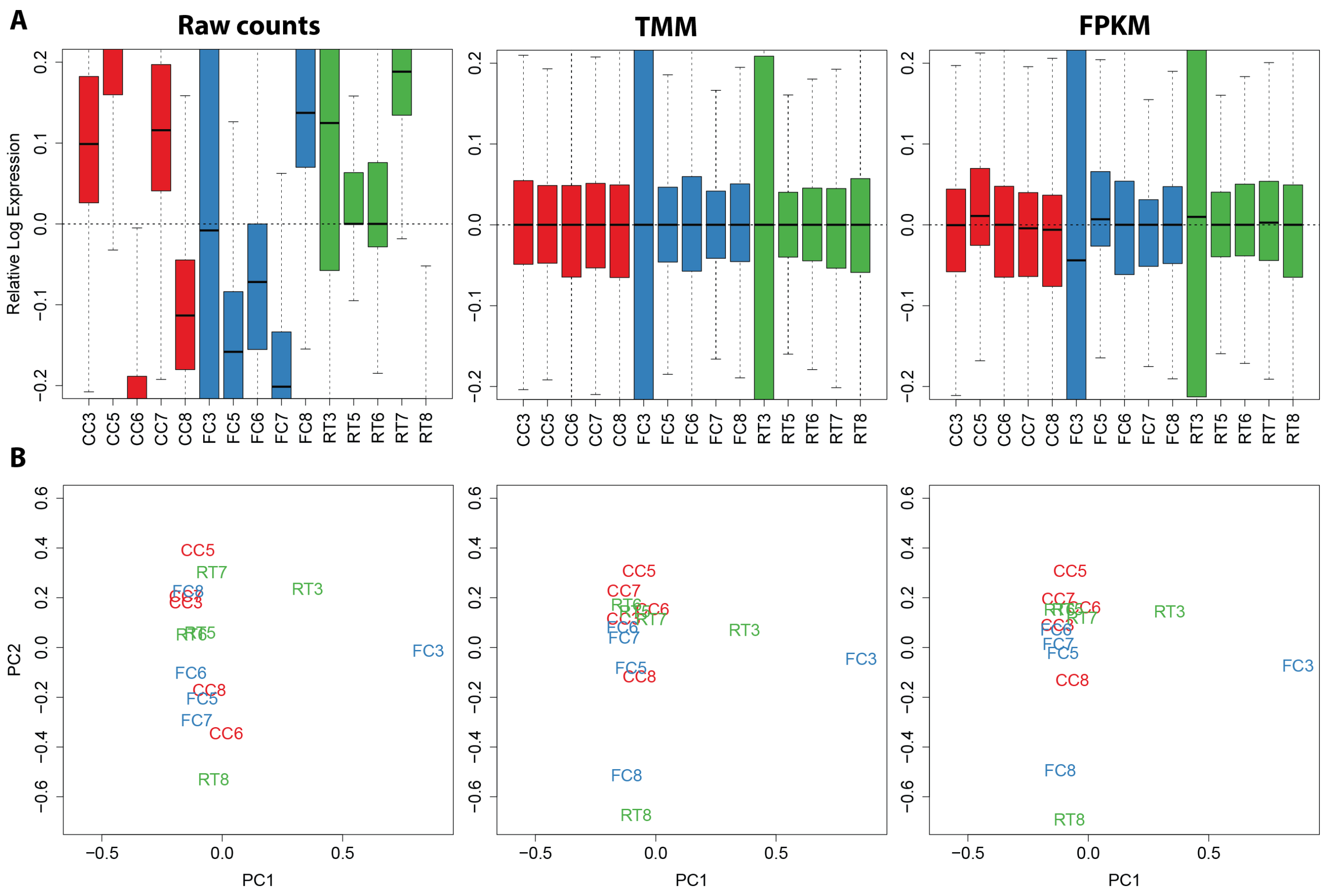

Additional Figure 1. **TMM and FPKM normalization methods do not correct unwanted variation in FC data**. In red control samples matched for time of day (CC), in blue samples obtained 30 minutes after memory acquisition (FC), in green samples obtained 30 minutes after memory retrieval (RT). Panel A) Relative log expression (RLE) plot of all samples for raw counts, following trimmed mean of M-values (TMM) and fragments per kilo-base of exon per million mapped fragments (FPKM). Panel B) Scatterplot of first two principal components (log-scaled, centered counts) for raw counts, TMM and FPKM normalization.

Additional Table 3. Negative and positive controls used in the study.

See accompanying excel spreadsheet

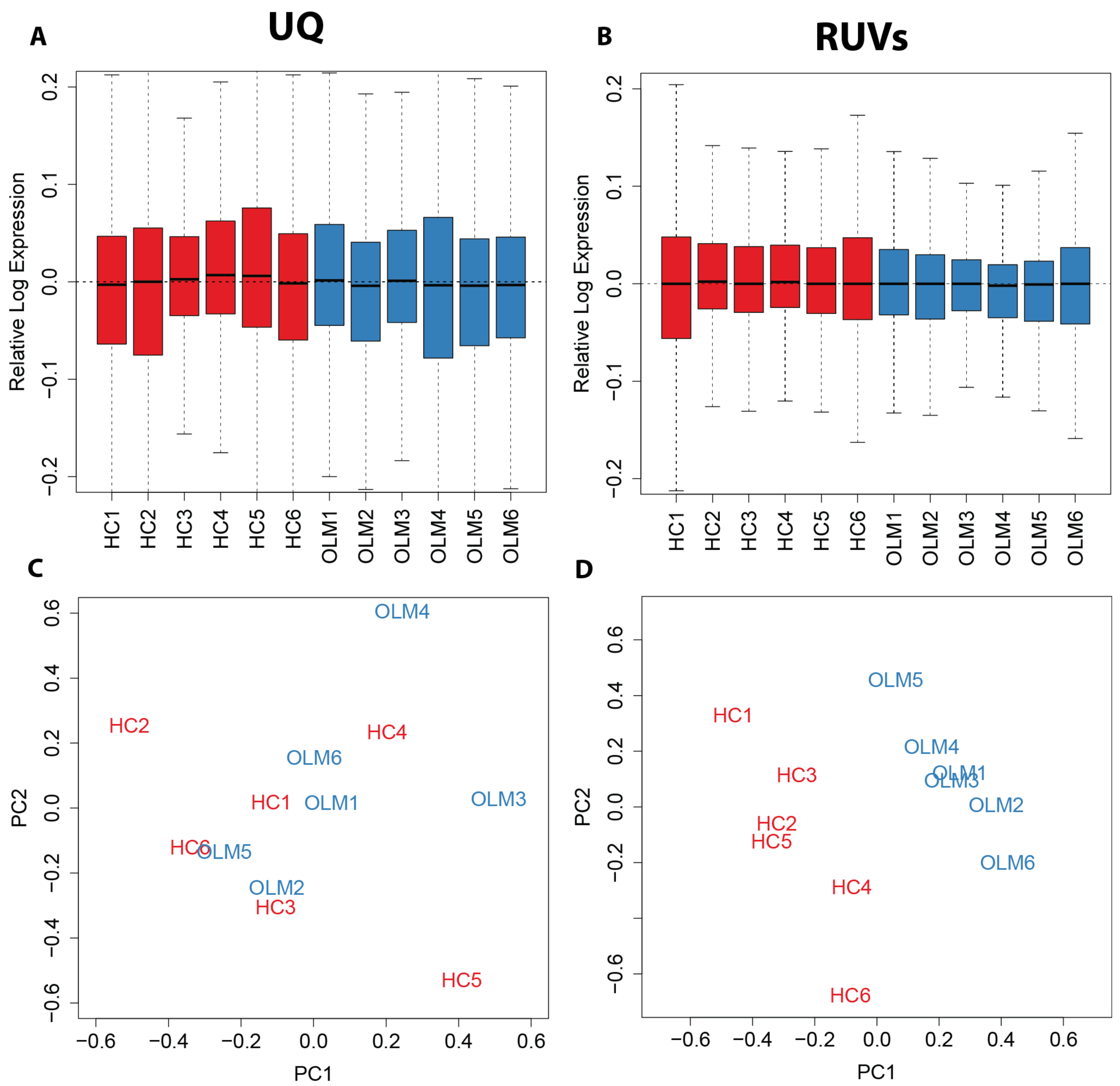

Additional Figure 2. **RUVs, but not UQ corrects unwanted variation in OLM data**. In red control samples matched for time of day (HC), in blue samples obtained 30 minutes after last training session following object location memory (OLM). A) Relative log expression (RLE) plot of all samples following traditional upper-quartile normalization (UQ). B) RLE plots following normalization with RUV using negative controls and samples (RUVs). C) Scatterplot of first two principal components (log-scaled, centered counts) following UQ normalization. The first two PCs explained 22.6% and 17.3% of the variance, respectively. D) Scatterplot of first two principal components following RUVs normalization. The first two PCs explained 23.7% and 16.5% of the variance, respectively.

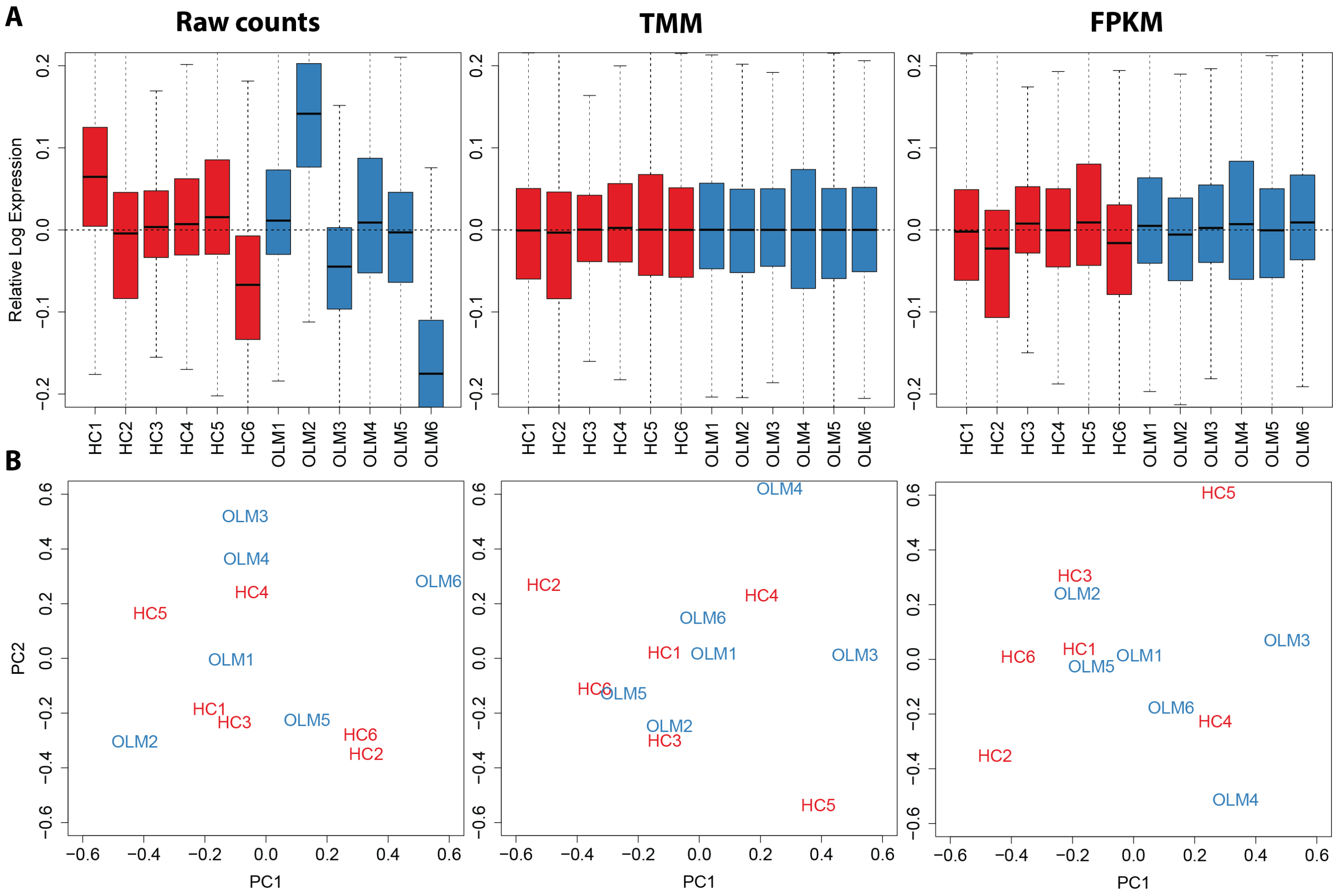

Additional Figure 3. TMM and FPKM normalization methods do not correct unwanted variation in OLM data. In red control samples matched for time of day (HC), in blue samples obtained 30 minutes after last training session following object location memory (OLM). Panel A) Relative log expression (RLE) plot of all samples for raw counts, following trimmed mean of M-values (TMM) and fragments per kilo-base of exon per million mapped fragments (FPKM). Panel B) Scatterplot of first two principal components (log-scaled, centered counts) for raw counts, TMM and FPKM normalization.

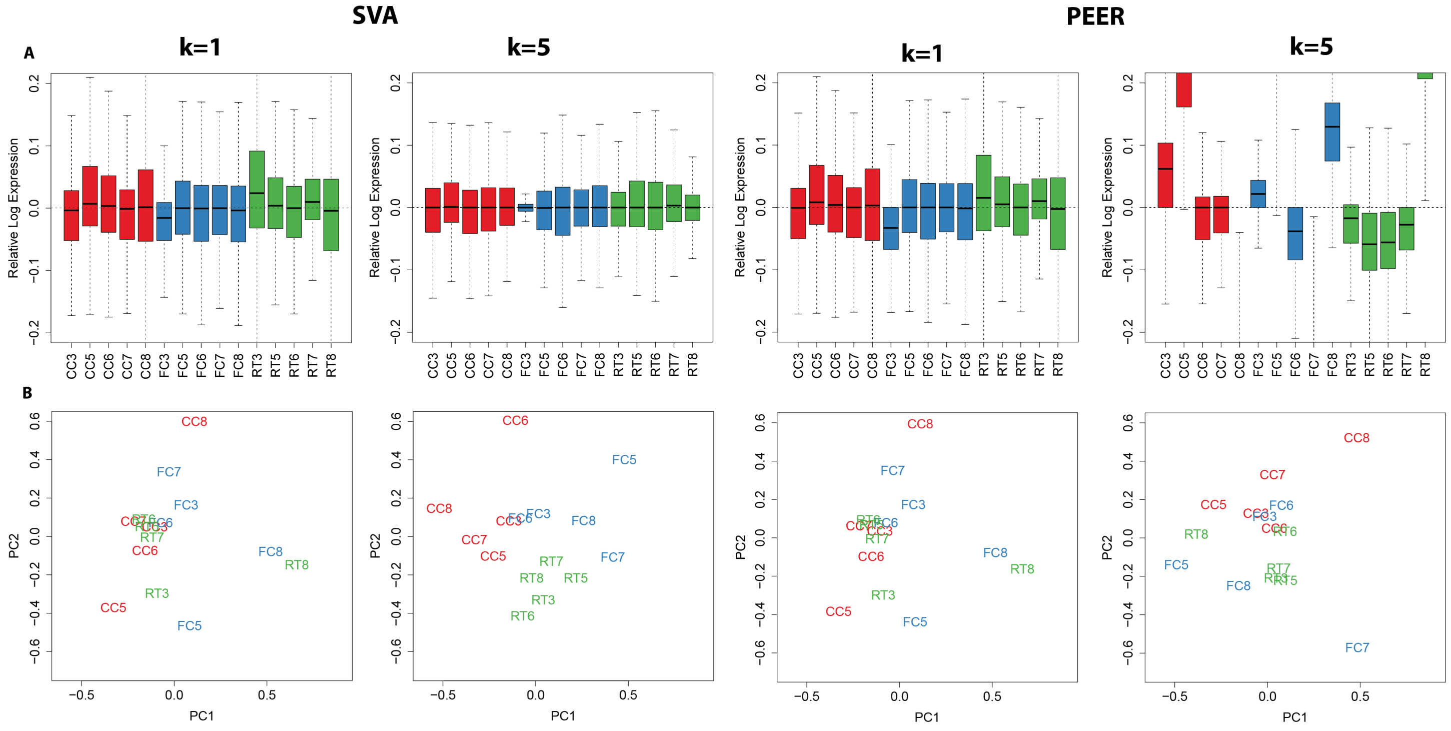

Additional Figure 4. **SVA and PEER normalization in FC data**. In red control samples matched for time of day (CC), in blue samples obtained 30 minutes after memory acquisition (FC), in green samples obtained 30 minutes after memory retrieval (RT). Panel A) Relative log expression (RLE) plot of all samples following normalization using SVA (unsupervised, n.sv=1) and PEER (k=1). Panel B) Scatterplot of first two principal components (log-scaled, centered counts) following normalization using SVA (unsupervised, n.sv=1) and PEER (k=1). The first two PCs of the SVA normalized data explained 19.7% and 13.1% of the variance, respectively. The first two PCs of the PEER normalized data explained 18.0% and 11.5% of the variance, respectively. As for RUV, we defined normalized expression by regressing out the estimated factors from the original data (Risso et al., 2014). SVA normalization was performed using R/Bioconductor package sva (v. 3.12.0). PEER normalization was performed using R package peer (v. 1.0).

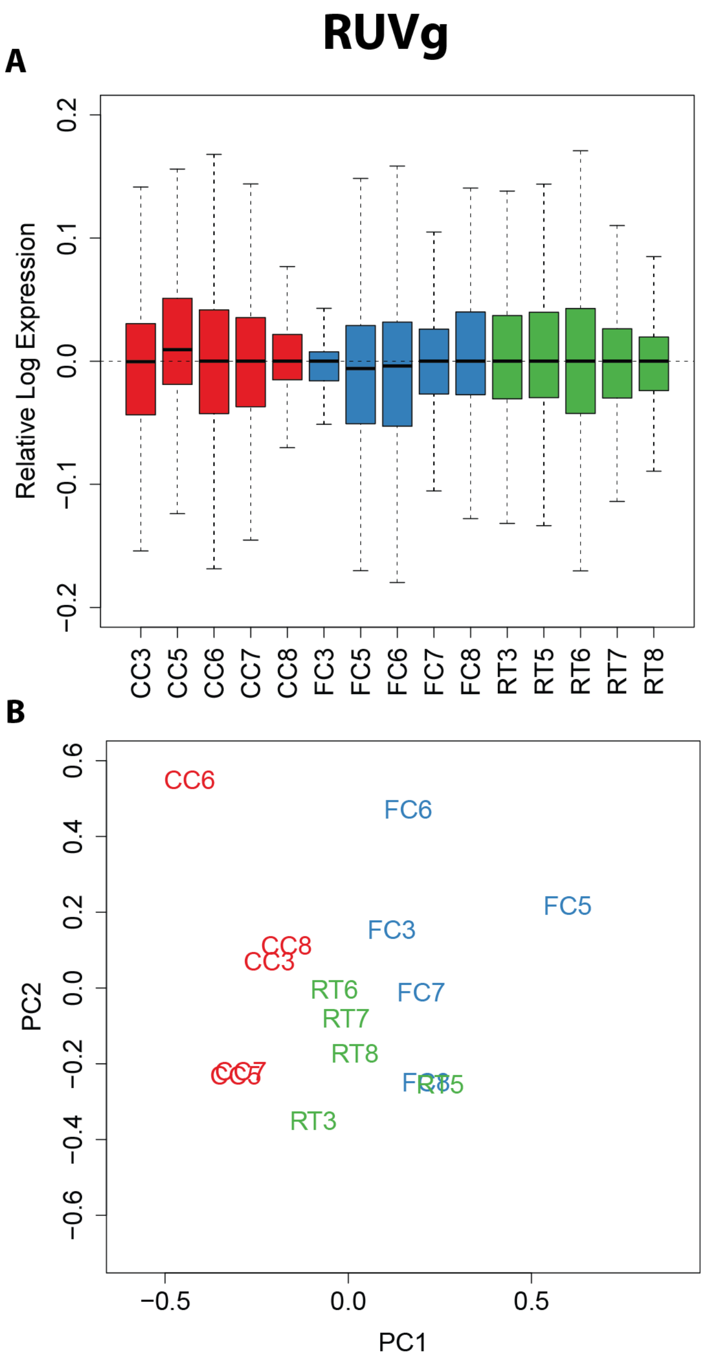

Additional Figure 5. **RUVg corrects unwanted variation in FC data**. In red control samples matched for time of day (CC), in blue samples obtained 30 minutes after memory acquisition (FC), in green samples obtained 30 minutes after memory retrieval (RT). A) Relative log expression (RLE) and B) Scatterplot of first two principal components following RUVg (RUV with control genes, without control samples) normalization.

RUVall

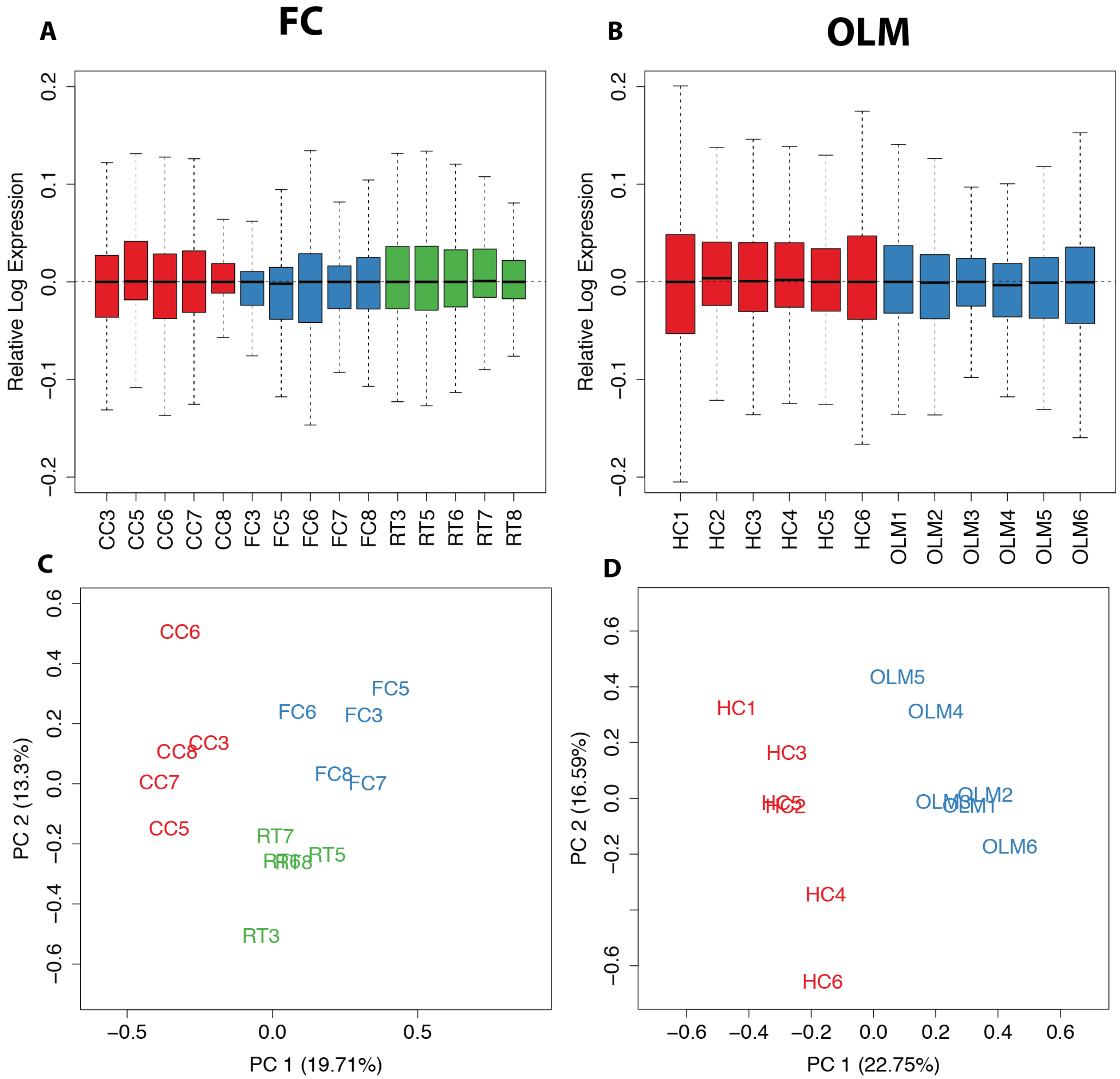

Additional Figure 6. **RUVall corrects unwanted variation in FC and OLM data**. In red control samples matched for time of day (CC), in blue samples obtained 30 minutes after memory acquisition (FC), in green samples obtained 30 minutes after memory retrieval (RT). A and B: Relative log expression (RLE) plots. C and D: Scatterplot of first two principal components following RUVall (RUVs with all genes as negative controls) normalization.

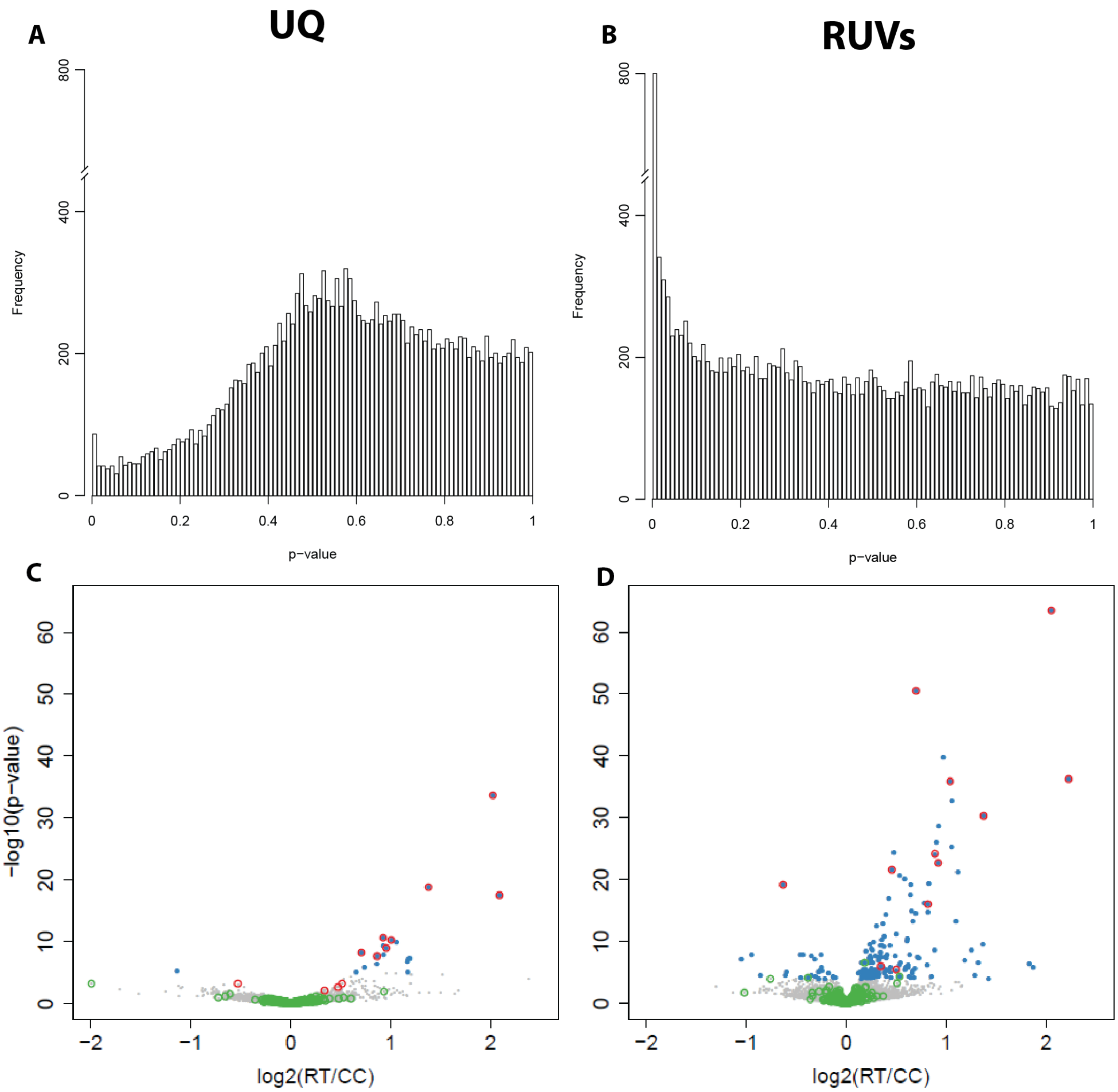

Additional Figure 7. **Normalization impacts differential expression after memory retrieval**. A) Distribution of edgeR p-values (uncorrected) for tests of differential expression between RT and CC samples following UQ normalization B) Distribution of edgeR p-values (uncorrected) for tests of differential expression between following RUVs normalization. C) Volcano plot of differential expression (-log10 p-value vs log fold change) of UQ normalized samples D) Volcano plot of differential expression of RUVs normalized samples. Genes with and FDR <0.01 are highlighted in blue. Positive controls are highlighted in red, negative controls are highlighted in green (**Additional Table 3**).

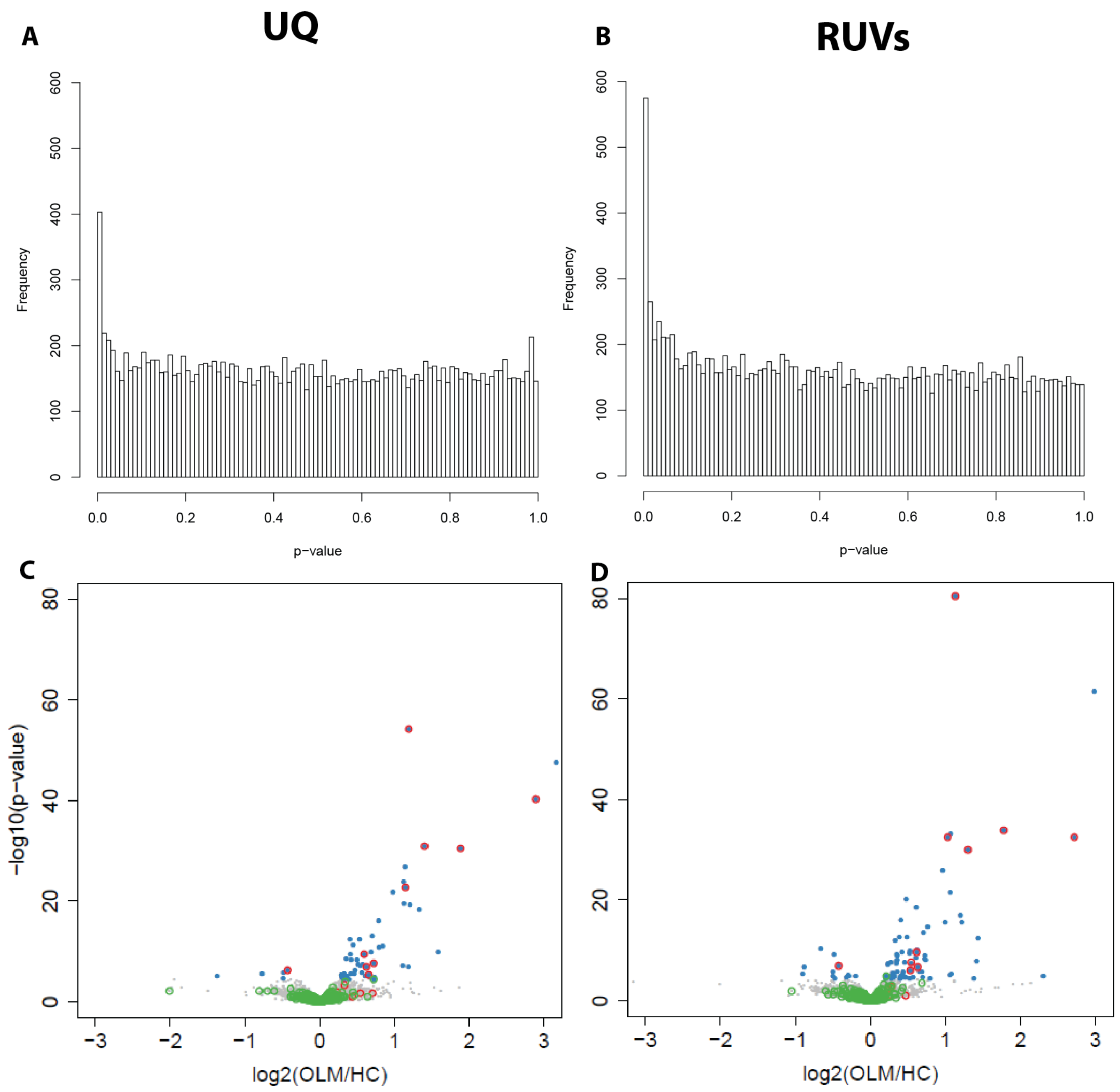

Additional Figure 8. **Normalization impacts differential expression after object location memory**. A) Distribution of edgeR p-values (uncorrected) for tests of differential expression between OLM and HC samples following UQ normalization B) Distribution of edgeR p-values (uncorrected) for tests of differential expression between following RUVs normalization. C) Volcano plot of differential expression (-log10 p-value vs log fold change) of UQ normalized samples D) Volcano plot of differential expression of RUVs normalized samples. Genes with and FDR <0.01 are highlighted in blue. Positive controls are highlighted in red, negative controls are highlighted in green (**Additional Table 3**).

Additional Table 4. Lists of differentially expressed genes (FDR <0.01) for all pairwise comparisons reported (FC vs. CC, RT vs. CC and OLM vs. HC) using UQ or RUVs normalization independently on the datasets obtained from different laboratories.

See accompanying excel spreadsheet.

A UQ-normalization

Upregulated

	genes	Enriched pathways
**OLM vs HC**	53	MAPK
**FC vs CC**	32	MAPK

Downregulated

	genes	Enriched pathways
**OLM vs HC**	5	None
**FC vs CC**	2	None

B RUV-normalization

Upregulated

	genes	Enriched pathways
**OLM vs HC**	72	MAPK
**FC vs CC**	237	MAPK, T-cell receptor signaling, Toll-like receptor signaling GnRH signaling, Insulin signaling

Downregulated

	genes	Enriched pathways
**OLM vs HC**	12	None
**FC vs CC**	166	GnRH signaling, Ca signaling, Melanogenesis, RIG-I receptor signaling

Additional Figure 9. **Quantitative and qualitative effects of the choice of normalization method in gene expression changes following FC and OLM**. A) Number of genes and enriched KEGG pathways for OLM or FC relative to their respective controls following UQ normalization. UQ normalization leads to inferring housekeeping genes as differentially expressed. B) Number of genes and enriched KEGG pathways for OLM or FC relative to their respective controls following RUVs normalization.

Additional Table 5. Lists of differentially expressed genes (FDR <0.01) for all comparisons reported (HC+CC vs. FC, HC+CC vs. OLM, FC+OLM vs. CC+HC, FC vs. OLM) using UQ or RUVs normalization on the combined analysis of datasets obtained from different laboratories.

See accompanying excel spreadsheet.

Additional File 1. Tutorial of implementation of RUV-seq normalization on FC, RT and OLM data (requires additional input files).

See accompanying pdf.

Source code and input files can be downloaded from the GiTHub repository:

github.com/drisso/peixoto2015_tutorial

Bellow is a list of the input files required to run the tutorial:

- Peixoto_CC_FC_RT.txt
- Peixoto_NegativeControls.txt
- Peixoto_positive_controls.txt
- Peixoto_FC_array_combined.txt
- Peixoto_OLM_HC.txt

Additional File 2. Tutorial of implementation of RUV-seq normalization on public datasets (requires additional input files).

Source code and input files can be downloaded from the GiTHub repository:

github.com/drisso/peixoto2015_tutorial

Bellow is a list of the input files required to run the tutorial:

- GSE60261.txt : Count matrix for GSE60261dataset
- GSE60262.txt : Count matrix for GSE60262 dataset
- GSE58797.txt: Count matrix for GSE58797 dataset
- GSE61915.txt: Count matrix for GSE61915 dataset
- GSE53380.txt: Count matrix for GSE53380 dataset
- GSE65159.txt: Count matrix for GSE65159 dataset
- GSE58343.txt: Count matrix for GSE58343 dataset

Additional references

1. Fiorenza, A., Lopez-Atalaya, J.P., Rovira, V., Scandaglia, M., Geijo-Barrientos, E. and Barco, A. (2015) Blocking miRNA Biogenesis in Adult Forebrain Neurons Enhances Seizure Susceptibility, Fear Memory, and Food Intake by Increasing Neuronal Responsiveness. *Cerebral cortex*.
2. Zovkic, I.B., Paulukaitis, B.S., Day, J.J., Etikala, D.M. and Sweatt, J.D. (2014) Histone H2A.Z subunit exchange controls consolidation of recent and remote memory. *Nature*, **515**, 582-586.
3. Stilling, R.M., Benito, E., Gertig, M., Barth, J., Capece, V., Burkhardt, S., Bonn, S. and Fischer, A. (2014) De-regulation of gene expression and alternative splicing affects distinct cellular pathways in the aging hippocampus. *Frontiers in cellular neuroscience*, **8**, 373.
4. Stilling, R.M., Ronicke, R., Benito, E., Urbanke, H., Capece, V., Burkhardt, S., Bahari-Javan, S., Barth, J., Sananbenesi, F., Schutz, A.L. *et al.* (2014) K-Lysine acetyltransferase 2a regulates a hippocampal gene expression network linked to memory formation. *The EMBO journal*, **33**, 1912-1927.
5. Gjoneska, E., Pfenning, A.R., Mathys, H., Quon, G., Kundaje, A., Tsai, L.H. and Kellis, M. (2015) Conserved epigenomic signals in mice and humans reveal immune basis of Alzheimer's disease. *Nature*, **518**, 365-369.
6. Ainsley, J.A., Drane, L., Jacobs, J., Kittelberger, K.A. and Reijmers, L.G. (2014) Functionally diverse dendritic mRNAs rapidly associate with ribosomes following a novel experience. *Nature communications*, **5**, 4510.
7. Vogel-Ciernia, A., Matheos, D.P., Barrett, R.M., Kramar, E.A., Azzawi, S., Chen, Y., Magnan, C.N., Zeller, M., Sylvain, A., Haettig, J. *et al.* (2013) The neuron-specific chromatin regulatory subunit BAF53b is necessary for synaptic plasticity and memory. *Nature neuroscience*, **16**, 552-561.
