# Supplementary material for: How data analysis affects power, reproducibility and biological insight of RNA-seq studies in complex datasets
Source: Nucleic Acids Res. 2015 Jul 21;43(16):7664–74. doi: 10.1093/nar/gkv736 (PMC4652761; doi:10.1093/nar/gkv736)
Supplement: SUPPLEMENTARY DATA [file supp_gkv736_nar-01134-survey-d-2015-File011.pdf]

How data analysis affects power, reproducibility and biological insight of RNA-seq studies in complex datasets

Lucia Peixoto, Davide Risso, Shane G. Poplawski, Mathieu, E. Wimmer, Terence P. Speed, Marcelo A. Wood and Ted Abel

Contents

1 Preliminaries **1**
1.1 Data pre-processing 1
1.2 Positive and negative controls 2
1.3 Bioconductor packages 2

2 Fear Conditioning (FC) data **2**
2.1 Normalization 3
2.2 Differential expression 5

3 Object Location Memory (OLM) data **13**
3.1 Normalization 14
3.2 Differential expression 16

4 Combined Analysis **19**
4.1 Normalization 19
4.2 Differential expression 22

5 Comparison with microarray data **26**

6 Tuning parameters **28**
6.1 Number of factors of unwanted variation (k) 29
6.2 Set of negative control genes 32

7 Session Info **33**

References **35**

1 Preliminaries

1.1 Data pre-processing

1.1.1 RNA-seq data

The "raw" sequencing data are available in GEO with the accession number GSE63412 for the fear conditioning (FC) experiment and GSE44229 for the object location memory (OLM) experiment.

Reads were mapped to the mouse genome (mm9) using GMAP/GSNAP (Wu and Watanabe 2005). Only unique and concordant mapped reads were subsequently used for feature quantitation. Ensembl (Flicek et al. 2014) (release 65) gene counts were obtained using HTSeq (Anders, Pyl, and Huber 2015) (v. 0.6.1).

Peixoto_Additional_inputtext.zip is a ZIP archive that contains all the files needed to run this tutorial. For the remainder of the document we assume that the reader has extracted the archive into the working directory.

Peixoto_CC_FC_RT.txt contains the FC read counts and **Peixoto_OLM_HC.txt** contains the OLM read counts used in the subsequent analyses.

1.1.2 Microarray data

The "raw" microarray data are available in GEO with the accession number GSE50423. See the GEO submission for details on the pre-processing.

We mapped the Affymetrix probe IDs to ENSEMBL gene IDs. We retained only the genes with a one-to-one mapping. **Peixoto_FC_array_combined.txt** contains the combined dataset used for the ranking analysis of Figure 5.

1.2 Positive and negative controls

Peixoto_NegativeControls.txt contains a list of negative control genes, i.e., genes that are not influenced by the biological effect of interest (see the main text for details). **Peixoto_positive_controls.txt** contains a list of positive control genes, i.e., genes known to be differentially expressed with respect to the biological effect of interest (see the main text for details).

1.3 Bioconductor packages

Remove Unwanted Variation (RUV) normalization is implemented in the *RUVSeq* package. Upper-quartile (UQ) normalization is implemented in the *EDASeq* package. To generate the figures, we have used the *plotRLE* and *plotPCA* functions implemented in *EDASeq* and the *CATplot* function implemented in the *ffpe* package. The differential expression (DE) analysis was carried out with *edgeR* for the RNA-seq data and with *limma* for the microarray data. See "Session Info" for the package versions used in this document.

```
library(limma)
library(edgeR)
library(EDASeq)
library(RUVSeq)
library(ffpe)
library(RColorBrewer)
```

2 Fear Conditioning (FC) data

After reading the FC counts and the positive and negative control genes in R, we filter out the non expressed genes.

```
fc <- read.table("Peixoto_Additional_inputtext/Peixoto_CC_FC_RT.txt", row.names = 1,
    header = TRUE)
negControls <- read.table("Peixoto_Additional_inputtext/Peixoto_NegativeControls.txt",
    sep = "\t", header = TRUE, as.is = TRUE)
positive <- read.table("Peixoto_Additional_inputtext/Peixoto_positive_controls.txt",
    as.is = TRUE, sep = "\t", header = TRUE)

x <- as.factor(rep(c("CC", "FC", "RT"), each = 5))
names(x) <- colnames(fc)

filter <- apply(fc, 1, function(x) length(x[which(x > 10)]) > 5)
filtered <- as.matrix(fc)[filter, ]

negCon <- intersect(negControls[, 2], rownames(filtered))
FCup <- intersect(positive[positive[, 3] == "UP", 1], rownames(filtered))
FCdown <- intersect(positive[positive[, 3] == "DOWN", 1], rownames(filtered))
RTup <- intersect(positive[positive[, 4] == "UP", 1], rownames(filtered))
RTdown <- intersect(positive[positive[, 4] == "DOWN", 1], rownames(filtered))
```

```
colors <- brewer.pal(9, "Set1")
colLib <- colors[x]
```

2.1 Normalization

2.1.1 UQ normalization

The *betweenLaneNormalization* function of *EDASeq* implements UQ normalization. We can then use the *plotRLE* and *plotPCA* functions of *EDASeq* to explore the normalized data.

```
uq <- betweenLaneNormalization(filtered, which = "upper")

plotRLE(uq, col = colLib, outline = FALSE, las = 3, ylim = c(-0.2, 0.2), ylab = "Relative Log Expression",
    cex.axis = 1, cex.lab = 1)
```

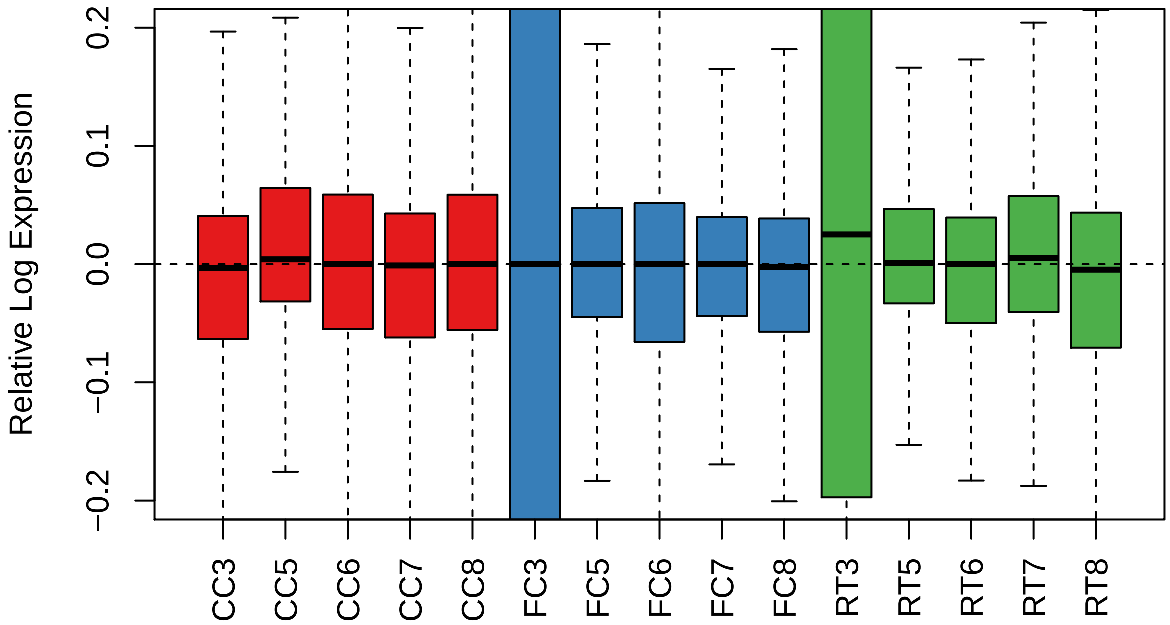


```
plotPCA(uq, col = colLib, cex = 1, cex.axis = 1, cex.lab = 1, xlim = c(-0.6, 0.9),
    ylim = c(-0.7, 0.6))
```

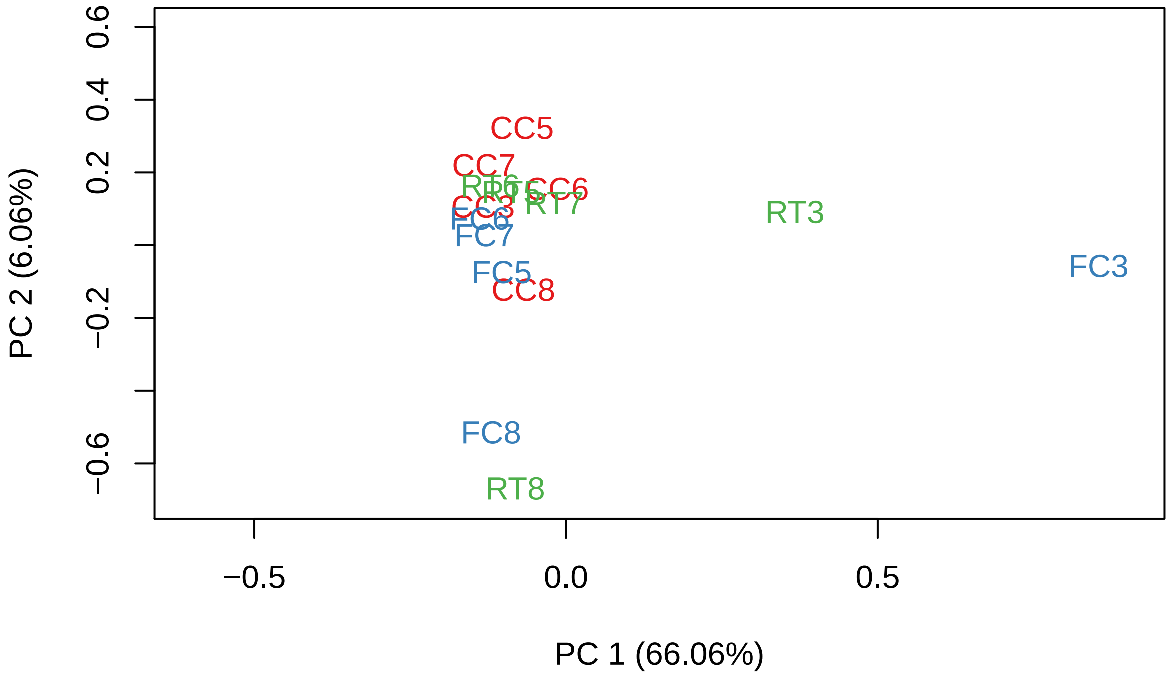

2.1.2 RUV normalization

Analogously, we can use the *RUVs* function of *RUVSeq* to normalize the data with RUV. Note that *RUVs* uses replicate samples and negative control genes to normalize the data. We use the biological replicates and a list of negative control genes obtained from a recent microarray study. Here, we use *RUVs* on UQ-normalized data, see (Risso et al. 2014) for additional details.

The information about the replicates is contained in the matrix *groups*, which has as many rows as the number of replicate groups and as many columns as the number of replicates in each group (see the *RUVSeq* package manual for additional details).

```
groups <- matrix(data = c(1:5, 6:10, 11:15), nrow = 3, byrow = TRUE)
groups
```

```
##      [,1] [,2] [,3] [,4] [,5]
## [1,]    1    2    3    4    5
## [2,]    6    7    8    9   10
## [3,]   11   12   13   14   15
```

```
s <- RUVs(uq, negCon, k = 5, groups)

plotRLE(s$normalizedCounts, col = colLib, outline = FALSE, las = 3, ylim = c(-0.2,
    0.2), ylab = "Relative Log Expression", cex.axis = 1, cex.lab = 1)
```

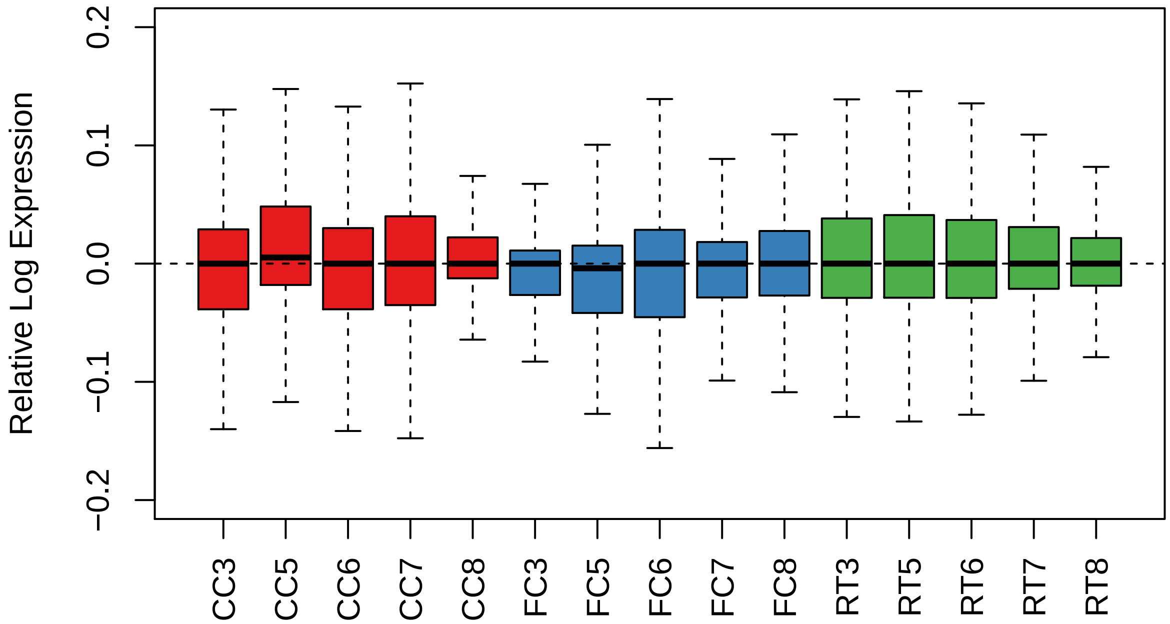


```
plotPCA(s$normalizedCounts, col = colLib, cex = 1, cex.axis = 1, cex.lab = 1, xlim = c(-0.6,
    0.9), ylim = c(-0.7, 0.6))
```

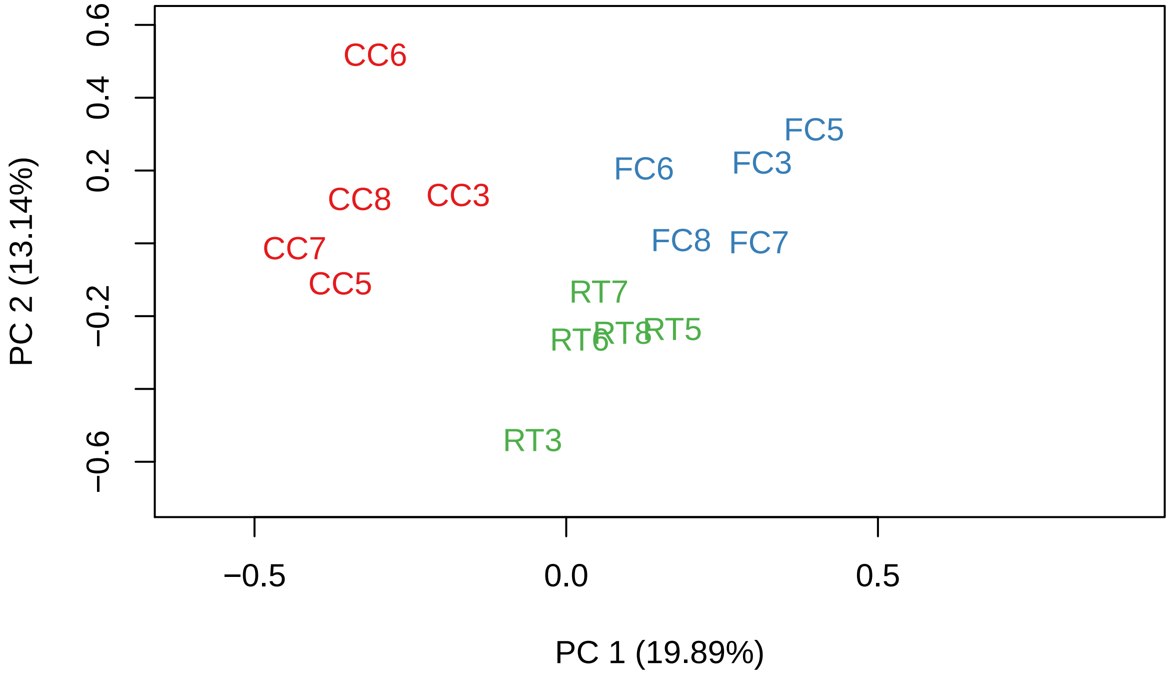

2.2 Differential expression

Next, we evaluate the impact of the normalization on differential expression. Note that it is preferable to model the original read counts, adding the normalization factors to the differential expression model, rather than modeling the normalized counts. See (Risso et al. 2014) for a discussion.

Here, we consider *edgeR*, but other count-based models, such as *DESeq* can also be used.

2.2.1 UQ normalization

We model the counts with a generalized linear model (GLM), using the group variable x as covariate. The design matrix is hence

```
design <- model.matrix(~x)
design
```

```
##    (Intercept) xFC xRT
## 1            1   0   0
## 2            1   0   0
## 3            1   0   0
## 4            1   0   0
## 5            1   0   0
## 6            1   1   0
## 7            1   1   0
## 8            1   1   0
## 9            1   1   0
## 10           1   1   0
## 11           1   0   1
## 12           1   0   1
## 13           1   0   1
## 14           1   0   1
## 15           1   0   1
## attr(,"assign")
## [1] 0 1 1
## attr(,"contrasts")
## attr(,"contrasts")$x
## [1] "contr.treatment"
```

We compute the UQ normalization factors, estimate the dispersion parameters and fit the GLM.

```
y <- DGEList(counts = filtered, group = x)
y <- calcNormFactors(y, method = "upperquartile")

y <- estimateGLMCommonDisp(y, design, verbose = TRUE)
```

```
## Disp = 0.04112 , BCV = 0.2028
```

```
y <- estimateGLMTagwiseDisp(y, design)

fit <- glmFit(y, design)
```

FC vs CC. First, we compare the expression levels after training (FC) to those of the controls (CC).

We expect the distribution of the p-values to be uniform for the majority of the non DE genes, with a spike at zero corresponding to the DE genes.

```
lrt <- glmLRT(fit, coef = 2)
topUQFC <- topTags(lrt, n = Inf)$table

hist(topUQFC$PValue, main = "", xlab = "p-value", breaks = 100, ylim = c(0, 1400))
```

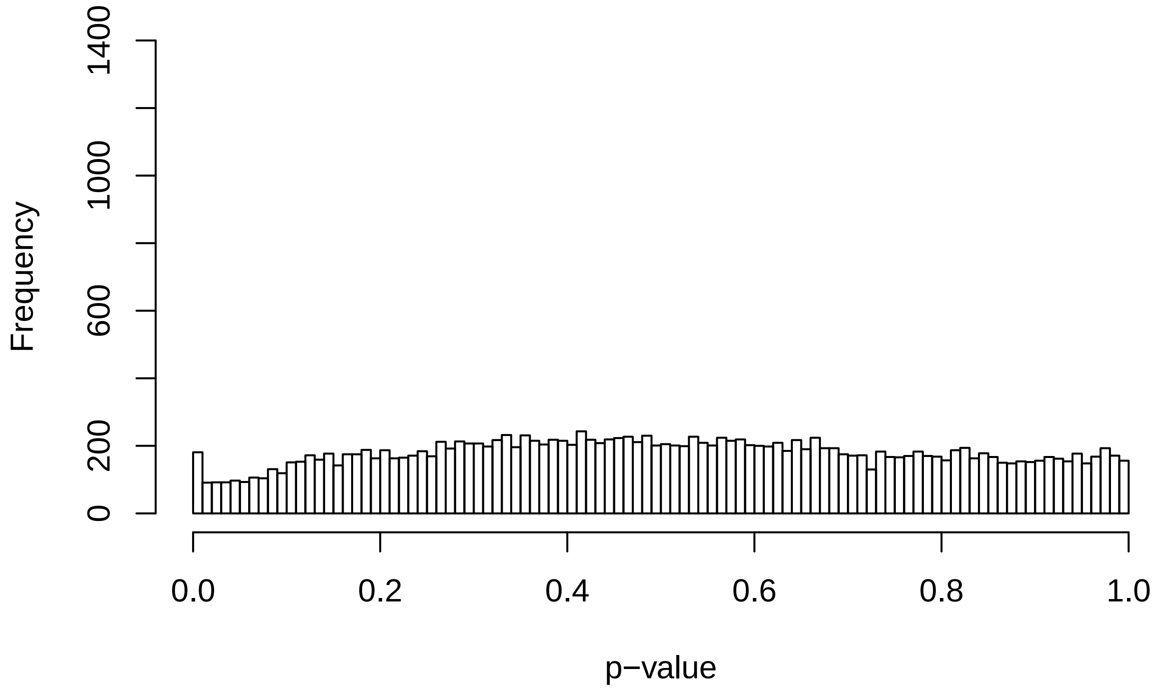

The histogram of the *p*-value corresponding to UQ normalization is not ideal.

Additionally, we can look at a "volcano plot," in which we plot the negative log *p*-values vs. the log-fold-change. We expect to see the positive controls (red circles) as high as possible and the negative controls (green circles) as close to zero as possible. The blue points correspond to genes called DE at an FDR of 0.01.

```
plot(topUQFC[, 1], -log10(topUQFC$PValue), pch = 20, col = "gray", cex = 0.5, ylab = "-log10(p-value)",
    xlab = "log2(FC/CC)", ylim = c(0, 85), xlim = c(-2, 4), cex.lab = 1, cex.axis = 1)
de <- rownames(topUQFC[topUQFC$FDR <= 0.01, ])
points(topUQFC[de, 1], -log10(topUQFC[de, "PValue"]), pch = 20, col = colors[2],
    cex = 1)
points(topUQFC[FCup, 1], -log10(topUQFC[FCup, "PValue"]), pch = 1, col = colors[1],
    cex = 1, lwd = 2)
points(topUQFC[FCdown, 1], -log10(topUQFC[FCdown, "PValue"]), pch = 1, col = colors[1],
    cex = 1, lwd = 2)
points(topUQFC[negCon, 1], -log10(topUQFC[negCon, "PValue"]), pch = 1, col = colors[3],
    cex = 1, lwd = 2)
```

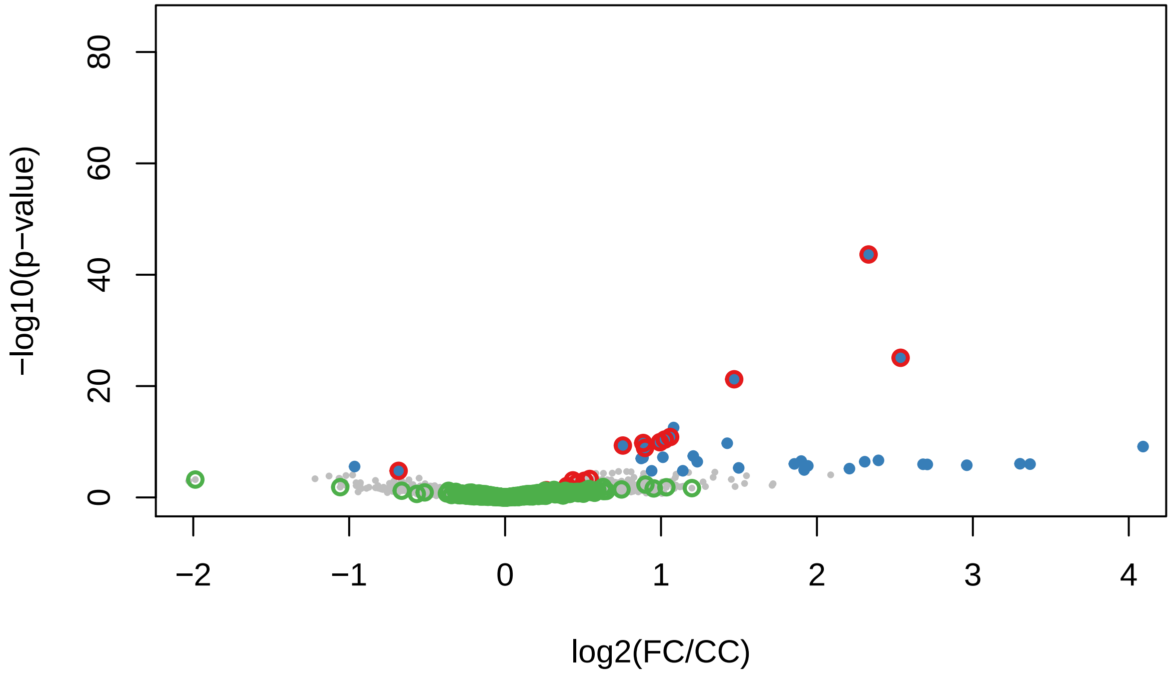

RT vs. CC. We also compare the expression levels after retrieval of the memory (RT) to those of the controls (CC).

```
lrt <- glmLRT(fit, coef = 3)
topUQRT <- topTags(lrt, n = Inf)$table

hist(topUQRT$PValue, main = "", xlab = "p-value", breaks = 100, ylim = c(0, 900))
```

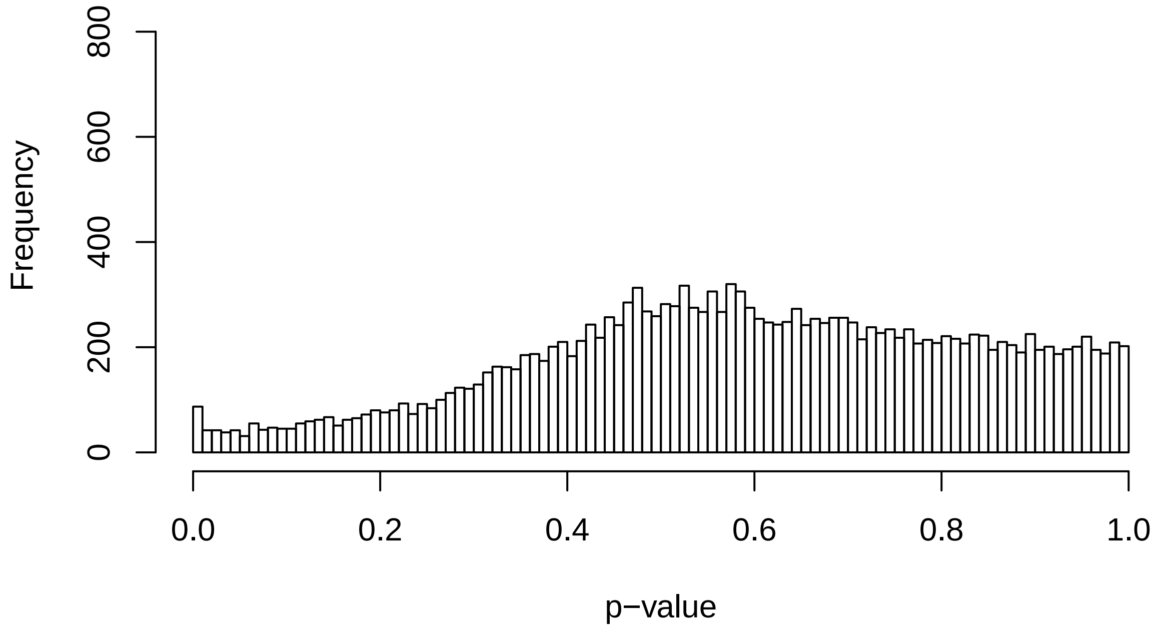


```
plot(topUQRT[, 1], -log10(topUQRT$PValue), pch = 20, col = "gray", cex = 0.5, ylab = "-log10(p-value)",
    xlab = "log2(RT/CC)", ylim = c(0, 65), xlim = c(-2, 2.5), cex.lab = 1, cex.axis = 1)
de <- rownames(topUQRT[topUQRT$FDR <= 0.01, ])
points(topUQRT[de, 1], -log10(topUQRT[de, "PValue"]), pch = 20, col = colors[2],
    cex = 1)
points(topUQRT[RTup, 1], -log10(topUQRT[RTup, "PValue"]), pch = 1, col = colors[1],
```

```
    cex = 1, lwd = 2)
points(topUQRT[RTdown, 1], -log10(topUQRT[RTdown, "PValue"]), pch = 1, col = colors[1],
    cex = 1, lwd = 2)
points(topUQRT[negCon, 1], -log10(topUQRT[negCon, "PValue"]), pch = 1, col = colors[3],
    cex = 1, lwd = 2)
```

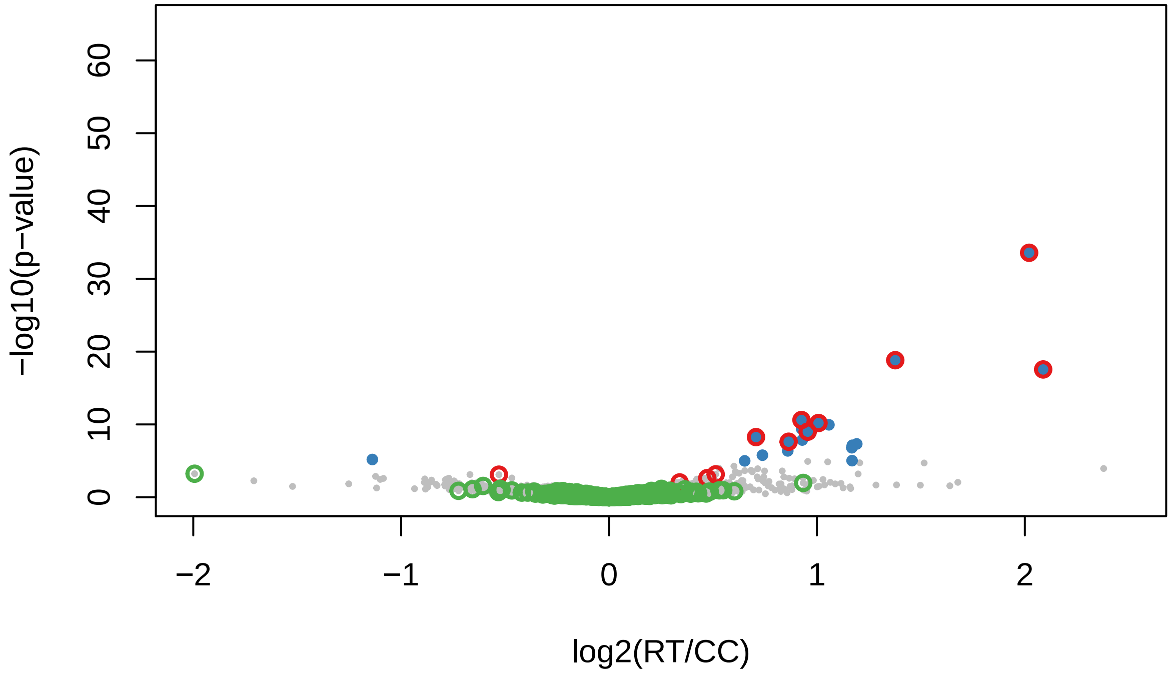

2.2.2 RUV normalization

In the RUV approach, we consider the factors of unwanted variation as additional covariates in the GLM (see *risso2014ruv* for details).

```
design <- model.matrix(~x + s$W)
design
```

```
##    (Intercept) xFC xRT      s$WW_1     s$WW_2    s$WW_3    s$WW_4
## 1            1   0   0 0.04043165 -1.5178537 -2.470752 -5.188997
## 2            1   0   0 0.09967579 -1.6652696 -2.807996 -5.466711
## 3            1   0   0 0.15112914 -1.4226748 -2.543977 -5.526913
## 4            1   0   0 0.04354857 -1.6859244 -2.359658 -5.172585
## 5            1   0   0 0.10064759 -1.2120067 -1.728733 -5.907422
## 6            1   1   0 1.03616492 -1.4171129 -2.314199 -5.145315
## 7            1   1   0 0.04705958 -1.5635628 -2.750573 -5.892653
## 8            1   1   0 0.01638110 -1.6412488 -2.334876 -5.488086
## 9            1   1   0 0.02165814 -1.5675696 -2.040667 -5.285826
## 10           1   1   0 0.03511239 -1.0567208 -2.478183 -5.339183
## 11           1   0   1 0.54678220 -1.5385567 -2.514049 -5.999072
## 12           1   0   1 0.06766670 -1.7849279 -2.224247 -5.404268
## 13           1   0   1 0.03320416 -1.5453762 -2.302969 -5.237210
## 14           1   0   1 0.14742538 -1.6153858 -2.346276 -5.559301
## 15           1   0   1 0.07140432 -0.7276544 -2.603699 -5.384926
##       s$WW_5
## 1  -1.541330
```

```
## 2  -1.230319
## 3  -1.876568
## 4  -1.046862
## 5  -1.109745
## 6  -1.217443
## 7  -1.131867
## 8  -1.154119
## 9  -1.870579
## 10 -1.507956
## 11 -1.677098
## 12 -1.382377
## 13 -1.200236
## 14 -1.695319
## 15 -1.351004
## attr(,"assign")
## [1] 0 1 1 2 2 2 2 2
## attr(,"contrasts")
## attr(,"contrasts")$x
## [1] "contr.treatment"
```

We compute the UQ normalization factors, estimate the dispersion parameters and fit the GLM.

```
y <- DGEList(counts = filtered, group = x)
y <- calcNormFactors(y, method = "upperquartile")

y <- estimateGLMCommonDisp(y, design)
y <- estimateGLMTagwiseDisp(y, design)

fit <- glmFit(y, design)
```

FC vs CC. First, we compare the expression levels after training (FC) to those of the controls (CC).

```
lrt <- glmLRT(fit, coef = 2)
topRsFC <- topTags(lrt, n = Inf)$table

hist(topRsFC$PValue, main = "", xlab = "p-value", breaks = 100, ylim = c(0, 1400))
```

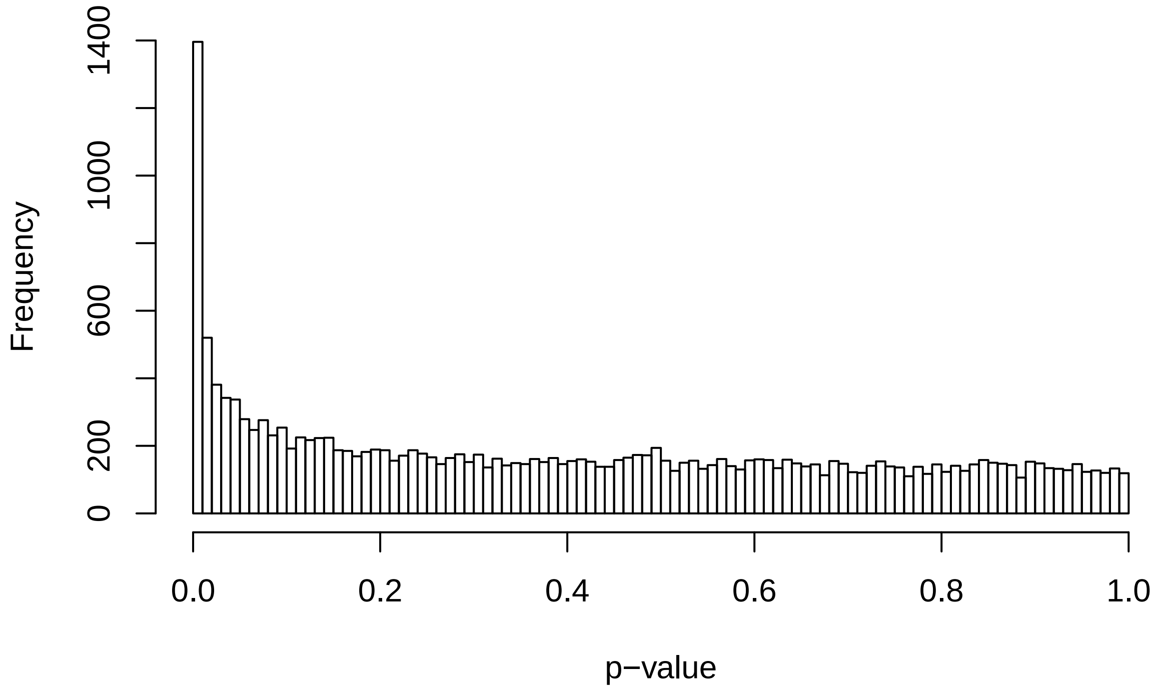


```
plot(topRsFC[, 1], -log10(topRsFC$PValue), pch = 20, col = "gray", cex = 0.5, ylab = "-log10(p-value)",
    xlab = "log2(FC/CC)", ylim = c(0, 85), xlim = c(-2, 4), cex.lab = 1, cex.axis = 1)
de <- rownames(topRsFC[topRsFC$FDR <= 0.01, ])
points(topRsFC[de, 1], -log10(topRsFC[de, "PValue"]), pch = 20, col = colors[2],
    cex = 1)
points(topRsFC[FCup, 1], -log10(topRsFC[FCup, "PValue"]), pch = 1, col = colors[1],
    cex = 1, lwd = 2)
points(topRsFC[FCdown, 1], -log10(topRsFC[FCdown, "PValue"]), pch = 1, col = colors[1],
    cex = 1, lwd = 2)
points(topRsFC[negCon, 1], -log10(topRsFC[negCon, "PValue"]), pch = 1, col = colors[3],
    cex = 1, lwd = 2)
```

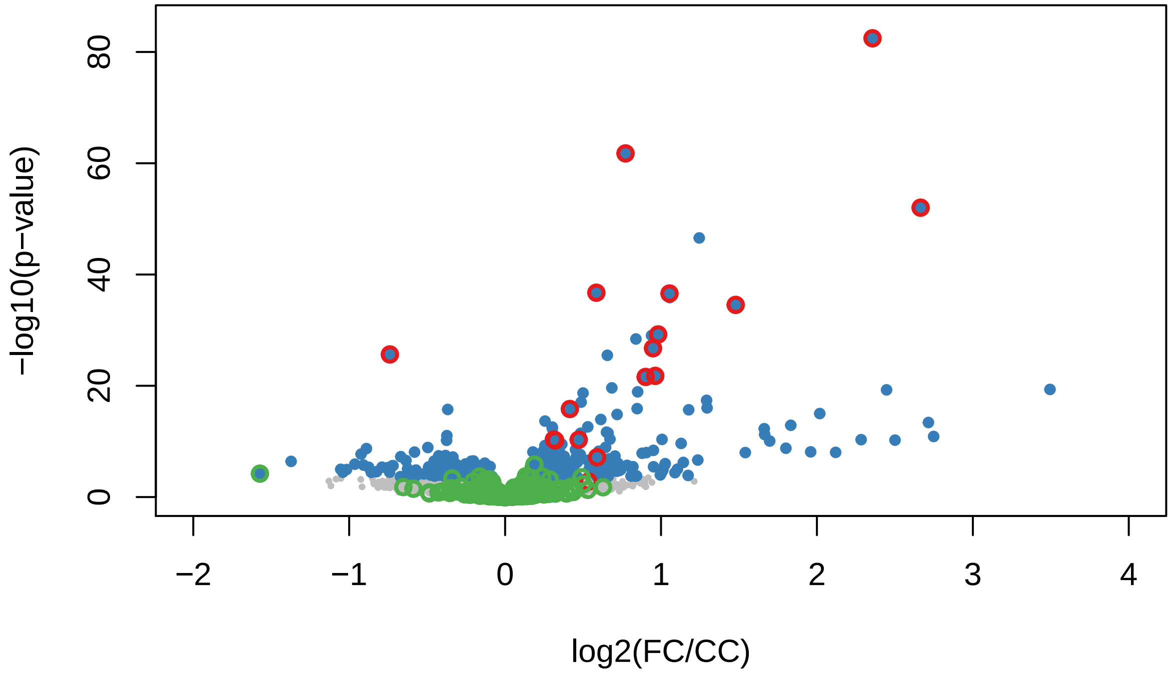

The histogram looks like expected and the volcano plot shows that we obtain smaller p-values for the positive controls, while the negative controls are still close to one (zero in the -log scale). We also detect more genes as DE, suggesting more power, although the DE genes might be a mixture of true and false positives.

RT vs. CC. We also compare the expression levels after retrieval of the memory (RT) to those of the controls (CC).

```
lrt <- glmLRT(fit, coef = 3)
topRsRT <- topTags(lrt, n = Inf)$table

hist(topRsRT$PValue, main = "", xlab = "p-value", breaks = 100, ylim = c(0, 900))
```

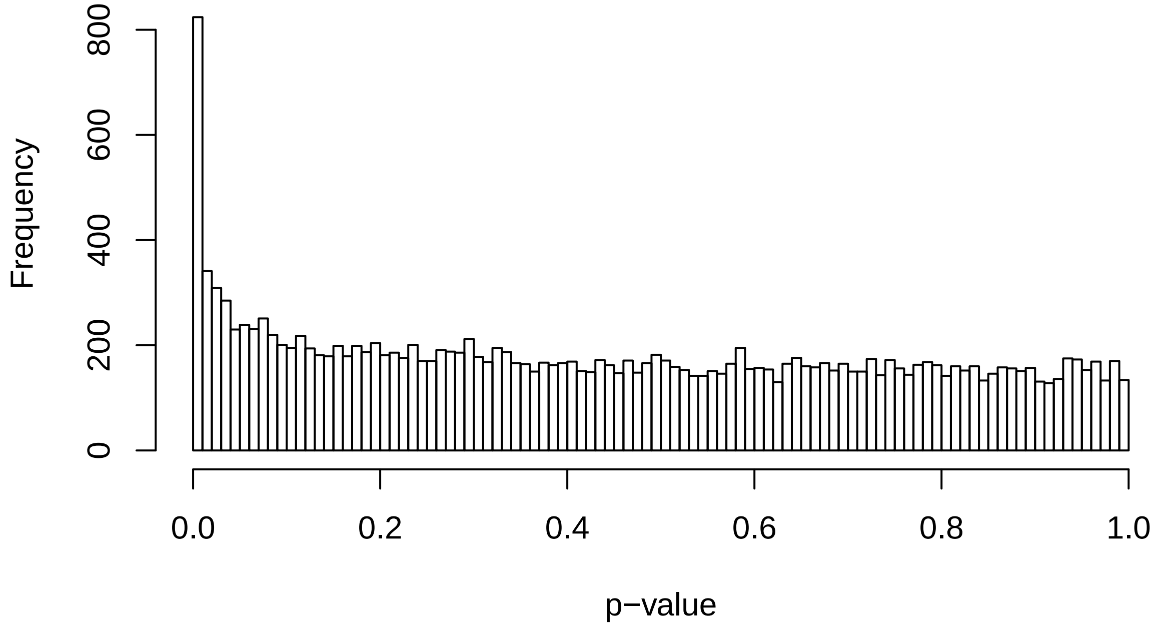


```
plot(topRsRT[, 1], -log10(topRsRT$PValue), pch = 20, col = "gray", cex = 0.5, ylab = "-log10(p-value)",
    xlab = "log2(RT/CC)", ylim = c(0, 65), xlim = c(-2, 2.5), cex.lab = 1, cex.axis = 1)
de <- rownames(topRsRT[topRsRT$FDR <= 0.01, ])
```

```
points(topRsRT[de, 1], -log10(topRsRT[de, "PValue"]), pch = 20, col = colors[2],
    cex = 1)
points(topRsRT[RTup, 1], -log10(topRsRT[RTup, "PValue"]), pch = 1, col = colors[1],
    cex = 1, lwd = 2)
points(topRsRT[RTdown, 1], -log10(topRsRT[RTdown, "PValue"]), pch = 1, col = colors[1],
    cex = 1, lwd = 2)
points(topRsRT[negCon, 1], -log10(topRsRT[negCon, "PValue"]), pch = 1, col = colors[3],
    cex = 1, lwd = 2)
```

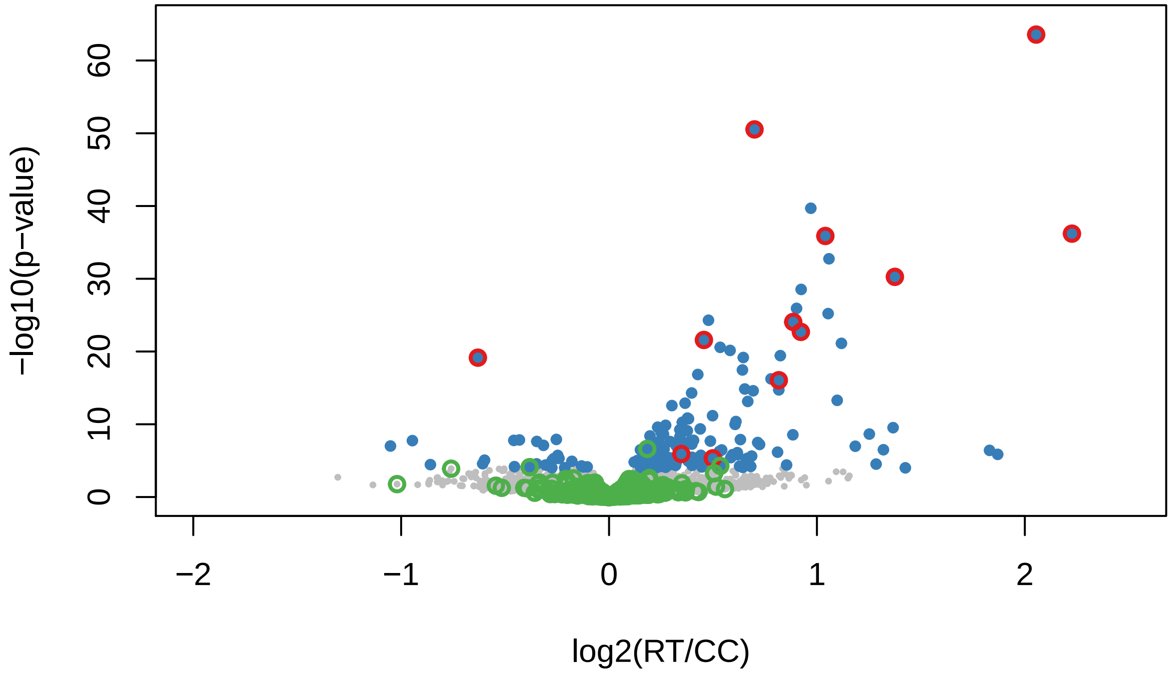

3 Object Location Memory (OLM) data

We next look at the OLM experiment. After reading the OLM counts into R, we filter out the non expressed genes.

```
olm <- read.table("Peixoto_Additional_inputtext/Peixoto_OLM_HC.txt", row.names = 1,
    header = TRUE)
stopifnot(all(rownames(olm) == rownames(fc)))

x <- as.factor(rep(c("HC", "OLM"), each = 6))
names(x) <- colnames(olm)
colLib <- colors[x]

filter <- apply(olm, 1, function(x) length(x[which(x > 10)]) > 5)
filtered <- as.matrix(olm[filter, ])

negCon <- intersect(negControls[, 2], rownames(filtered))
OLMup <- intersect(positive[positive[, 5] == "UP", 1], rownames(filtered))
OLMdown <- intersect(positive[positive[, 5] == "DOWN", 1], rownames(filtered))
```

3.1 Normalization

As for the FC experiment, we consider UQ and RUVs normalizations.

3.1.1 UQ normalization

```
uqOLM <- betweenLaneNormalization(filtered, which = "upper")

plotRLE(uqOLM, col = colLib, outline = FALSE, las = 3, ylim = c(-0.2, 0.2), ylab = "Relative Log Expressio
    cex.axis = 1, cex.lab = 1)
```

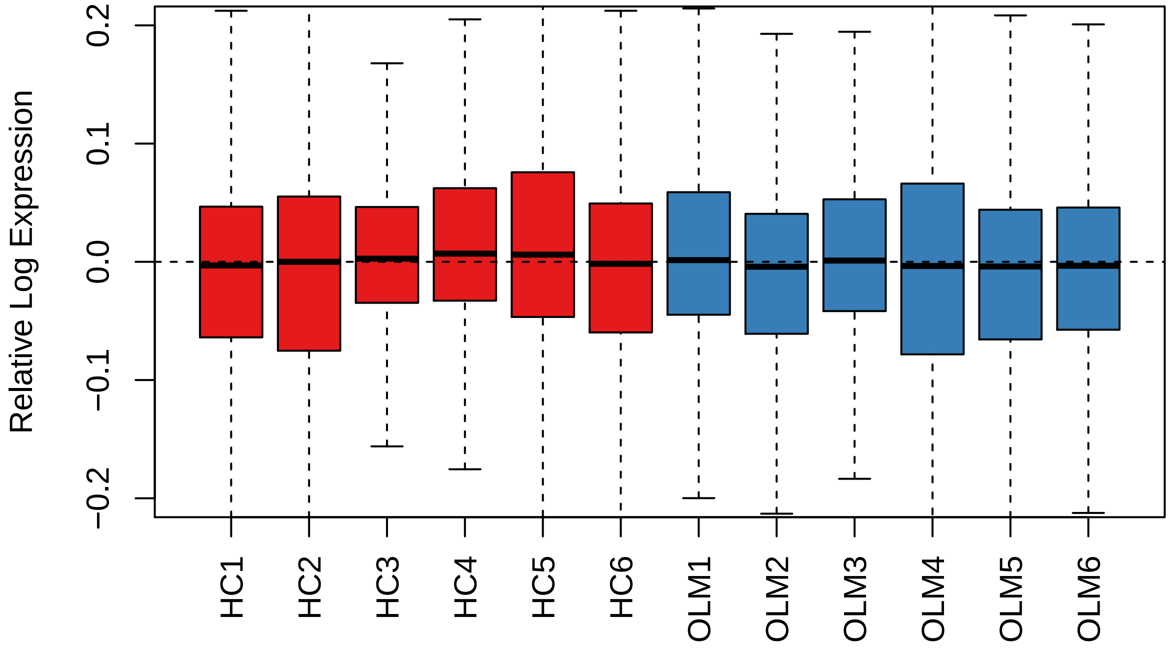


```
plotPCA(uqOLM, col = colLib, cex = 1, cex.axis = 1, cex.lab = 1, xlim = c(-0.7, 0.7),
    ylim = c(-0.7, 0.7))
```

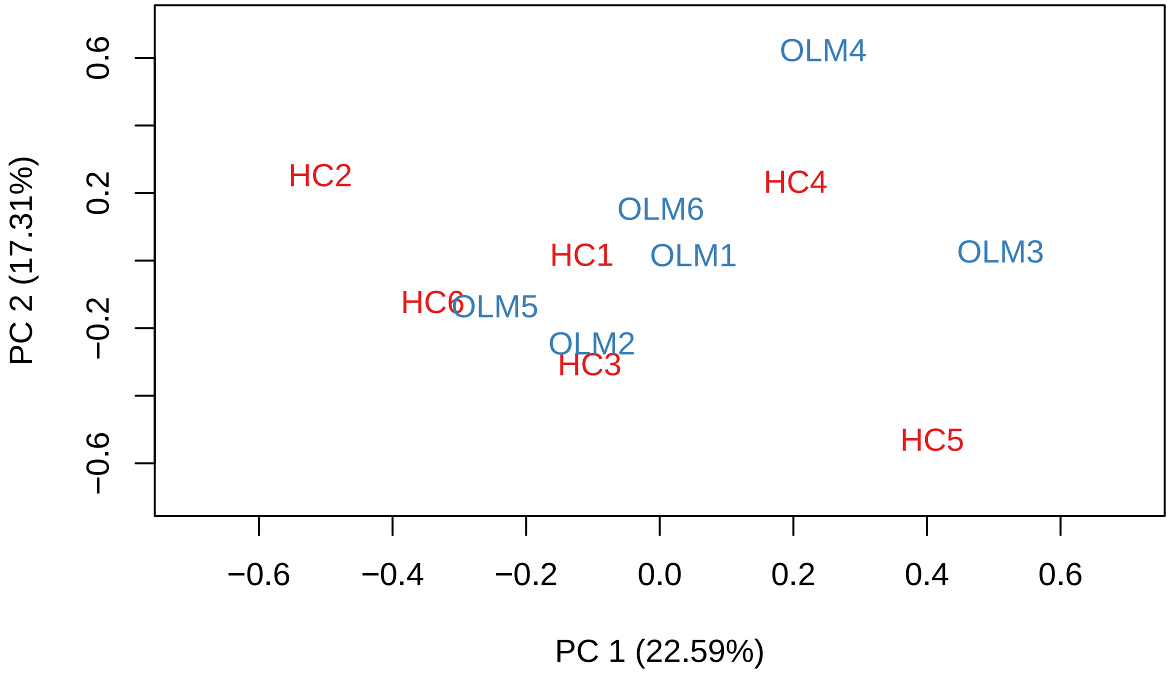

Although the RLE plot looks good, the samples do not cluster by treatment.

3.1.2 RUV normalization

```
groups <- matrix(data = c(1:6, 7:12), nrow = 2, byrow = TRUE)

sOLM <- RUVs(uqOLM, negCon, k = 4, groups)

plotRLE(sOLM$normalizedCounts, col = colLib, outline = FALSE, las = 3, ylim = c(-0.2,
    0.2), ylab = "Relative Log Expression", cex.axis = 1, cex.lab = 1)
```

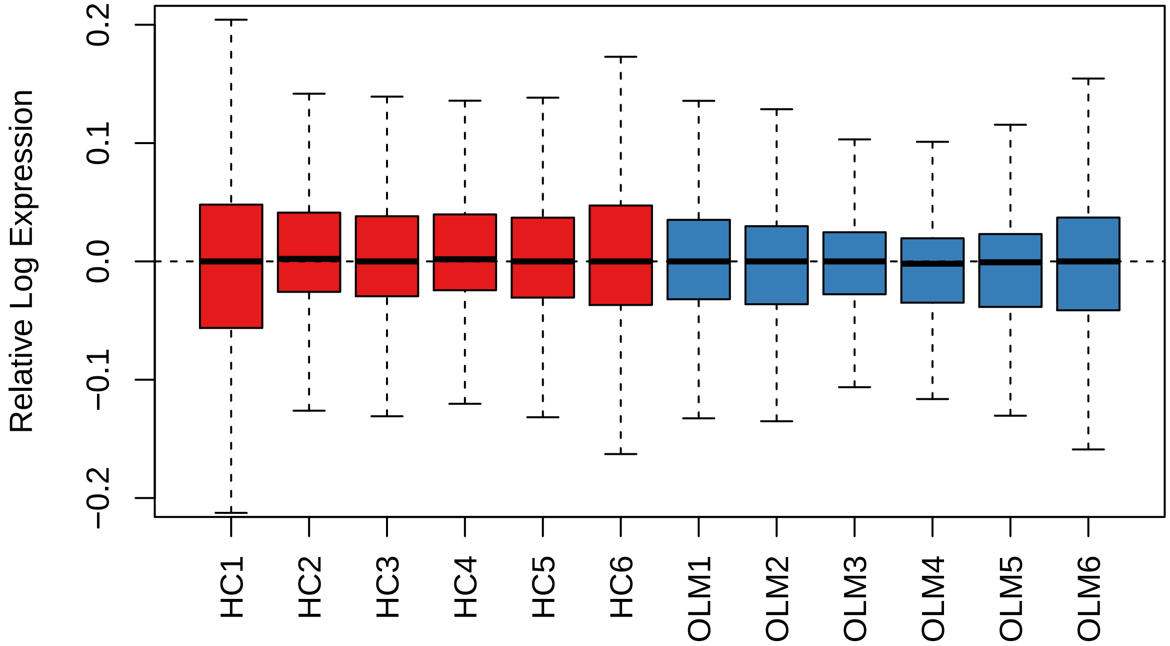

```
plotPCA(sOLM$normalizedCounts, col = colLib, cex = 1, cex.axis = 1, cex.lab = 1,
    xlim = c(-0.7, 0.7), ylim = c(-0.7, 0.7))
```

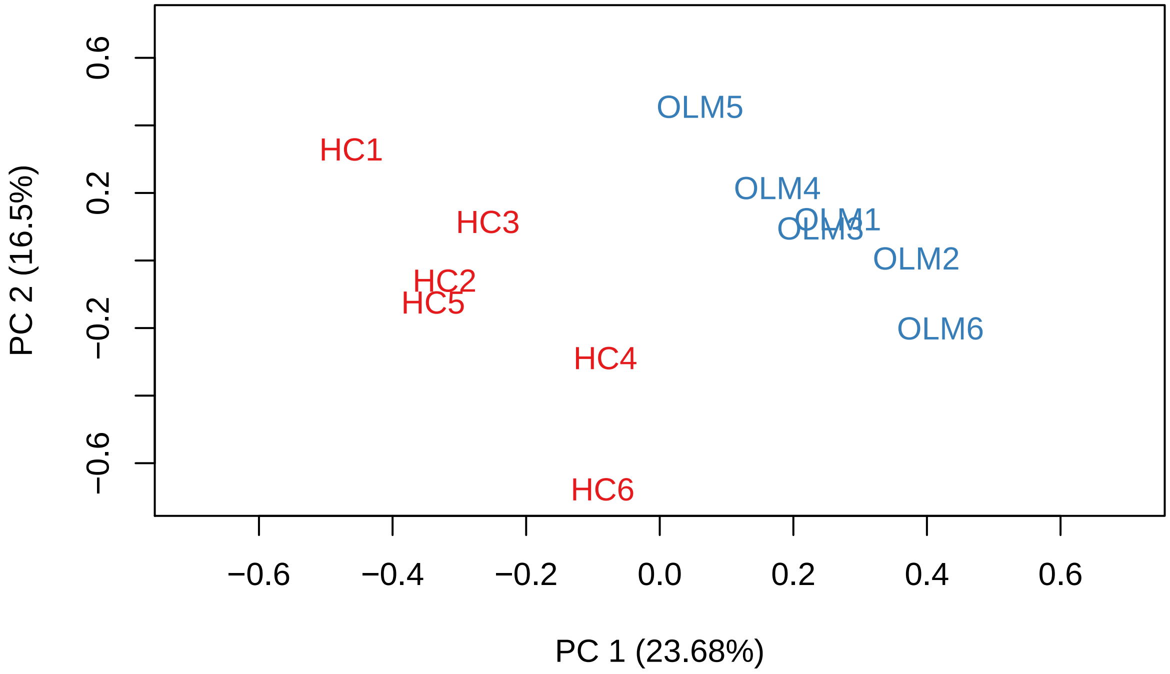

The RLE plot is still looking good and the samples now cluster by treatment in the space of the first two principal components.

3.2 Differential expression

3.2.1 UQ normalization

```
design <- model.matrix(~x)
y <- DGEList(counts = filtered, group = x)
y <- calcNormFactors(y, method = "upperquartile")

y <- estimateGLMCommonDisp(y, design, verbose = TRUE)
```

```
## Disp = 0.01073 , BCV = 0.1036
```

```
y <- estimateGLMTagwiseDisp(y, design)
fit <- glmFit(y, design)

lrt <- glmLRT(fit, coef = 2)
topUQOLM <- topTags(lrt, n = Inf)$table

hist(topUQOLM$PValue, main = "", xlab = "p-value", breaks = 100, ylim = c(0, 600))
```

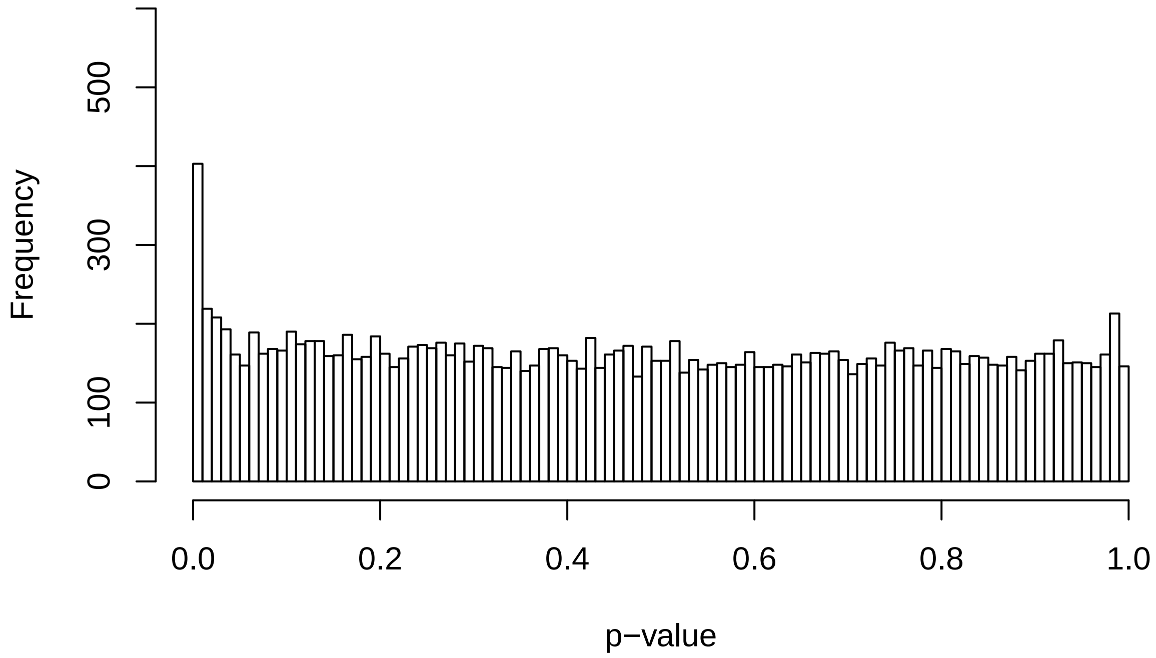


```
plot(topUQOLM[, 1], -log10(topUQOLM$PValue), pch = 20, col = "gray", cex = 0.5, ylab = "-log10(p-value)",
    xlab = "log2(OLM/HC)", ylim = c(0, 80), xlim = c(-3, 3), cex.lab = 1, cex.axis = 1)
de <- rownames(topUQOLM[topUQOLM$FDR <= 0.01, ])
points(topUQOLM[de, 1], -log10(topUQOLM[de, "PValue"]), pch = 20, col = colors[2],
    cex = 1)
points(topUQOLM[OLMup, 1], -log10(topUQOLM[OLMup, "PValue"]), pch = 1, col = colors[1],
    cex = 1, lwd = 2)
points(topUQOLM[OLMdown, 1], -log10(topUQOLM[OLMdown, "PValue"]), pch = 1, col = colors[1],
    cex = 1, lwd = 2)
points(topUQOLM[negCon, 1], -log10(topUQOLM[negCon, "PValue"]), pch = 1, col = colors[3],
    cex = 1, lwd = 2)
```

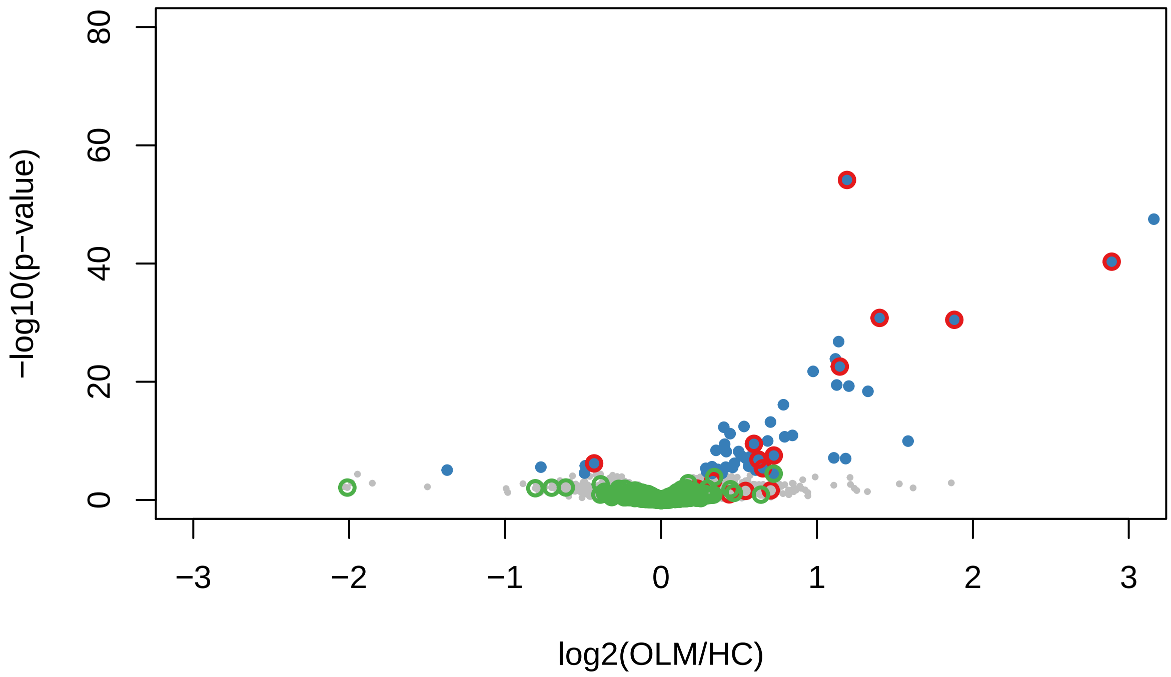

3.2.2 RUV normalization

```
design <- model.matrix(~x + sOLM$W)

y <- DGEList(counts = filtered, group = x)
y <- calcNormFactors(y, method = "upperquartile")

y <- estimateGLMCommonDisp(y, design)
y <- estimateGLMTagwiseDisp(y, design)

fit <- glmFit(y, design)

lrt <- glmLRT(fit, coef = 2)
topSOLM <- topTags(lrt, n = Inf)$table

hist(topSOLM$PValue, main = "", xlab = "p-value", breaks = 100, ylim = c(0, 600))
```

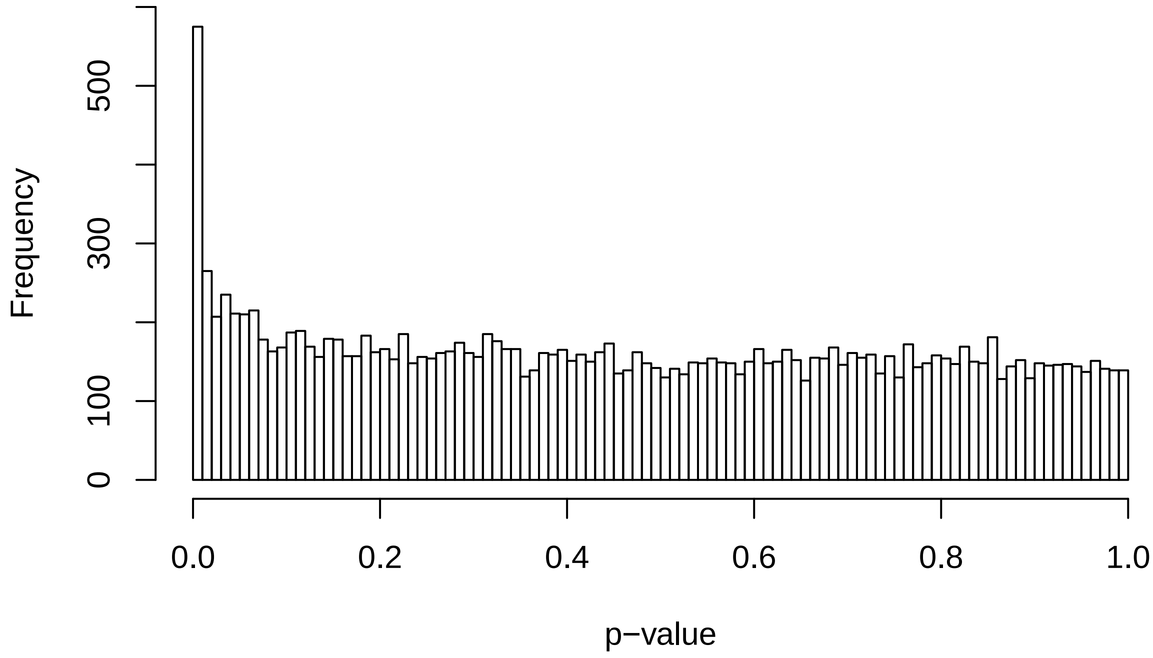


```
plot(topSOLM[, 1], -log10(topSOLM$PValue), pch = 20, col = "gray", cex = 0.5, ylab = "-log10(p-value)",
    xlab = "log2(OLM/HC)", ylim = c(0, 80), xlim = c(-3, 3), cex.lab = 1, cex.axis = 1)
de <- rownames(topSOLM[topSOLM$FDR <= 0.01, ])
points(topSOLM[de, 1], -log10(topSOLM[de, "PValue"]), pch = 20, col = colors[2],
    cex = 1)
points(topSOLM[OLMup, 1], -log10(topSOLM[OLMup, "PValue"]), pch = 1, col = colors[1],
    cex = 1, lwd = 2)
points(topSOLM[OLMdown, 1], -log10(topSOLM[OLMdown, "PValue"]), pch = 1, col = colors[1],
    cex = 1, lwd = 2)
points(topSOLM[negCon, 1], -log10(topSOLM[negCon, "PValue"]), pch = 1, col = colors[3],
    cex = 1, lwd = 2)
```

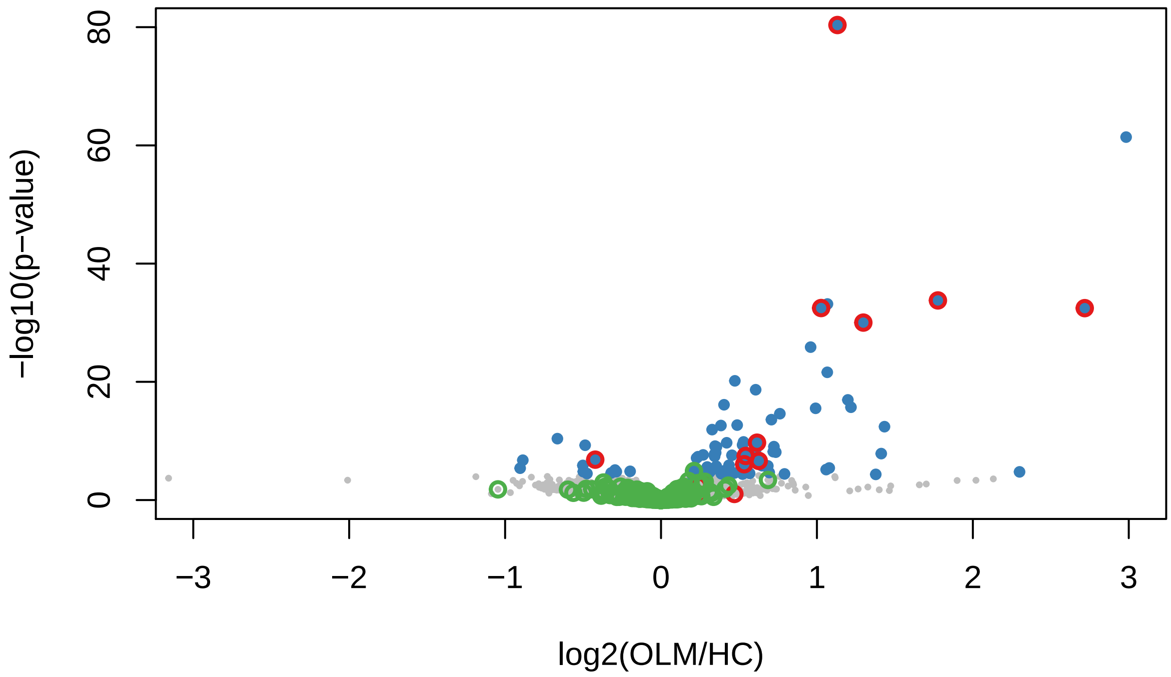

The histograms of the *p*-values look good for both normalizations. The volcano plots show that RUV leads to smaller *p*-values for the positive controls.

4 Combined Analysis

In this section, we consider a combined analysis of the FC and OLM experiments.

```
counts <- cbind(olm, fc[, !grepl("RT", colnames(fc))])
batch <- as.factor(c(rep("lab1", 12), rep("lab2", 10)))
x <- as.factor(c(rep(c("CC", "OLM"), each = 6), rep(c("CC", "FC"), each = 5)))
names(x) <- names(batch) <- colnames(counts)
colors <- brewer.pal(9, "Set1")
colLib <- colors[x]
colBatch <- colors[4:5][batch]

filter <- apply(counts, 1, function(x) length(x[which(x > 10)]) > 5)
filtered <- as.matrix(counts[filter, ])
negCon <- intersect(negControls[, 2], rownames(filtered))
```

4.1 Normalization

4.1.1 UQ normalization

```
uqCombined <- betweenLaneNormalization(filtered, which = "upper")

plotRLE(uqCombined, col = colLib, outline = FALSE, las = 3, ylim = c(-0.2, 0.2),
    ylab = "Relative Log Expression", cex.axis = 1, cex.lab = 1)
```

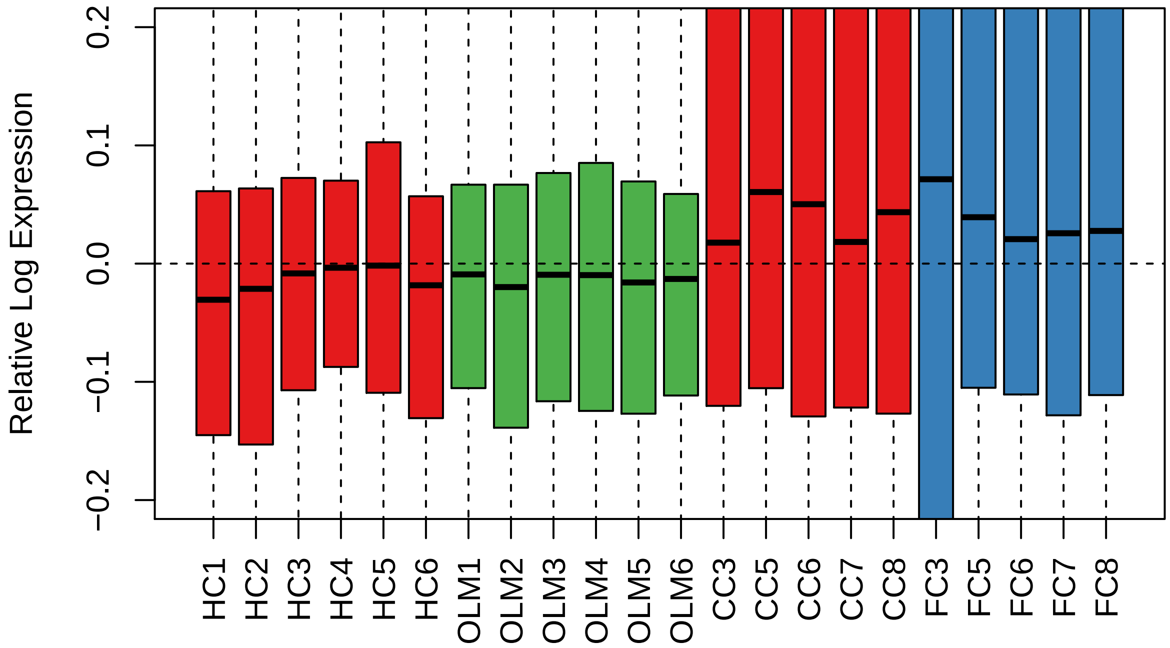


```
plotPCA(uqCombined, col = colLib, cex = 1, cex.axis = 1, cex.lab = 1, xlim = c(-0.4,
    0.6), ylim = c(-0.4, 0.5))
```

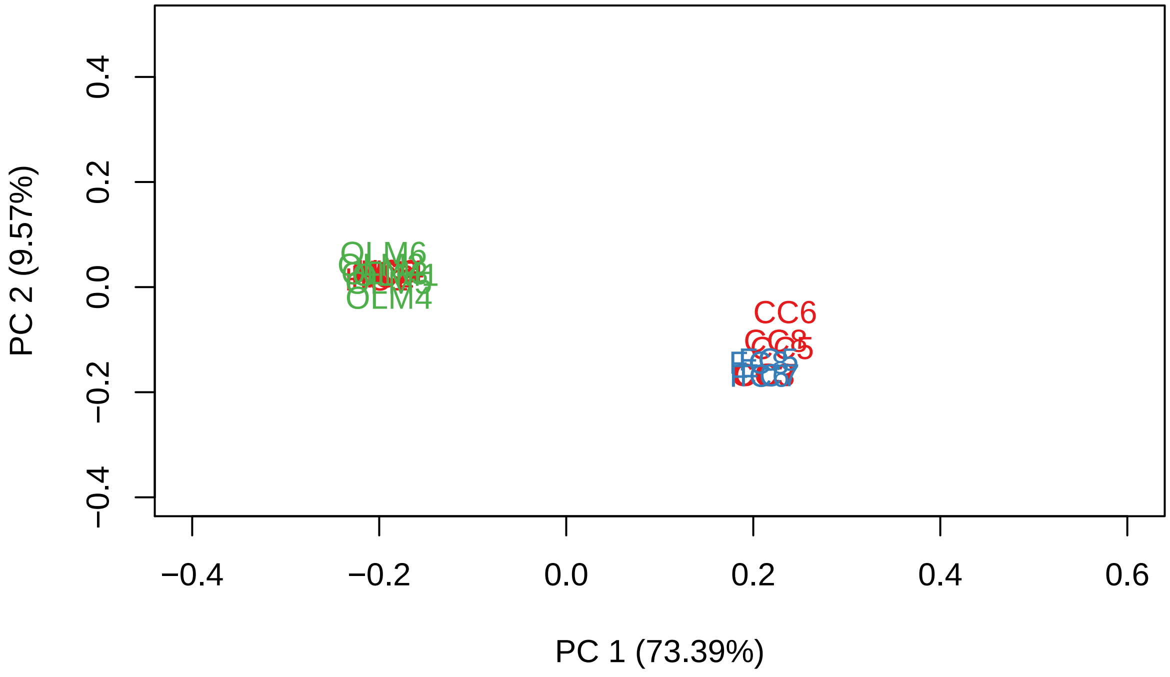

UQ normalization is not able to normalize for the lab effect. Both the RLE plot and the PCA plot show a strong batch effect due to the different labs in which the experiments were carried out. In particular, the samples cluster by lab rather than by treatment.

4.1.2 RUV normalization

Again, we consider the biological replicates to define the replicate groups for RUVs. In particular, we consider as one group the controls from both labs. This ensures that we can remove the lab effect.

```r
groups <- matrix(data = c(1:6, 13:17, 7:12, rep(-1, 5), 18:22, rep(-1, 6)), nrow = 3,
    byrow = TRUE)

sCombined <- RUVs(uqCombined, negCon, k = 6, groups)

plotRLE(sCombined$normalizedCounts, col = colLib, outline = FALSE, las = 3, ylim = c(-0.2,
    0.2), ylab = "Relative Log Expression", cex.axis = 1, cex.lab = 1)
```

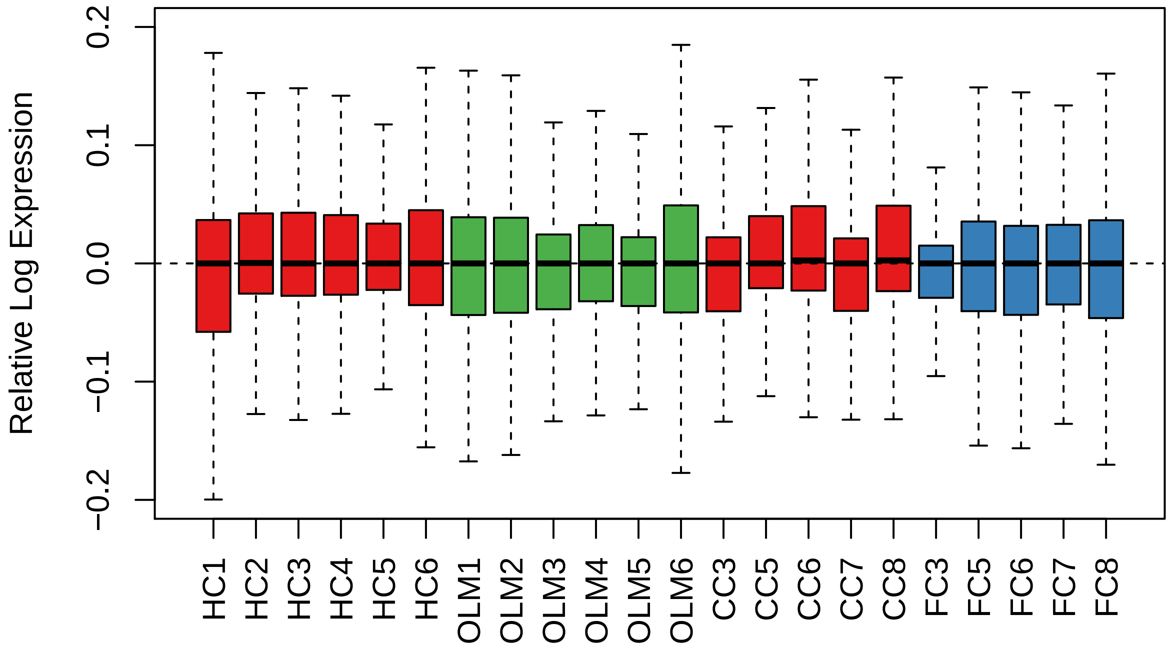


```r
plotPCA(sCombined$normalizedCounts, col = colLib, cex = 1, cex.axis = 1, cex.lab = 1,
    xlim = c(-0.4, 0.6), ylim = c(-0.4, 0.5))
```

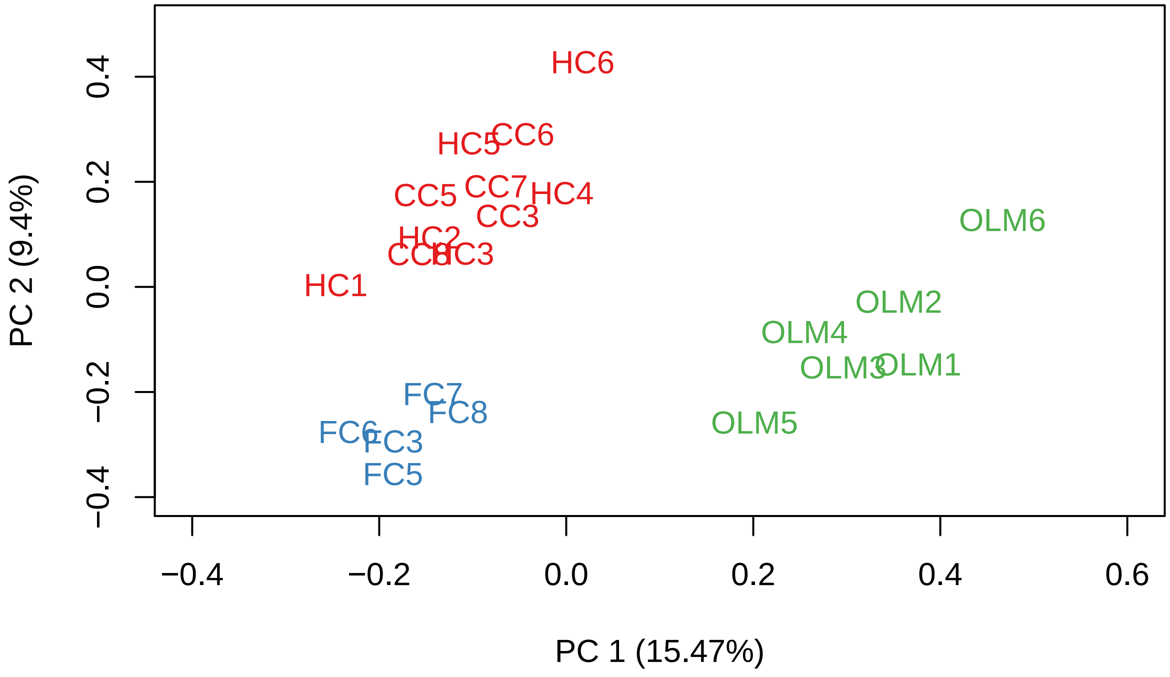

The samples now cluster by treatment. In particular, note how the controls from the two labs cluster together.

4.2 Differential expression

Here, we consider the "average effect" of the OLM and FC tasks compared to the controls as differential expression. Note that the combined analysis allows us to consider many interesting comparisons (not shown here and not run in the code below, for ease of reading). In particular, one can consider the individual effects (FC vs. CC, OLM vs. CC) and the difference between the two tasks (FC vs. OLM).

4.2.1 UQ normalization

```
design <- model.matrix(~x - 1)
y <- DGEList(counts = filtered, group = x)
y <- calcNormFactors(y, method = "upperquartile")

y <- estimateGLMCommonDisp(y, design, verbose = TRUE)
```

```
## Disp = 0.07604 , BCV = 0.2758
```

```
y <- estimateGLMTagwiseDisp(y, design)
fit <- glmFit(y, design)

## (OLM+FC)/2 vs. CC
lrt <- glmLRT(fit, contrast = c(-1, 1/2, 1/2))

## FC vs. CC

## lrt <- glmLRT(fit, contrast=c(-1, 1, 0))

## OLM vs. CC

## lrt <- glmLRT(fit, contrast=c(-1, 0, 1))

## FC vs. OLM

## lrt <- glmLRT(fit, contrast=c(0, 1, -1))

topUQCombined <- topTags(lrt, n = Inf)$table

hist(topUQCombined$PValue, main = "", xlab = "p-value", breaks = 100, ylim = c(0,
    1200))
```

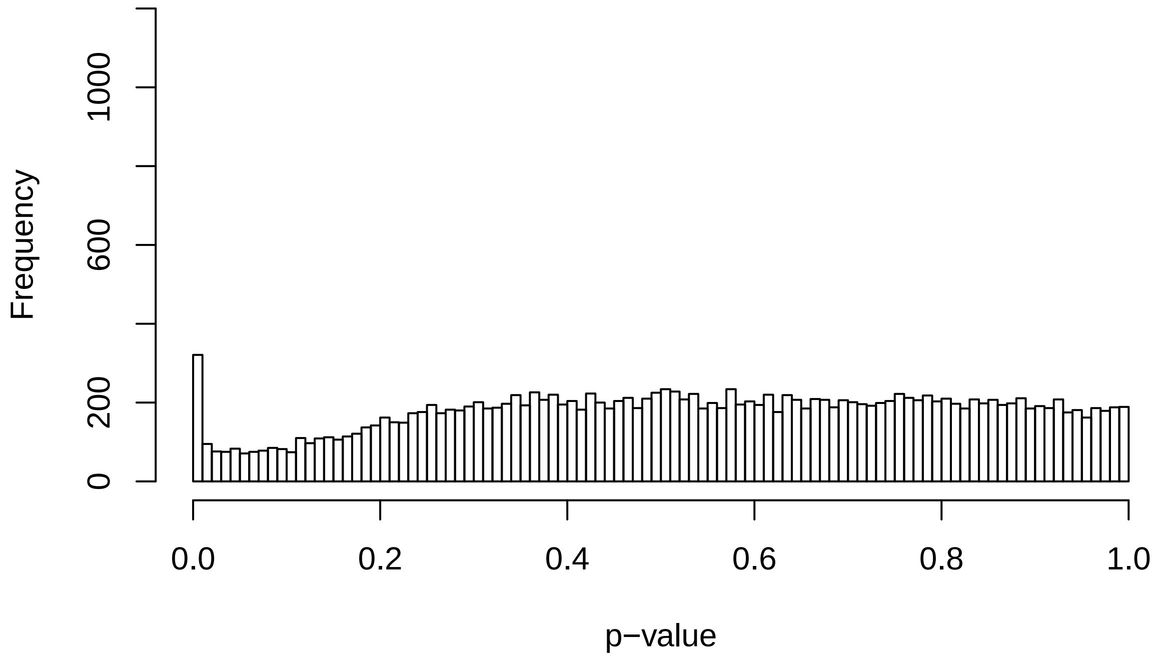


```r
plot(topUQCombined[, 1], -log10(topUQCombined$PValue), pch = 20, col = "gray", cex = 0.5,
    ylab = "-log10(p-value)", xlab = "log2((OLM+FC)/2/CC)", ylim = c(0, 80), xlim = c(-3,
        3), cex.lab = 1, cex.axis = 1)
de <- rownames(topUQCombined[topUQCombined$FDR <= 0.01, ])
points(topUQCombined[de, 1], -log10(topUQCombined[de, "PValue"]), pch = 20, col = colors[2],
    cex = 1)
points(topUQCombined[FCup, 1], -log10(topUQCombined[FCup, "PValue"]), pch = 1, col = colors[1],
    cex = 1, lwd = 2)
points(topUQCombined[FCdown, 1], -log10(topUQCombined[FCdown, "PValue"]), pch = 1,
    col = colors[1], cex = 1, lwd = 2)
points(topUQCombined[OLMup, 1], -log10(topUQCombined[OLMup, "PValue"]), pch = 1,
    col = colors[1], cex = 1, lwd = 2)
points(topUQCombined[OLMdown, 1], -log10(topUQCombined[OLMdown, "PValue"]), pch = 1,
    col = colors[1], cex = 1, lwd = 2)
points(topUQCombined[negCon, 1], -log10(topUQCombined[negCon, "PValue"]), pch = 1,
    col = colors[3], cex = 1, lwd = 2)
```

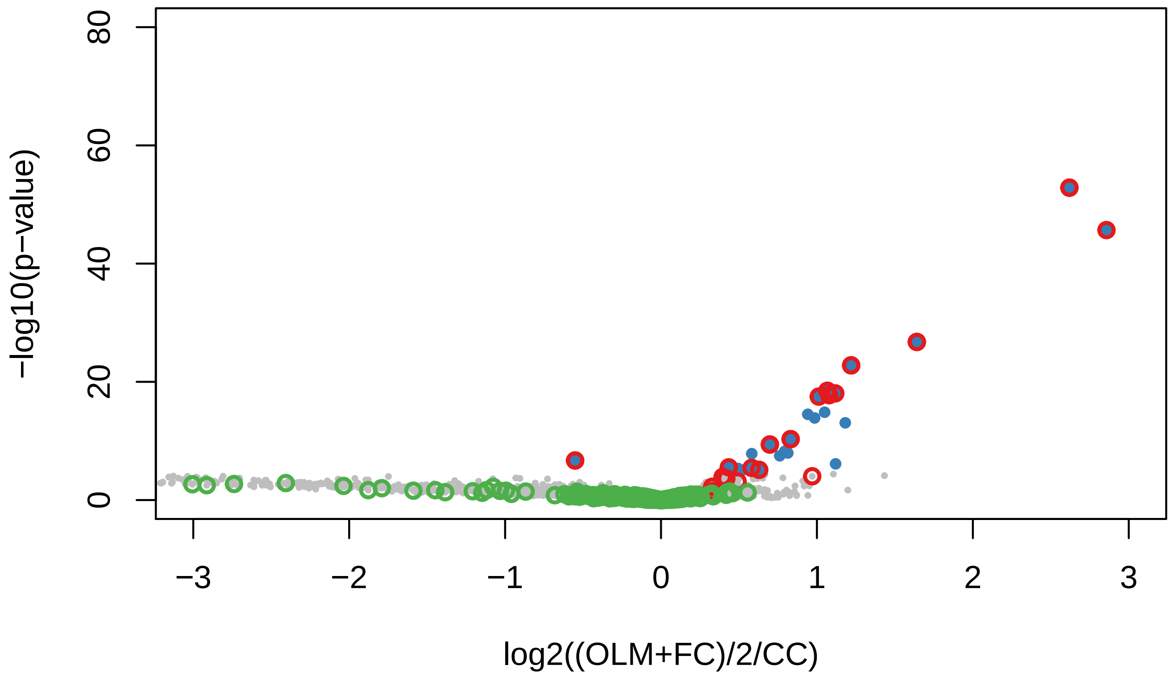

EdgeR's results with UQ normalization are not satisfactory: in particular, some of the positive controls are not detected as DE and some of the negative controls show a strong negative log-fold-change.

4.2.2 RUV normalization

```
design <- model.matrix(~x + sCombined$W - 1)

y <- DGEList(counts = filtered, group = x)
y <- calcNormFactors(y, method = "upperquartile")

y <- estimateGLMCommonDisp(y, design)
y <- estimateGLMTagwiseDisp(y, design)

fit <- glmFit(y, design)

## (OLM+FC)/2 vs. CC
lrt <- glmLRT(fit, contrast = c(-1, 1/2, 1/2, rep(0, 6)))

## FC vs. CC

## lrt <- glmLRT(fit, contrast=c(-1, 1, 0, rep(0, 6)))

## OLM vs. CC

## lrt <- glmLRT(fit, contrast=c(-1, 0, 1, rep(0, 6)))

## FC vs. OLM

## lrt <- glmLRT(fit, contrast=c(0, 1, -1, rep(0, 6)))

topSCombined <- topTags(lrt, n = Inf)$table
```

```
hist(topSCombined$PValue, main = "", xlab = "p-value", breaks = 100, ylim = c(0,
    1200))
```

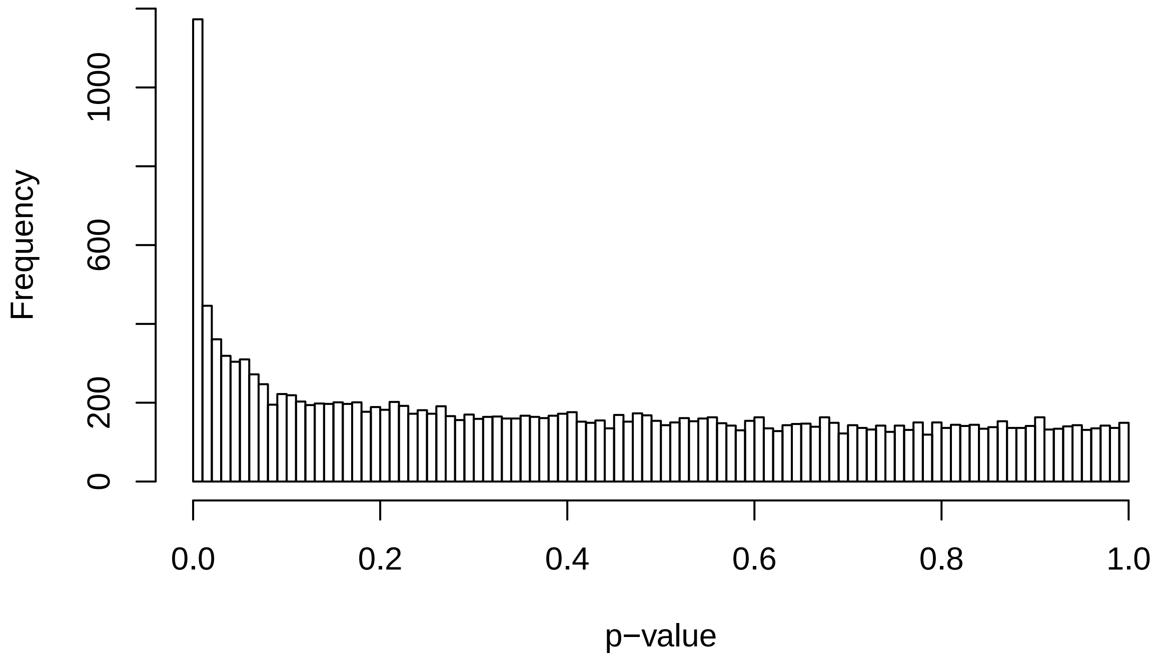


```
plot(topSCombined[, 1], -log10(topSCombined$PValue), pch = 20, col = "gray", cex = 0.5,
    ylab = "-log10(p-value)", xlab = "log2((OLM+FC)/2/CC)", ylim = c(0, 80), xlim = c(-3,
        3), cex.lab = 1, cex.axis = 1)
de <- rownames(topSCombined[topSCombined$FDR <= 0.01, ])
points(topSCombined[de, 1], -log10(topSCombined[de, "PValue"]), pch = 20, col = colors[2],
    cex = 1)
points(topSCombined[FCup, 1], -log10(topSCombined[FCup, "PValue"]), pch = 1, col = colors[1],
    cex = 1, lwd = 2)
points(topSCombined[FCdown, 1], -log10(topSCombined[FCdown, "PValue"]), pch = 1,
    col = colors[1], cex = 1, lwd = 2)
points(topSCombined[OLMup, 1], -log10(topSCombined[OLMup, "PValue"]), pch = 1, col = colors[1],
    cex = 1, lwd = 2)
points(topSCombined[OLMdown, 1], -log10(topSCombined[OLMdown, "PValue"]), pch = 1,
    col = colors[1], cex = 1, lwd = 2)
points(topSCombined[negCon, 1], -log10(topSCombined[negCon, "PValue"]), pch = 1,
    col = colors[3], cex = 1, lwd = 2)
```

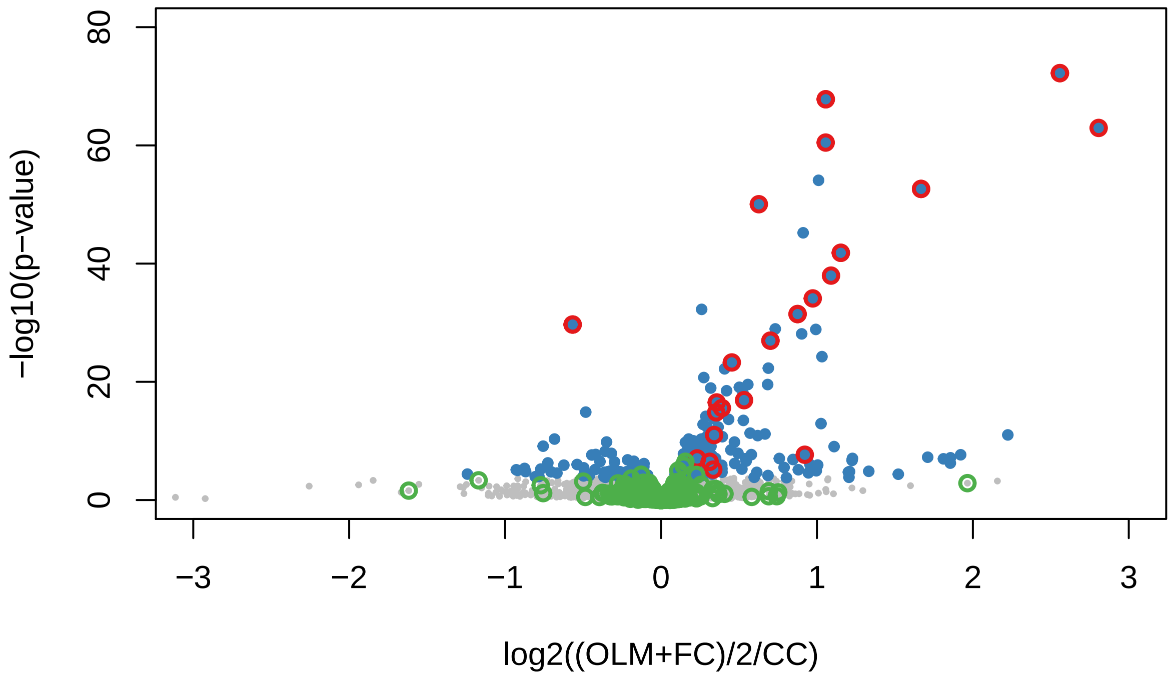

RUV leads to a better distribution of the p-values. In particular, p-values of the positive controls are smaller and those of negative controls are closer to one (zero on the -log scale).

5 Comparison with microarray data

Finally, we look at the concordance between DE lists obtained from RNA-seq (either with UQ or RUV normalization) and microarray.

We consider a subset of genes, including only the genes mapped uniquely by the Affymetrix probe-sets and detected by RNA-seq, and only the samples assessed with both technologies.

```
data <- read.table("Peixoto_Additional_inputtext/Peixoto_FC_array_combined.txt",
    header = TRUE, as.is = TRUE, row.names = 74)
data <- data[, -1]

platform <- rep("seq", ncol(data))
platform[grep("_", colnames(data))] <- "array"
platform <- as.factor(platform)
names(platform) <- colnames(data)

x <- substr(colnames(data), 1, 2)
x[x == "TT"] <- "RT"
x <- as.factor(x)
names(x) <- colnames(data)

## subset
include <- c(paste("CC30", c(3, 5, 6, 7), sep = "_"), paste("FC30", c(3, 5, 6, 7),
    sep = "_"), paste("CC", c(3, 5, 6, 7), sep = ""), paste("FC", c(3, 5, 6, 7),
    sep = ""))
data <- data[, include]
x <- droplevels(x[include])
platform <- droplevels(platform[include])
```

```
### filter
array <- as.matrix(data[, platform == "array"])
seq <- as.matrix(data[, platform == "seq"])
xA <- x[platform == "array"]
xS <- x[platform == "seq"]
filter <- apply(seq, 1, function(x) length(x[x > 5]) > 3)

array <- array[filter, ]
seq <- seq[filter, ]
```

First, we consider a DE analysis of the microarray only, using limma.

```
design <- model.matrix(~xA)
fit <- lmFit(array, design)
fit <- eBayes(fit)
top <- topTable(fit, coef = 2, n = Inf)
deLimma <- rownames(top)[top$adj.P.Val <= 0.1]
```

As for RNA-seq, we consider edgeR, either with UQ or with RUV normalization.

```
design <- model.matrix(~xS)
y <- DGEList(counts = seq, group = xS)
y <- calcNormFactors(y, method = "upperquartile")

y <- estimateGLMCommonDisp(y, design, verbose = TRUE)
```

```
## Disp = 0.05178 , BCV = 0.2276
```

```
y <- estimateGLMTagwiseDisp(y, design)

fit <- glmFit(y, design)
lrt <- glmLRT(fit, coef = 2)
topUQ <- topTags(lrt, n = nrow(seq))$table
deUQ <- rownames(topUQ[topUQ$FDR < 0.1, ])
```

```
negCon <- intersect(negControls[, 2], rownames(seq))
norm <- betweenLaneNormalization(seq, which = "upper")
groups <- matrix(data = c(1:4, 5:8), nrow = 2, byrow = TRUE)
rS <- RUVs(norm, negCon, k = 1, groups)

design <- model.matrix(~xS + rS$W)
y <- DGEList(counts = seq, group = xS)
y <- calcNormFactors(y, method = "upperquartile")

y <- estimateGLMCommonDisp(y, design, verbose = TRUE)
```

```
## Disp = 0.00299 , BCV = 0.0546
```

```
y <- estimateGLMTagwiseDisp(y, design)

fit <- glmFit(y, design)
lrt <- glmLRT(fit, coef = 2)
topRS <- topTags(lrt, n = nrow(seq))$table
deRS <- rownames(topRS[topRS$FDR < 0.1, ])
```

We compare the two normalizations in terms of concordance between the genes identified DE by microarrays and RNA-seq. The concordance is defined, for the i top ranked genes by p-value, as the number of common genes divided by i.

```
limmaP <- top$P.Value
names(limmaP) <- rownames(top)

uqP <- topUQ$PValue
names(uqP) <- rownames(topUQ)

ruvSP <- topRS$PValue
names(ruvSP) <- rownames(topRS)

uq_limma = CATplot(uqP, limmaP, maxrank = 1000, make.plot = F)
ruvS_limma = CATplot(ruvSP, limmaP, maxrank = 1000, make.plot = F)


ul <- uq_limma[1:500, ]
rl <- ruvS_limma[1:500, ]
plot(ul[-(1:20), ], ylim = c(0.15, 0.55), col = colors[1], lwd = 2, type = "l", cex.axis = 1,
    cex.lab = 1)
lines(rl[-(1:20), ], col = colors[2], lwd = 2)
legend("topright", legend = c("UQ vs. limma", "RUVs vs limma"), col = colors, lwd = 2,
    cex = 1)
```

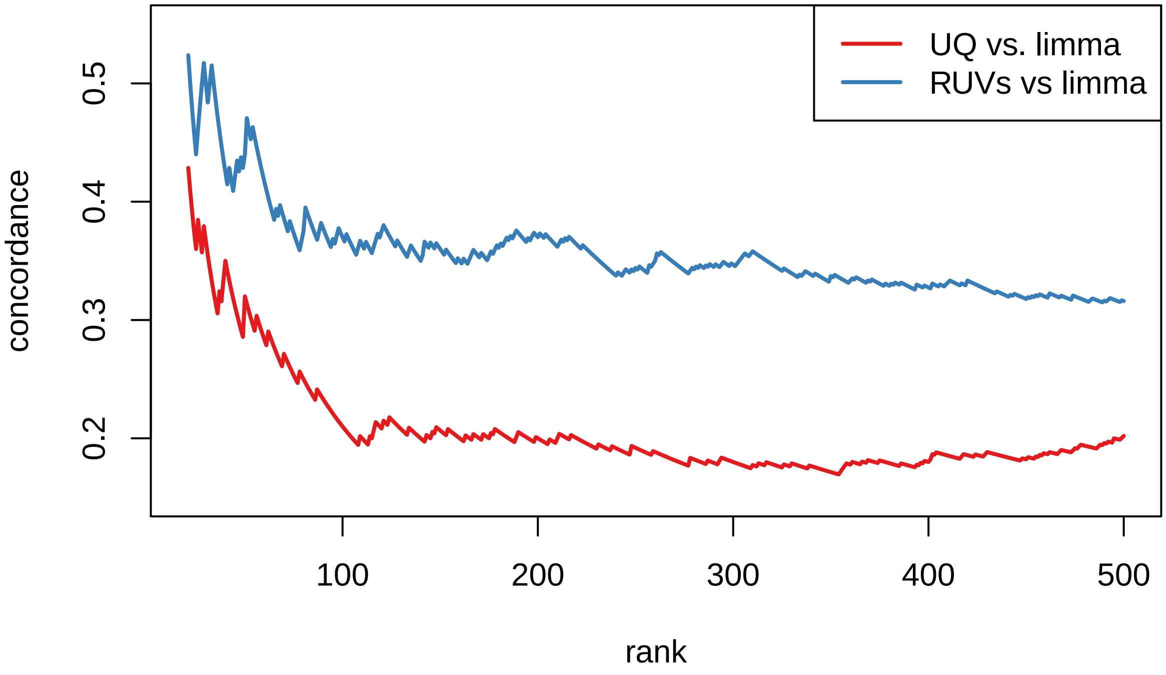

6 Tuning parameters

The two main tuning parameters of RUV are the number of factors of unwanted variation, k, and the set of negative controls genes. In this section, we provide guidelines on how to select these parameters and we show that RUVs is robust to the choice of negative controls.

6.1 Number of factors of unwanted variation (k)

The choice of the parameter k is not easy and is dataset-dependent. Hence, we recommend to perform extensive exploratory data analysis, comparing different values of k.

For this task, we found very useful to compare the RLE and PCA plots of the normalized data, as well as volcano plot and histogram of the p-values.

In what follows, we show an example with the FC data, that highlights how a too low value, as well as a too high value, for k leads to sub-optimal results, compared to the chosen $k = 5$.

6.1.1 $k = 1$

```
filter <- apply(fc, 1, function(x) length(x[which(x > 10)]) > 5)
filtered <- as.matrix(fc)[filter, ]
negCon <- intersect(negControls[, 2], rownames(filtered))

x <- as.factor(rep(c("CC", "FC", "RT"), each = 5))
names(x) <- colnames(fc)
colLib <- colors[x]

uq <- betweenLaneNormalization(filtered, which = "upper")

groups <- matrix(data = c(1:5, 6:10, 11:15), nrow = 3, byrow = TRUE)

s1 <- RUVs(uq, negCon, k = 1, groups)

plotRLE(s1$normalizedCounts, col = colLib, outline = FALSE, las = 3, ylim = c(-0.2,
    0.2), ylab = "Relative Log Expression", cex.axis = 1, cex.lab = 1)
```

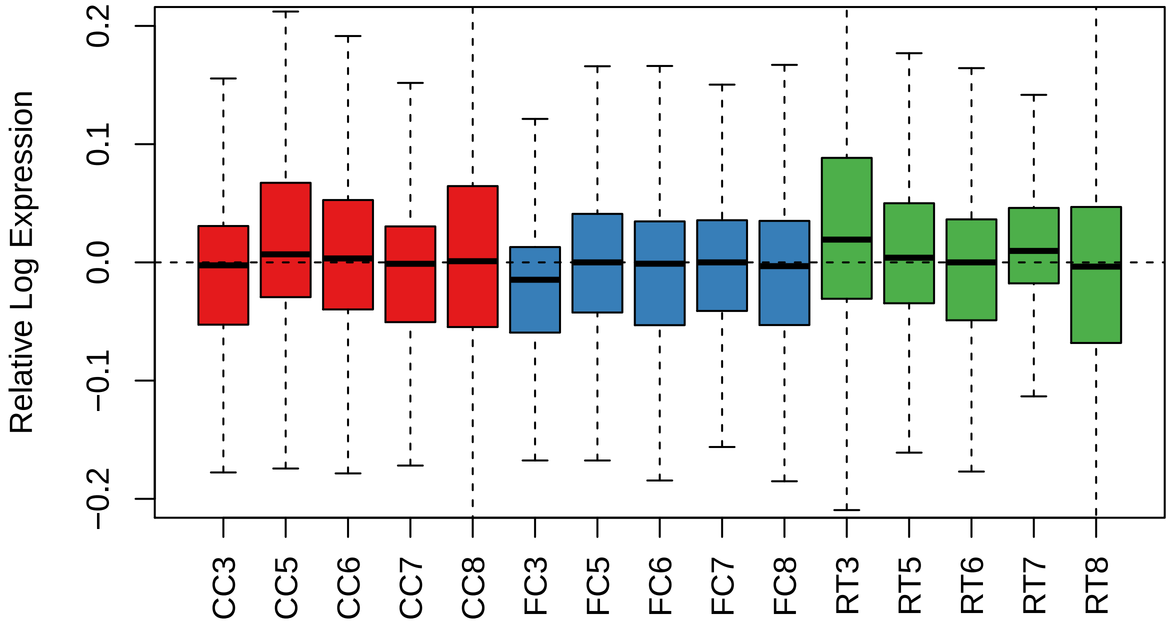


```
plotPCA(s1$normalizedCounts, col = colLib, cex = 1, cex.axis = 1, cex.lab = 1, xlim = c(-0.6,
    0.9), ylim = c(-0.7, 0.6))
```

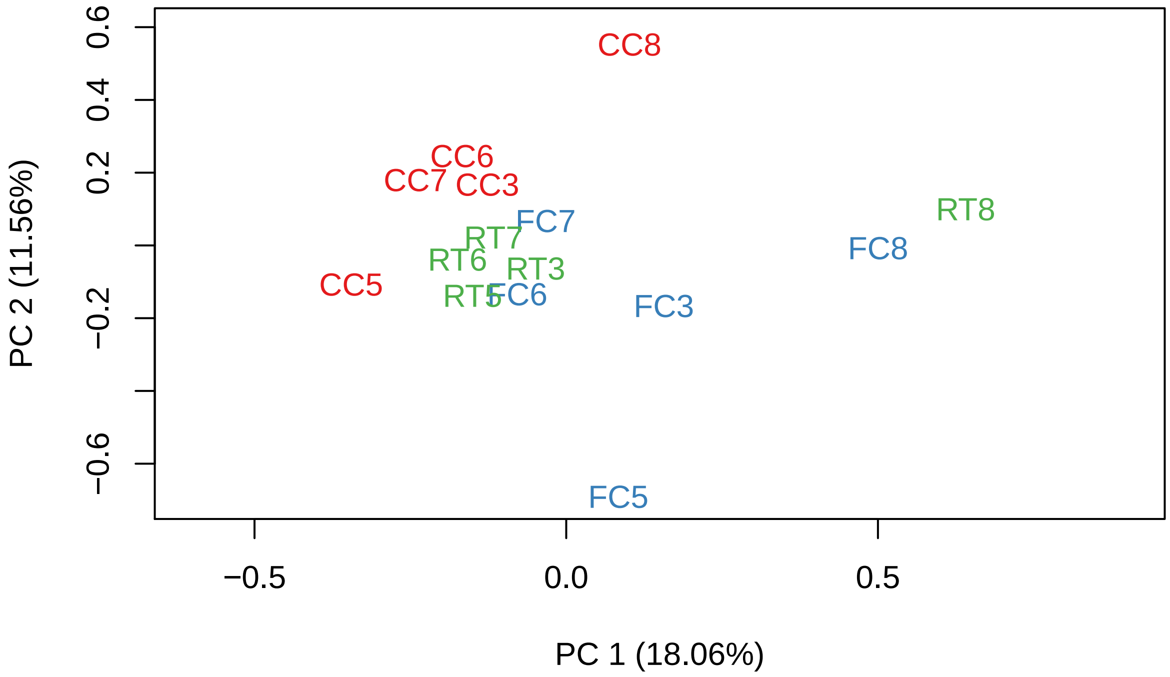

With $k = 1$ the RLE plot shows residual unwanted variation for samples CC8, RT3 and RT8. More importantly, the samples fail to cluster by condition in the space of the first two PCs.

6.1.2 $k = 5$

```
plotRLE(s$normalizedCounts, col = colLib, outline = FALSE, las = 3, ylim = c(-0.2,
    0.2), ylab = "Relative Log Expression", cex.axis = 1, cex.lab = 1)
```

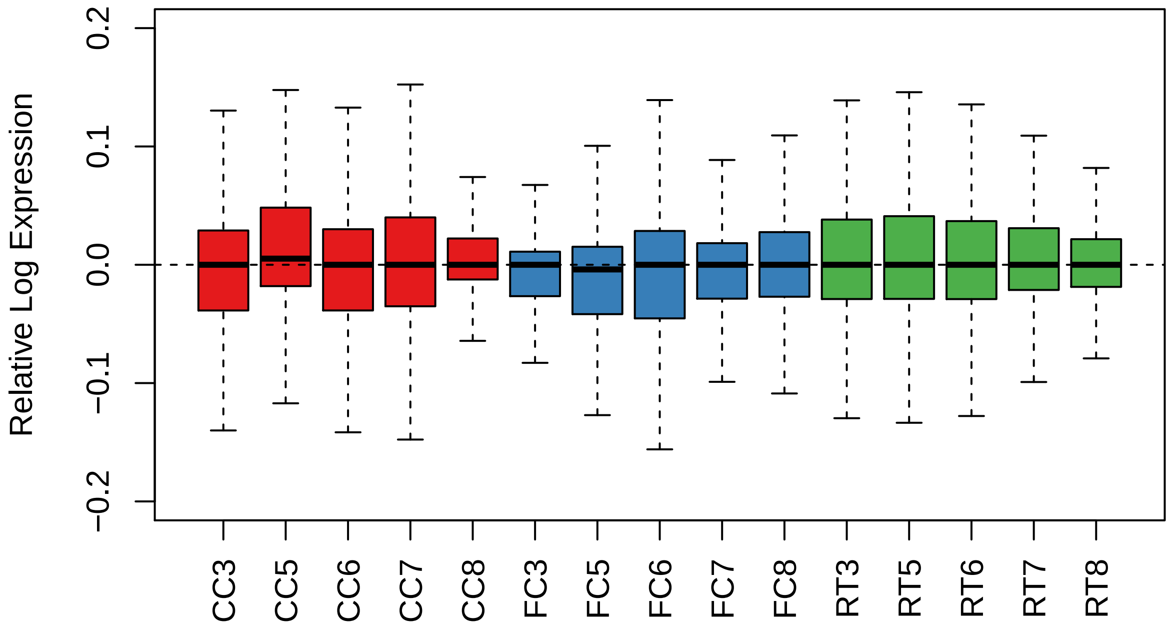


```
plotPCA(s$normalizedCounts, col = colLib, cex = 1, cex.axis = 1, cex.lab = 1, xlim = c(-0.6,
    0.9), ylim = c(-0.7, 0.6))
```

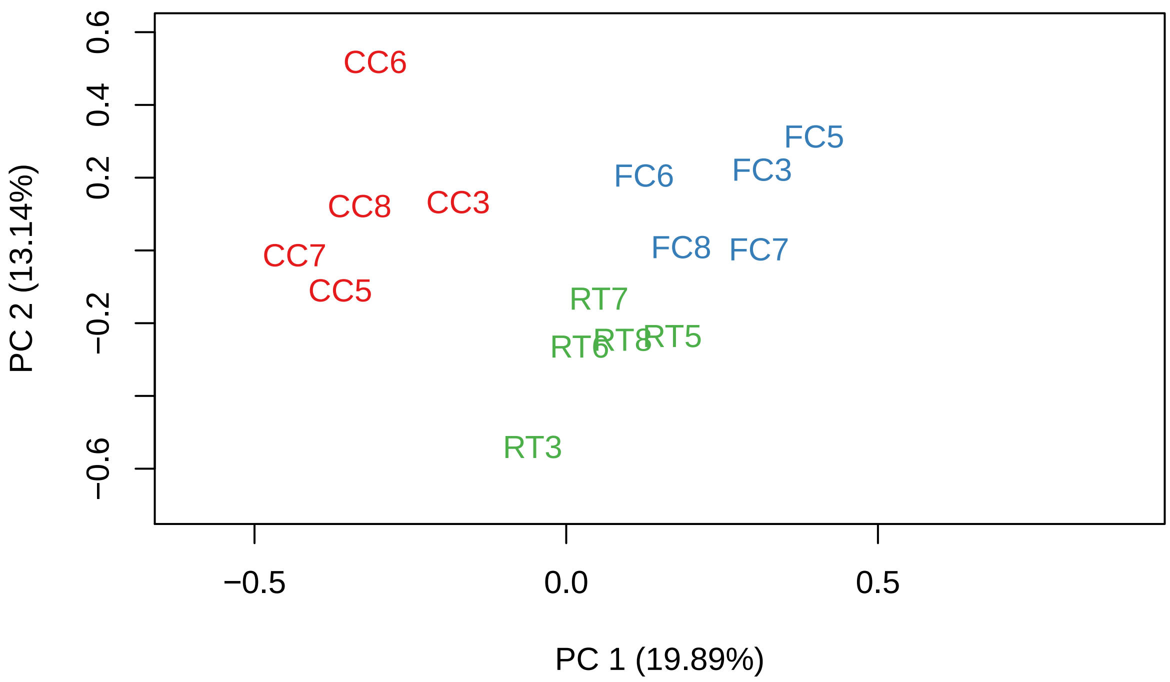

$k = 5$ is a good choice because the samples show similar RLE distributions and cluster by condition.

6.1.3 $k = 10$

```
s10 <- RUVs(uq, negCon, k = 10, groups)

plotRLE(s10$normalizedCounts, col = colLib, outline = FALSE, las = 3, ylim = c(-0.2,
    0.2), ylab = "Relative Log Expression", cex.axis = 1, cex.lab = 1)
```

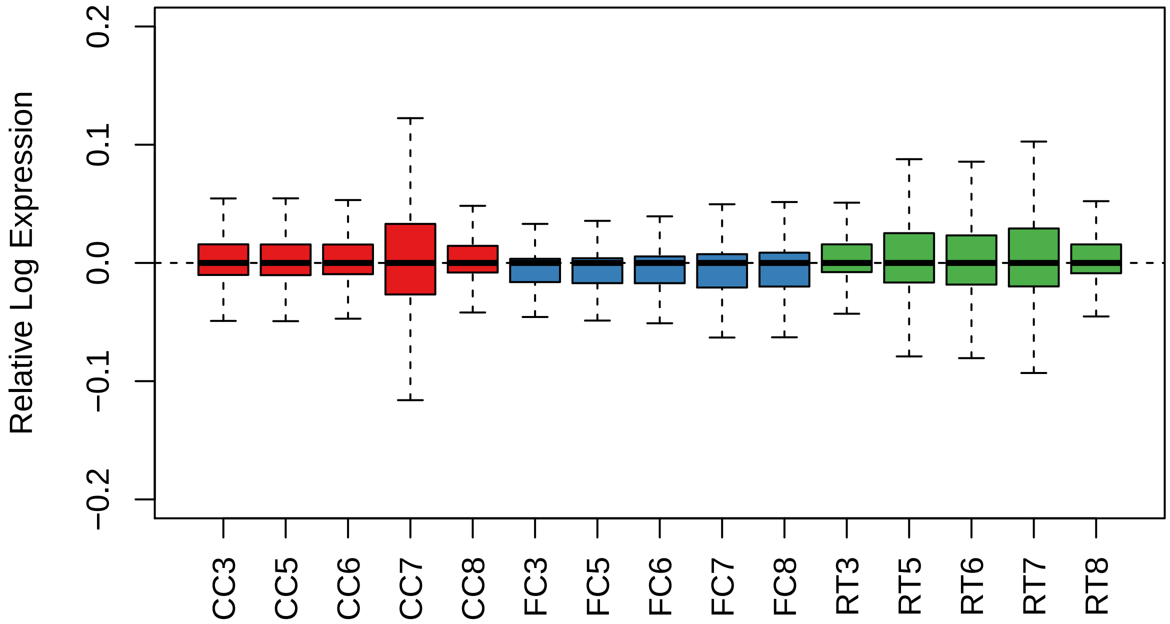


```
plotPCA(s10$normalizedCounts, col = colLib, cex = 1, cex.axis = 1, cex.lab = 1, xlim = c(-0.6,
    0.9), ylim = c(-0.7, 0.6))
```

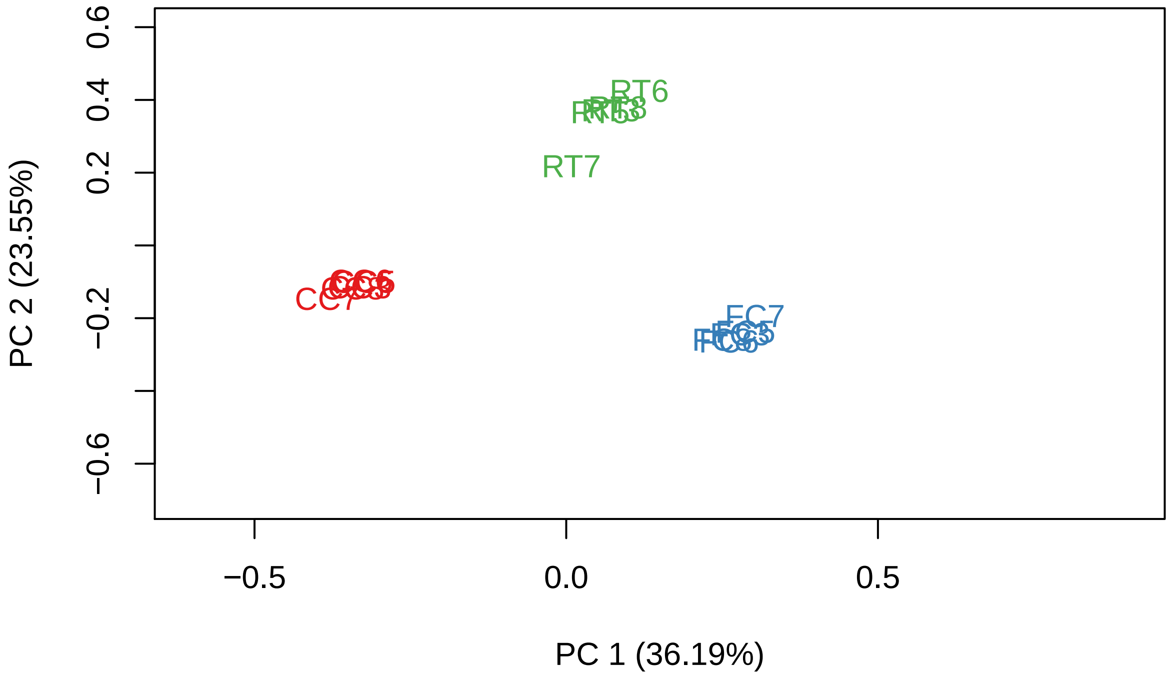

When the value of k is too high, the RLE distributions become uneven again. More importantly, the model over-corrects for unwanted variation and ends up removing (almost) all the biological variability within the conditions. This is shown in the PCA plot, where all the biological replicate samples are collapsed to almost the same coordinates.

6.2 Set of negative control genes

The choice of the set of negative control genes is somewhat dependent on the dataset. When results on similar datasets are available (either because of previous experiments or through publicly available data), it is often a good choice to use a context-specific set of negative controls.

In the absence of such a tailored set, alternative choices are a set of housekeeping genes, a set of synthetic controls (*spike-ins*), or a set of *in silico* empirical controls (Risso et al. 2014).

A good property of RUVs is its robustness to the choice of negative control genes. To highlight this robustness, we show the performance of RUVs when using all the genes as negative controls ($k = 5$).

```
sAll <- RUVs(uq, rownames(uq), k = 5, groups)

plotRLE(sAll$normalizedCounts, col = colLib, outline = FALSE, las = 3, ylim = c(-0.2,
    0.2), ylab = "Relative Log Expression", cex.axis = 1, cex.lab = 1)
```

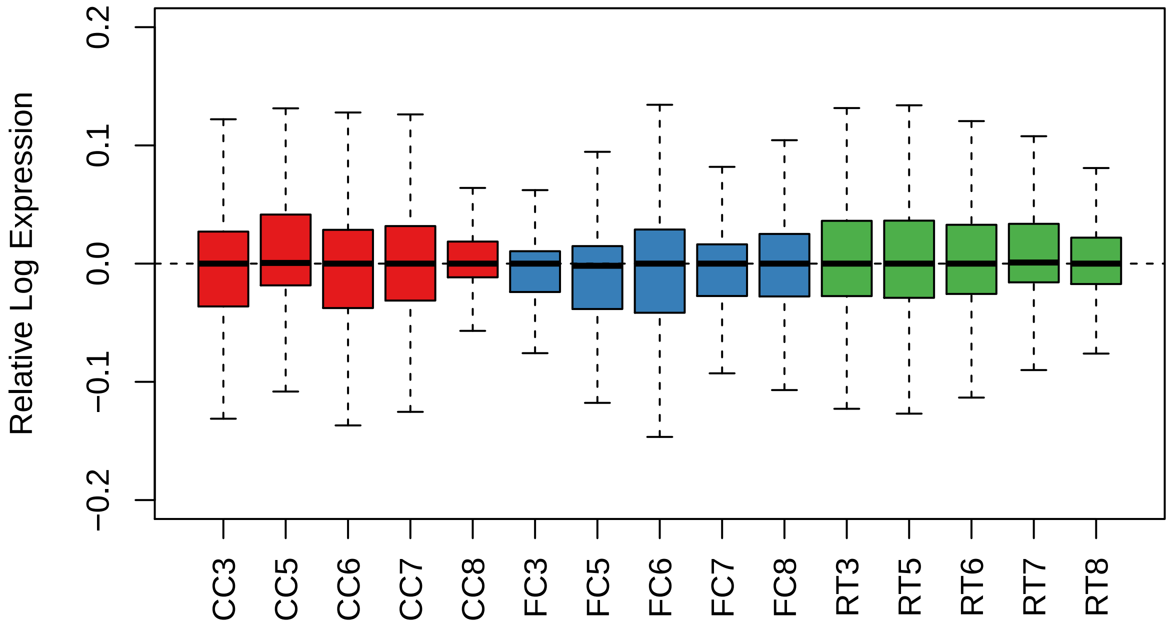


```
plotPCA(sAll$normalizedCounts, col = colLib, cex = 1, cex.axis = 1, cex.lab = 1,
    xlim = c(-0.6, 0.9), ylim = c(-0.7, 0.6))
```

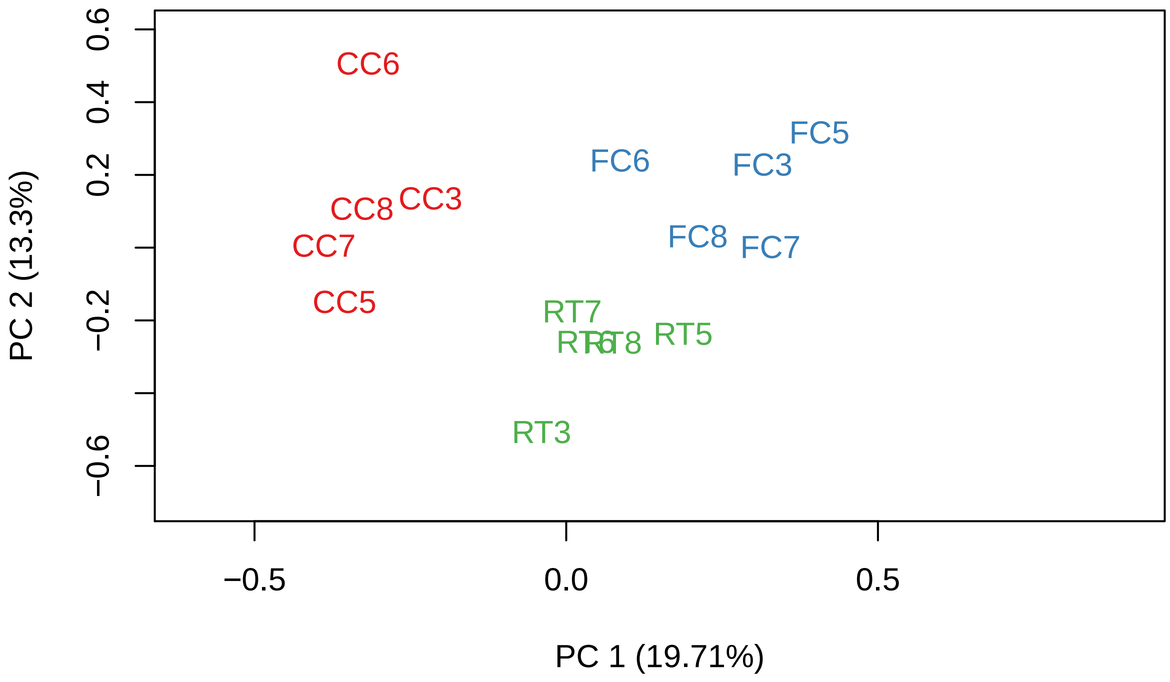

The results are very close to those based on the set of negative controls (see above).

7 Session Info

```
sessionInfo()
```

```
## R version 3.2.0 (2015-04-16)
## Platform: x86_64-apple-darwin13.4.0 (64-bit)
```

```
## Running under: OS X 10.10.3 (Yosemite)
##
## locale:
## [1] en_US.UTF-8/en_US.UTF-8/en_US.UTF-8/C/en_US.UTF-8/en_US.UTF-8
##
## attached base packages:
## [1] stats4    parallel  stats     graphics  grDevices utils
## [7] datasets  methods   base
##
## other attached packages:
##  [1] RColorBrewer_1.1-2      ffpe_1.12.0
##  [3] TTR_0.22-0              xts_0.9-7
##  [5] zoo_1.7-12              RUVSeq_1.2.0
##  [7] EDASeq_2.2.0            ShortRead_1.26.0
##  [9] GenomicAlignments_1.4.1 Rsamtools_1.20.4
## [11] GenomicRanges_1.20.4    GenomeInfoDb_1.4.0
## [13] Biostrings_2.36.1       XVector_0.8.0
## [15] IRanges_2.2.2           S4Vectors_0.6.0
## [17] BiocParallel_1.2.2      Biobase_2.28.0
## [19] BiocGenerics_0.14.0     edgeR_3.10.2
## [21] limma_3.24.5
##
## loaded via a namespace (and not attached):
##  [1] nlme_3.1-120          bitops_1.0-6
##  [3] matrixStats_0.14.0    tools_3.2.0
##  [5] doRNG_1.6             nor1mix_1.2-0
##  [7] affyio_1.36.0         KernSmooth_2.23-14
##  [9] DBI_0.3.1             mgcv_1.8-6
## [11] colorspace_1.2-6      methylumi_2.14.0
## [13] base64_1.1            preprocessCore_1.30.0
## [15] formatR_1.2           pkgmaker_0.22
## [17] rtracklayer_1.28.4    sfsmisc_1.0-27
## [19] genefilter_1.50.0     quadprog_1.5-5
## [21] affy_1.46.0           DESeq_1.20.0
## [23] stringr_1.0.0         digest_0.6.8
## [25] illuminaio_0.10.0     rmarkdown_0.6.1
## [27] siggenes_1.42.0       R.utils_2.1.0
## [29] GEOquery_2.34.0       lumi_2.20.1
## [31] htmltools_0.2.6       RSQLite_1.0.0
## [33] BiocInstaller_1.18.2  hwriter_1.3.2
## [35] mclust_5.0.1          R.oo_1.19.0
## [37] RCurl_1.95-4.6        magrittr_1.5
## [39] futile.logger_1.4.1   Matrix_1.2-1
## [41] Rcpp_0.11.6           R.methodsS3_1.7.0
## [43] stringi_0.4-1         yaml_2.1.13
## [45] nleqslv_2.8           MASS_7.3-40
## [47] zlibbioc_1.14.0       plyr_1.8.2
## [49] bumphunter_1.8.0      grid_3.2.0
## [51] minfi_1.14.0          lattice_0.20-31
## [53] splines_3.2.0         multtest_2.24.0
## [55] GenomicFeatures_1.20.1 annotate_1.46.0
## [57] locfit_1.5-9.1        knitr_1.10.5
## [59] beanplot_1.2          rngtools_1.2.4
## [61] geneplotter_1.46.0    codetools_0.2-11
```

```
## [63] biomaRt_2.24.0         futile.options_1.0.0
## [65] XML_3.98-1.2           evaluate_0.7
## [67] latticeExtra_0.6-26    lambda.r_1.1.7
## [69] foreach_1.4.2          reshape_0.8.5
## [71] aroma.light_2.4.0      xtable_1.7-4
## [73] survival_2.38-1        iterators_1.0.7
## [75] AnnotationDbi_1.30.1   registry_0.2
## [77] BiocStyle_1.6.0
```

References

Anders, Simon, Paul Theodor Pyl, and Wolfgang Huber. 2015. "HTSeq–A Python Framework to Work with High-Throughput Sequencing Data." *Bioinformatics* 31 (2). Oxford Univ Press: 166–69.

Flicek, Paul, M Ridwan Amode, Daniel Barrell, Kathryn Beal, Konstantinos Billis, Simon Brent, Denise Carvalho-Silva, et al. 2014. "Ensembl 2014." *Nucleic Acids Research* 42 (D1). Oxford University Press: D749.

Risso, Davide, John Ngai, Terence P Speed, and Sandrine Dudoit. 2014. "Normalization of RNA-seq Data Using Factor Analysis of Control Genes or Samples." *Nature Biotechnology* 32 (9). Nature Publishing Group: 896–902.

Wu, Thomas D, and Colin K Watanabe. 2005. "GMAP: A Genomic Mapping and Alignment Program for mRNA and EST Sequences." *Bioinformatics* 21 (9). Oxford Univ Press: 1859–75.
