# Supplementary material for: How data analysis affects power, reproducibility and biological insight of RNA-seq studies in complex datasets
Source: Nucleic Acids Res. 2015 Jul 21;43(16):7664–74. doi: 10.1093/nar/gkv736 (PMC4652761; doi:10.1093/nar/gkv736)
Supplement: SUPPLEMENTARY DATA [file supp_gkv736_nar-01134-survey-d-2015-File012.pdf]

Re-analysis of publicly available datasets

Lucia Peixoto, Davide Risso, Shane G. Poplawski, Mathieu, E. Wimmer, Terence P. Speed, Marcelo A. Wood and Ted Abel

We retrieved the pre-processed data of several publicly available studies from GEO (see main text for details). In this Section, we plot the PCA of each dataset using the original normalization.

Starting from the data as normalized by the authors, or applying UQ scaling normalization if the authors provided only raw counts, we apply RUVs using all the genes as negative controls and choosing the value of k that led to the best looking RLE plot. For each dataset, we retained only the genes expressed in at least three replicate samples.

This analysis is intended to show that published normalized datasets often show residual unwanted variation and that RUVs can remove unwanted variation when present and does not compromise the data when scaling normalization is working well. A more careful analysis of each dataset, e.g. by selecting a problem-specific set of negative control genes, could lead to better results.

1 GSE60261

1.1 dicer KO mRNA data, DESeq2 normalization

```
data <- read.table("GEO_datasets/GSE60261.txt", header = TRUE, sep = "\t")
dicerKOmRNA <- as.matrix(data[, 2:ncol(data)])
x <- as.factor(rep(c("KO", "WT"), each = 3))
colLib <- colors[x]

boxplot(log(dicerKOmRNA + 1), las = 2)
```

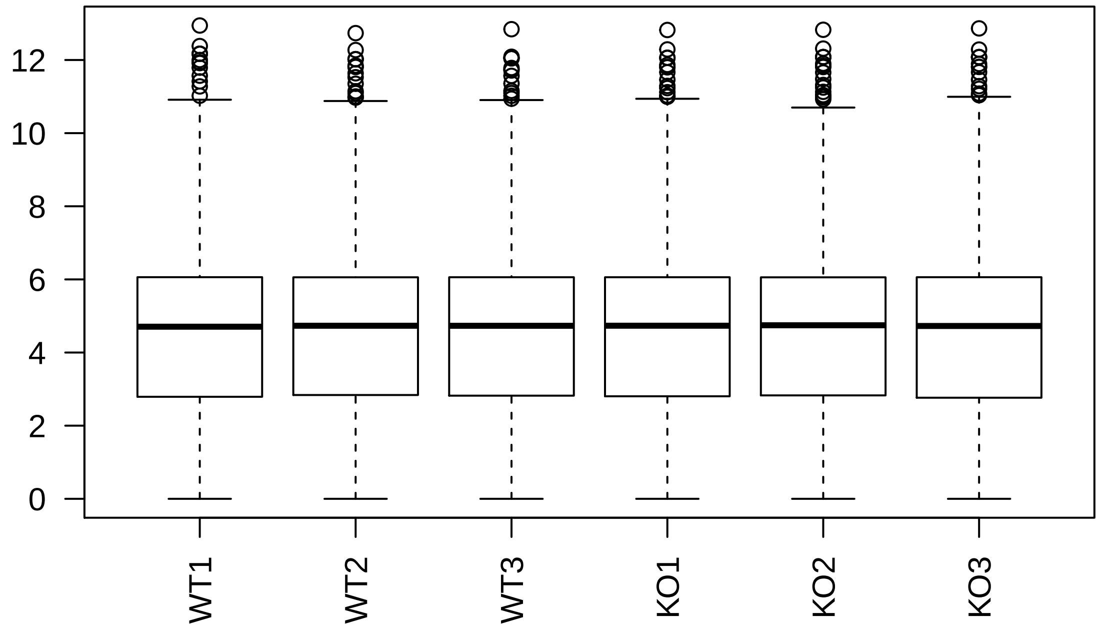


```
filter <- apply(dicerKOmRNA, 1, function(x) length(x[which(x > 0)]) > 3)
table(filter)
```

```
## filter
## FALSE  TRUE
##  8555 68383
```

```
filtered <- dicerKOmRNA[filter, ]

plotRLE(filtered, col = colLib, outline = FALSE, ylim = c(-0.4, 0.4), las = 2)
```

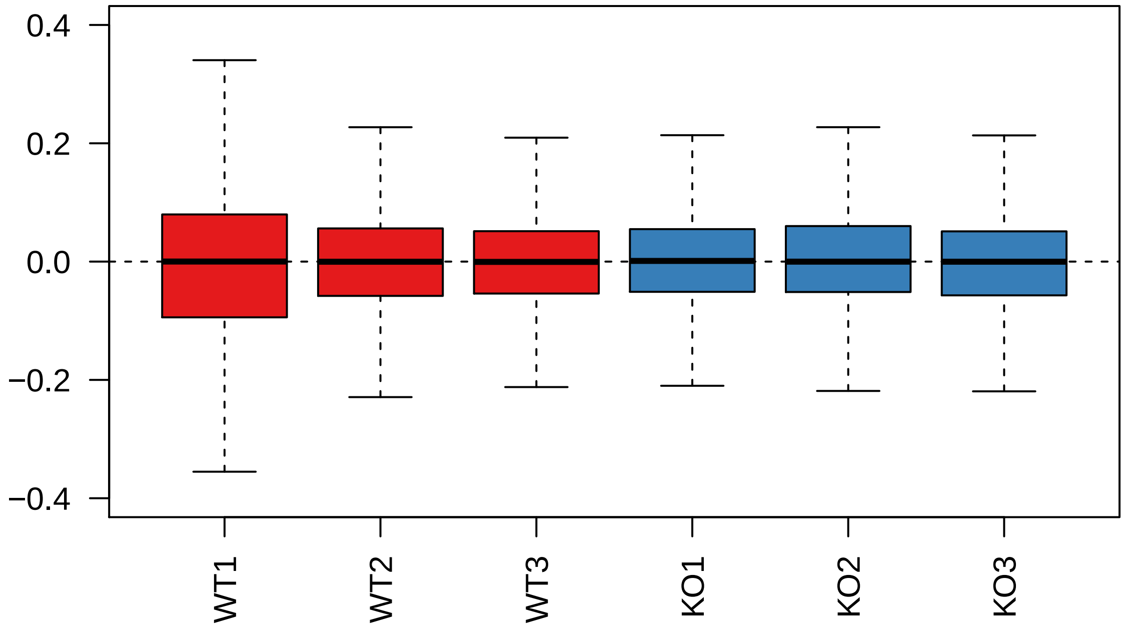


```
plotPCA(filtered, col = colLib)
```

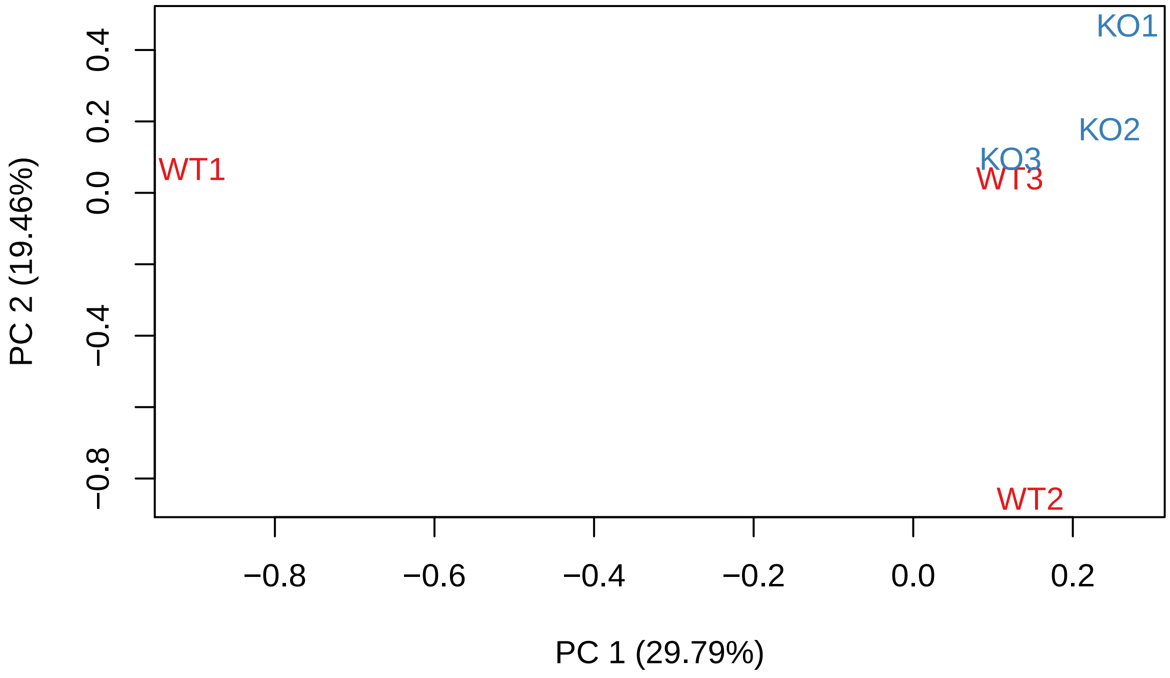


```
groups <- matrix(data = c(1:3, 4:6), nrow = 2, byrow = TRUE)
s <- RUVs(round(filtered), 1:nrow(filtered), k = 1, groups)

plotRLE(s$normalizedCounts, col = colLib, outline = FALSE, ylim = c(-0.4, 0.4), las = 2)
```

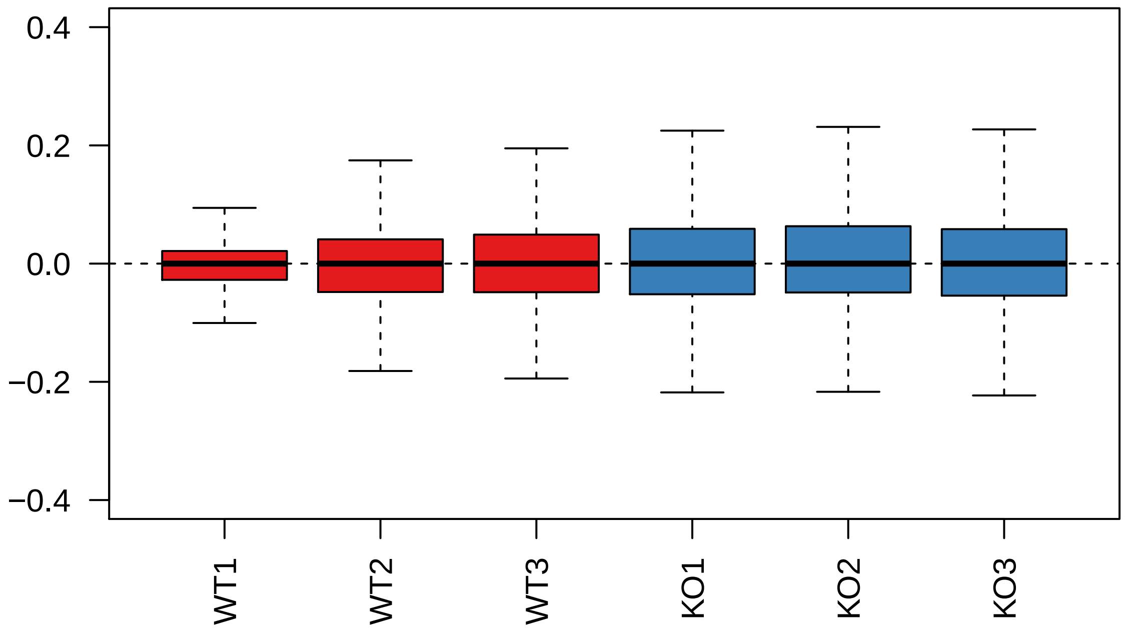


```
plotPCA(s$normalizedCounts, col = colLib)
```

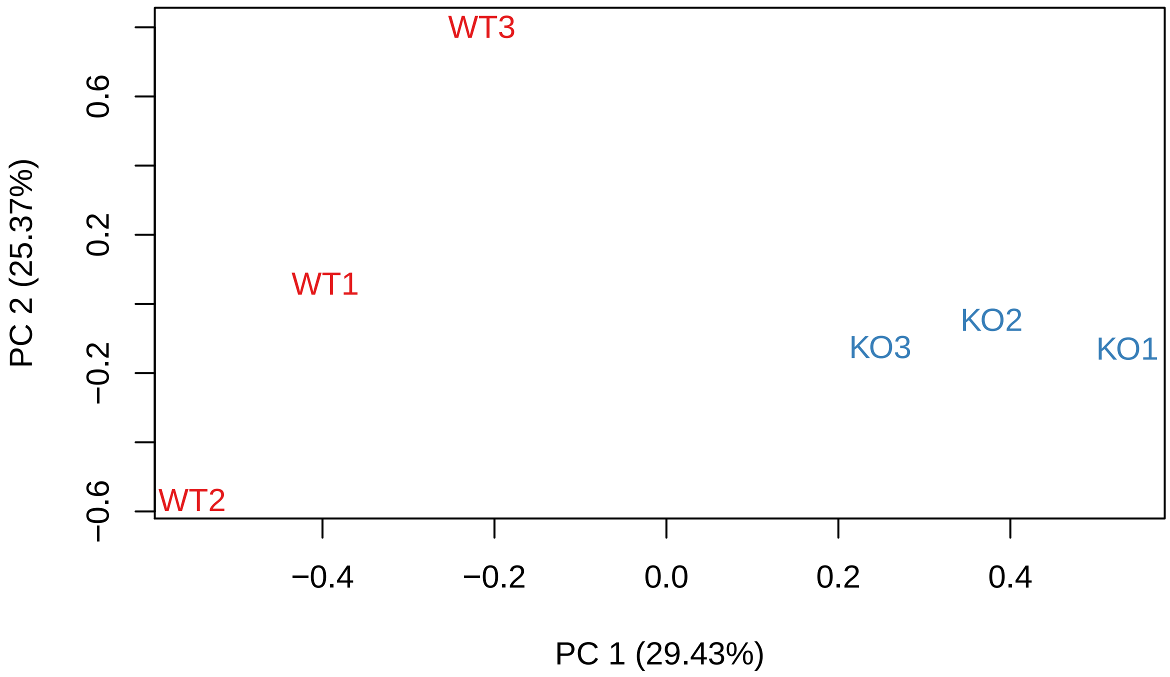

2 GSE60262

2.1 dicer KO small RNA data, DESeq2 normalization

```
data <- read.table("GEO_datasets/GSE60262.txt", header = TRUE, sep = "\t")
dicerKOsmallRNA <- as.matrix(data[, 2:ncol(data)])
x <- as.factor(rep(c("KO", "WT"), each = 3))
colLib <- colors[x]
```

```
boxplot(log(dicerKOsmallRNA + 1), las = 2)
```

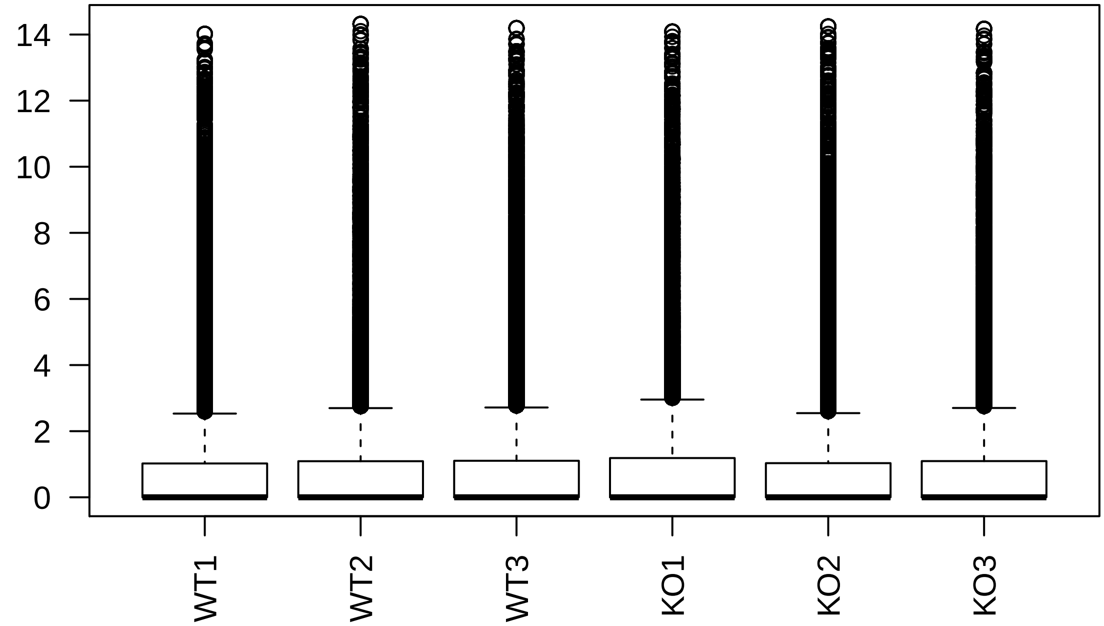


```
filter <- apply(dicerKOsmallRNA, 1, function(x) length(x[which(x > 0)]) > 3)
table(filter)
```

```
## filter
## FALSE  TRUE
## 69701 39407
```

```
filtered <- dicerKOsmallRNA[filter, ]

plotRLE(filtered, col = colLib, outline = FALSE, ylim = c(-1, 1), las = 2)
```

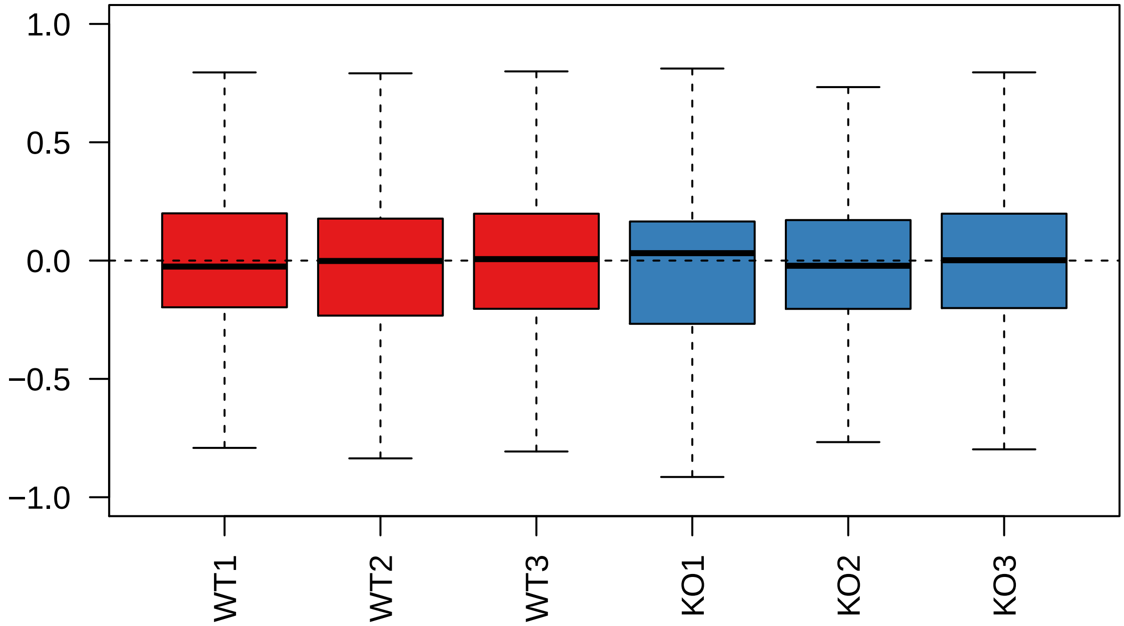

```
plotPCA(filtered, col = colLib)
```

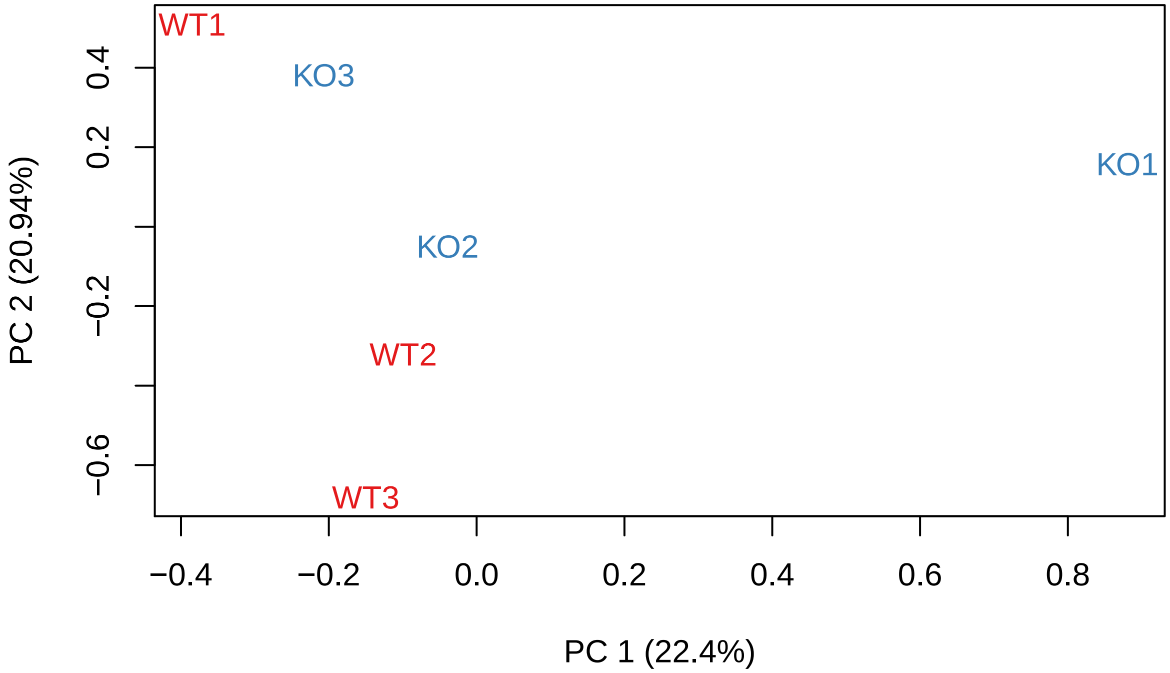


```
groups <- matrix(data = c(1:3, 4:6), nrow = 2, byrow = TRUE)
s <- RUVs(round(filtered), 1:nrow(filtered), k = 2, groups, round = FALSE)

plotRLE(s$normalizedCounts, col = colLib, outline = FALSE, ylim = c(-1, 1), las = 2)
```

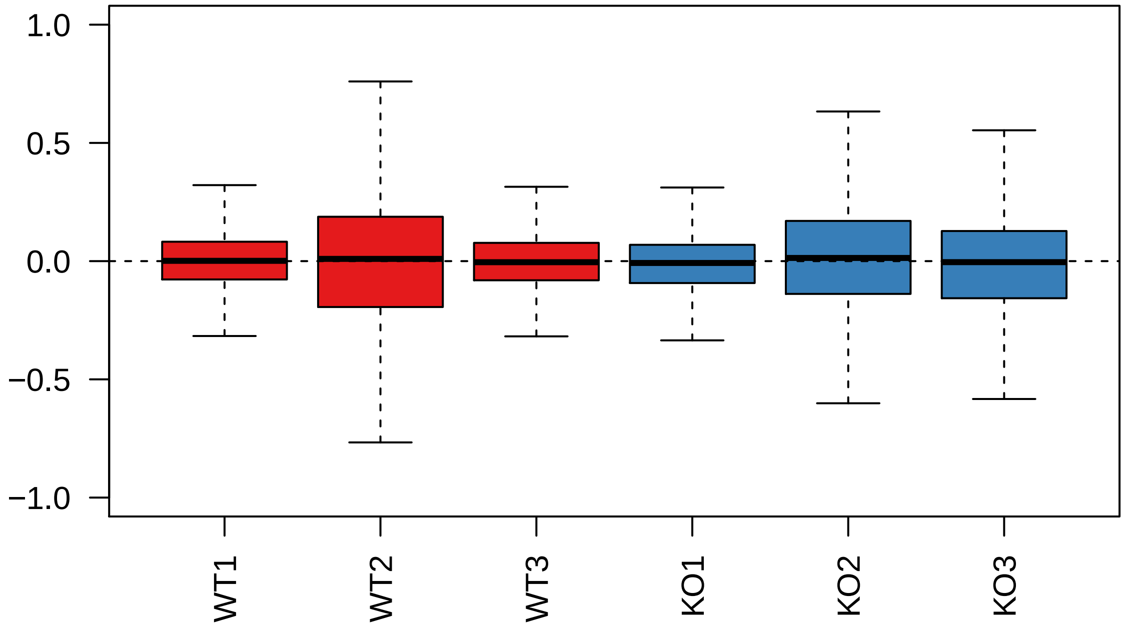


```
plotPCA(s$normalizedCounts, col = colLib)
```

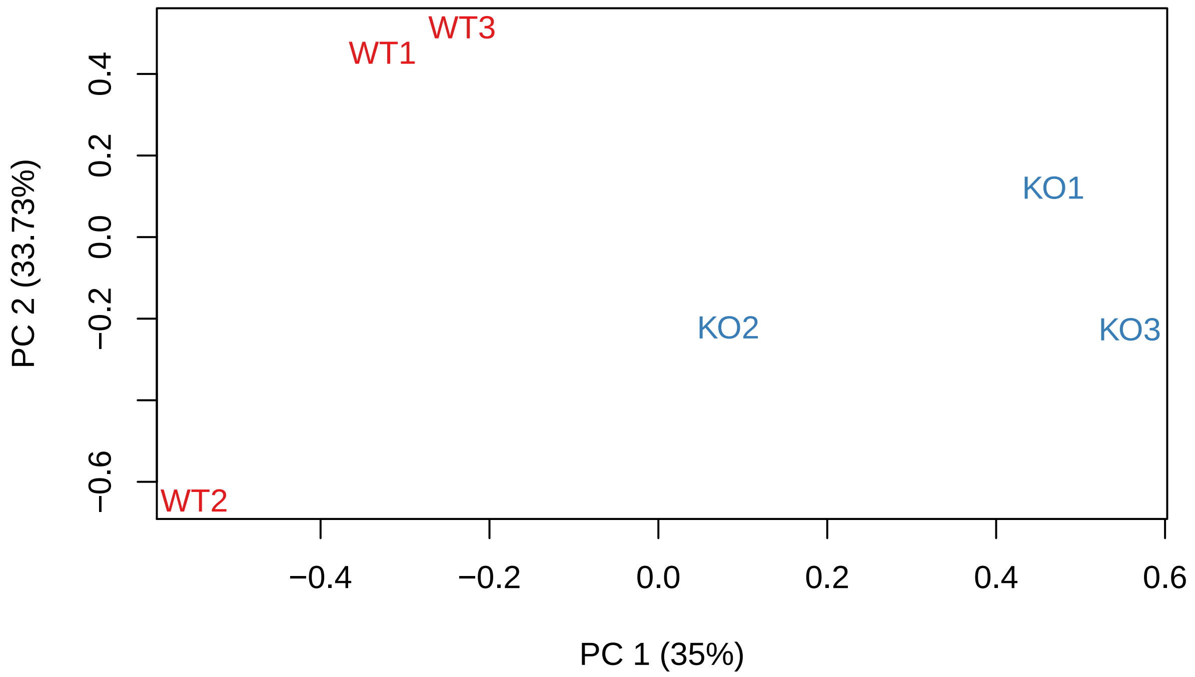

3 GSE58797

3.1 H2A.Z shRNA knckdown FPKM, and FPKM+UQ

```
data <- read.table("GEO_datasets/GSE58797.txt", header = TRUE, sep = "\t")
H2AZRNA <- as.matrix(data[, 2:10])
x <- as.factor(rep(c("Control", "shRNA", "FC"), each = 3))
colLib <- colors[x]

boxplot(log(H2AZRNA + 1), las = 2)
```

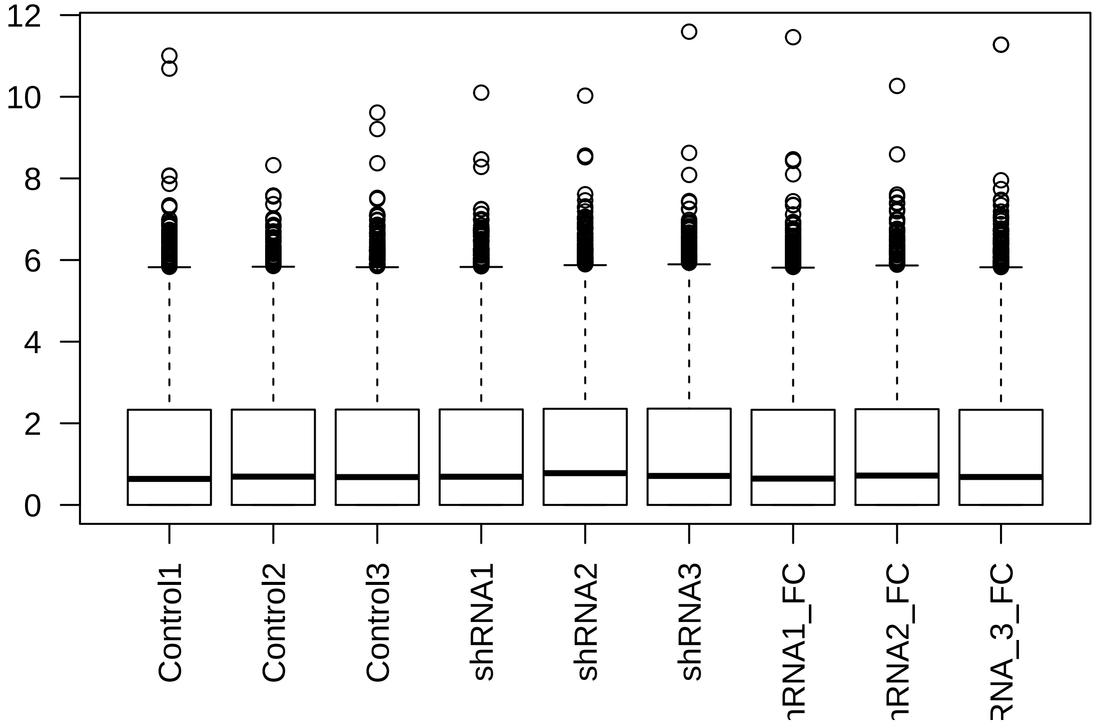


```
filter <- apply(H2AZRNA, 1, function(x) length(x[which(x > 0)]) > 3)
table(filter)
```

```
## filter
## FALSE  TRUE
##  7518 16971
```

```
filtered <- H2AZRNA[filter, ]

H2AZFPKMuq <- betweenLaneNormalization(filtered, which = "upper", round = FALSE)

boxplot(log(H2AZFPKMuq + 1), las = 2)
```

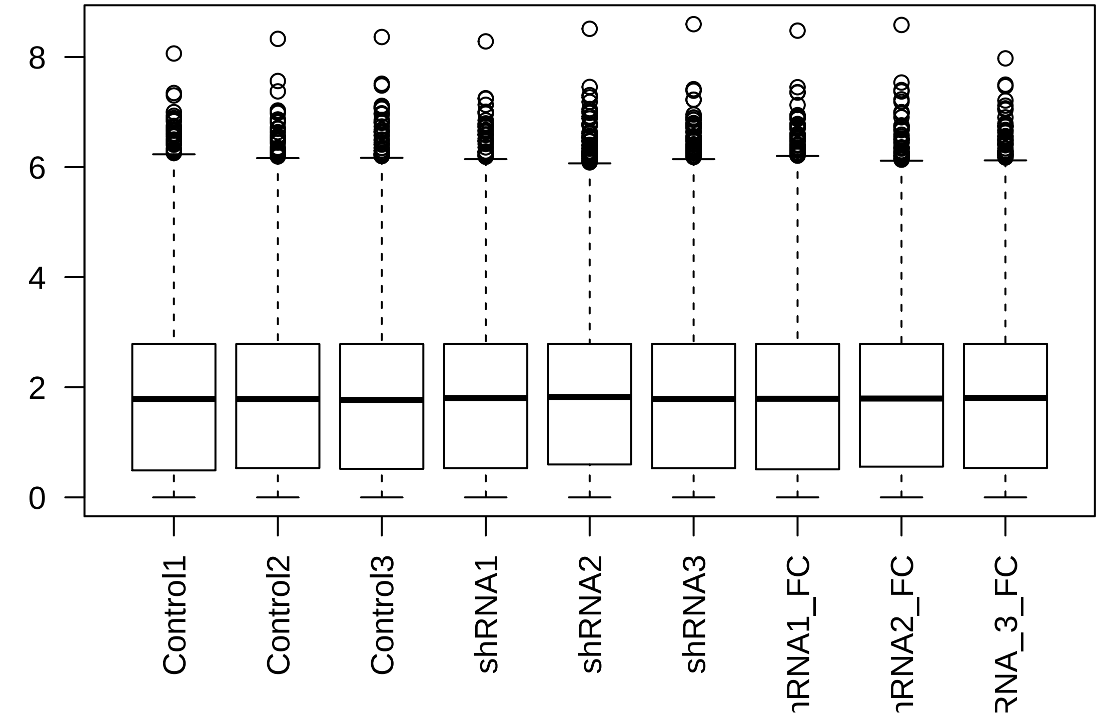


```
plotRLE(H2AZFPKMuq, col = colLib, outline = FALSE, ylim = c(-0.2, 0.2), las = 2)
```

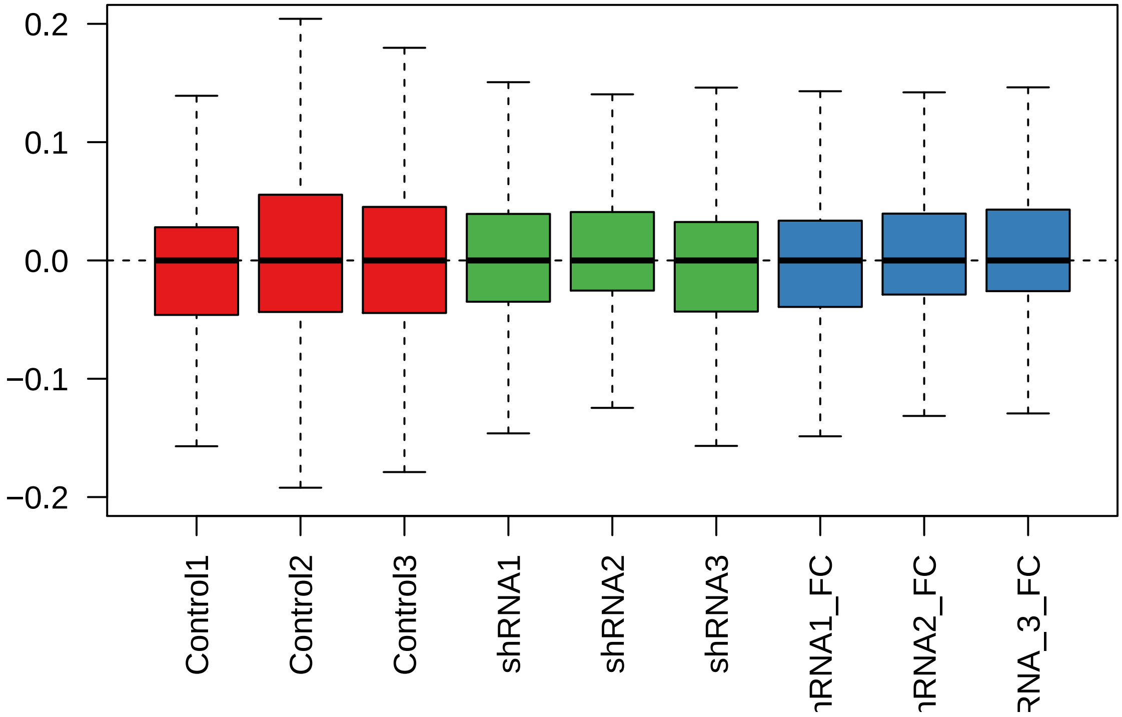


```
plotPCA(H2AZFPKMuq, col = colLib)
```

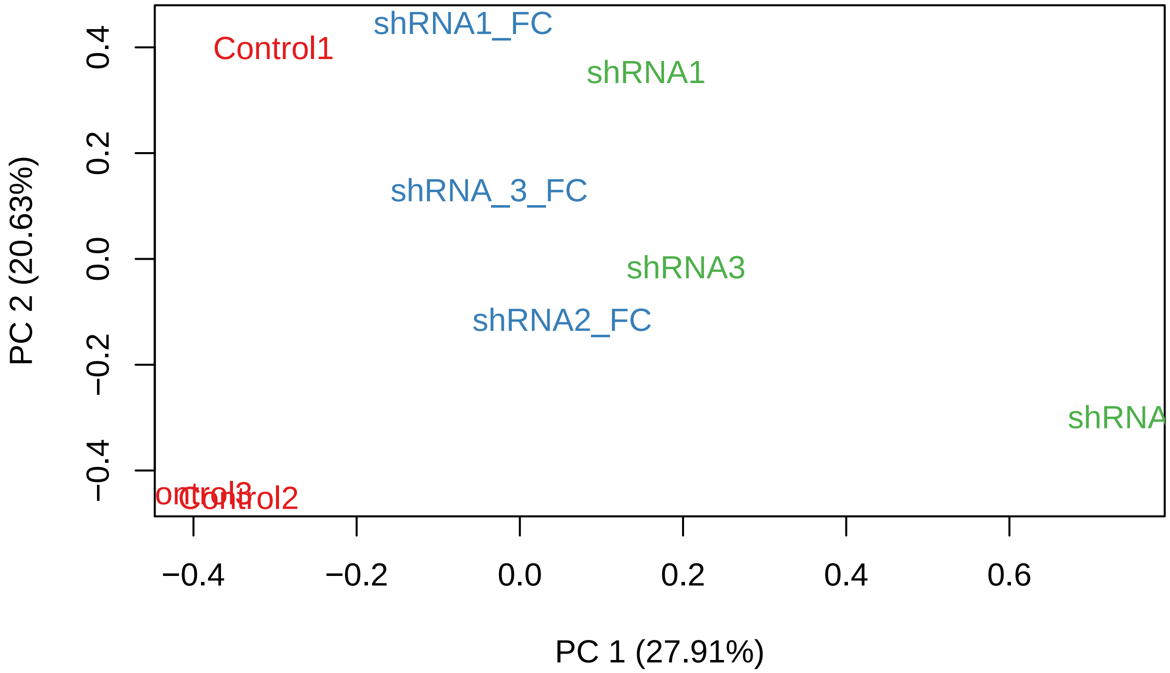


```
groups <- matrix(data = c(1:3, 4:6, 7:9), nrow = 3, byrow = TRUE)
s <- RUVs(round(H2AZFPKMuq), 1:nrow(H2AZFPKMuq), k = 1, groups, round = FALSE)

plotRLE(s$normalizedCounts, col = colLib, outline = FALSE, ylim = c(-0.2, 0.2), las = 2)
```

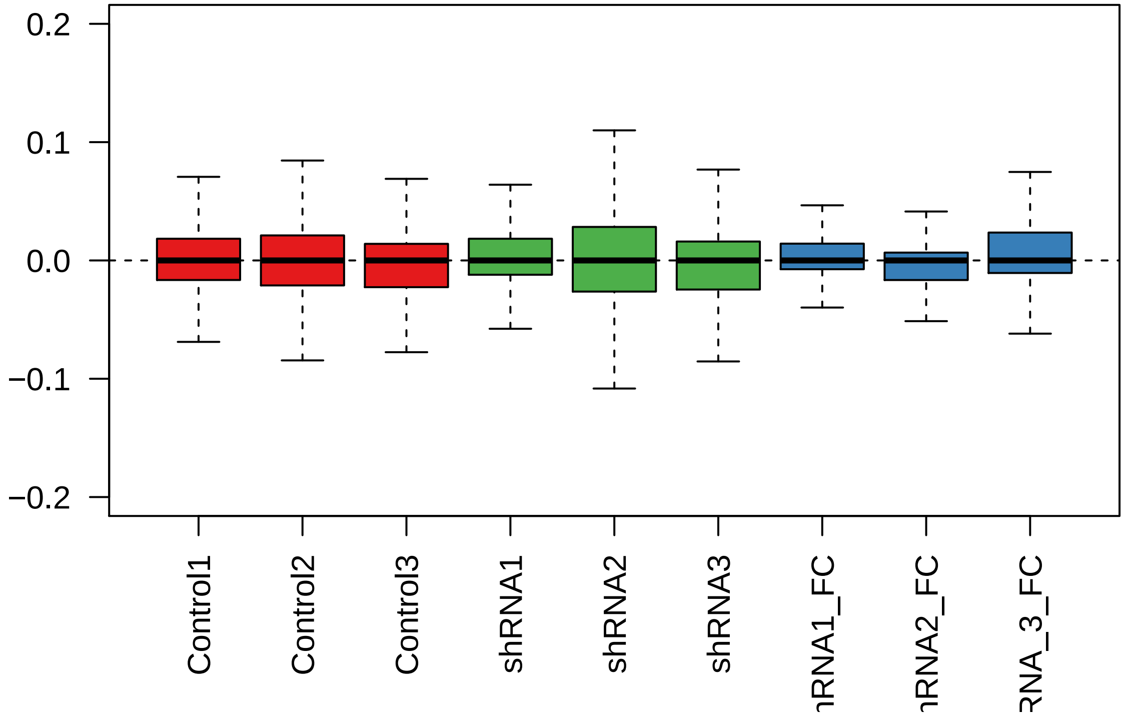


```
plotPCA(s$normalizedCounts, col = colLib)
```

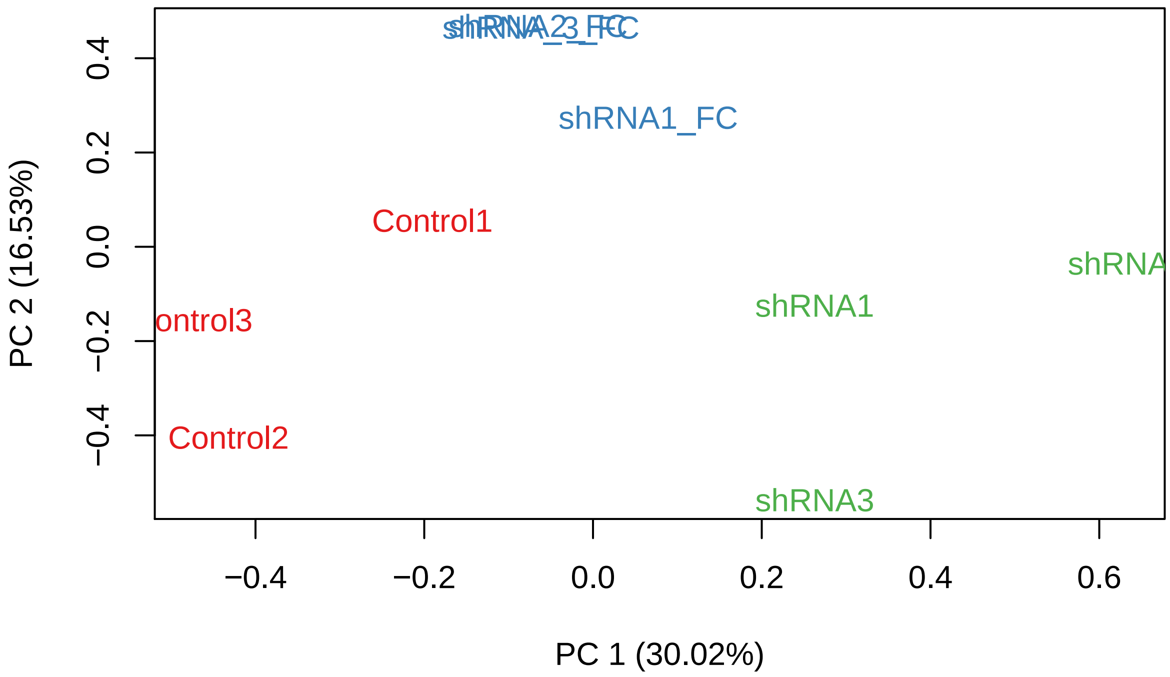

4 GSE61915

4.1 aging 3M-24 M SE filter >10 reads, DESeq

```
data <- read.table("GEO_datasets/GSE61915.txt", header = TRUE, sep = "\t")
agingRNASE <- as.matrix(data[, 2:ncol(data)])
x <- as.factor(c(rep("Young", 5), rep("Old", 6)))
colLib <- colors[x]

boxplot(log(agingRNASE + 1), las = 2)
```

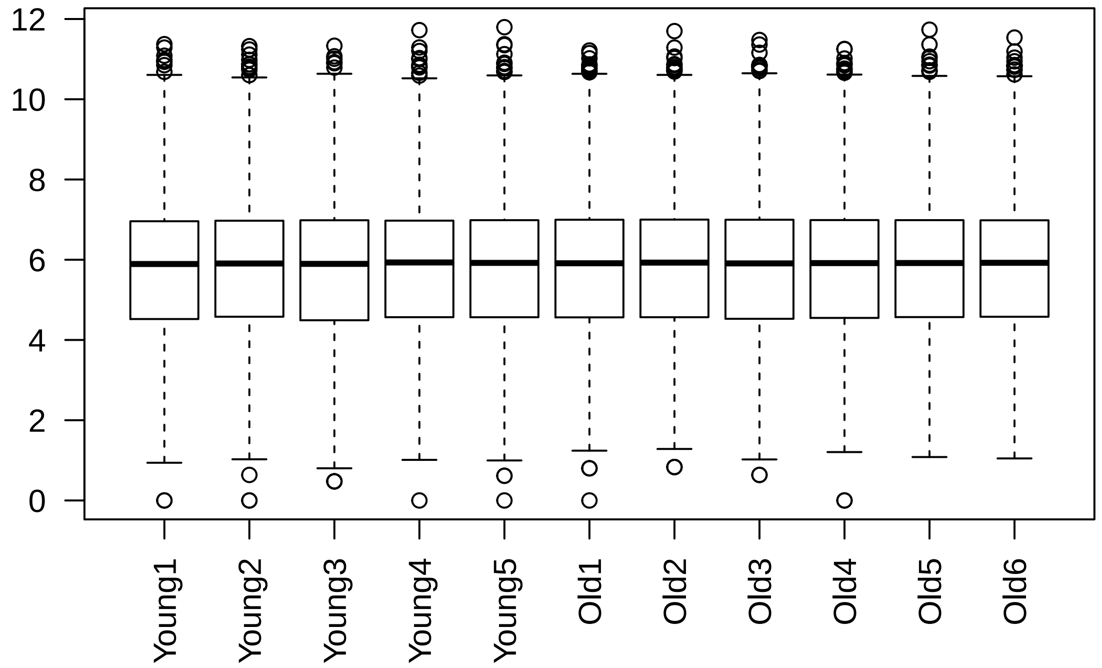


```
filter <- apply(agingRNASE, 1, function(x) length(x[which(x > 0)]) > 3)
table(filter)
```

```
## filter
##  TRUE
## 15436
```

```
filtered <- agingRNASE[filter, ]

plotRLE(filtered, col = colLib, outline = FALSE, ylim = c(-0.5, 0.5), las = 2)
```

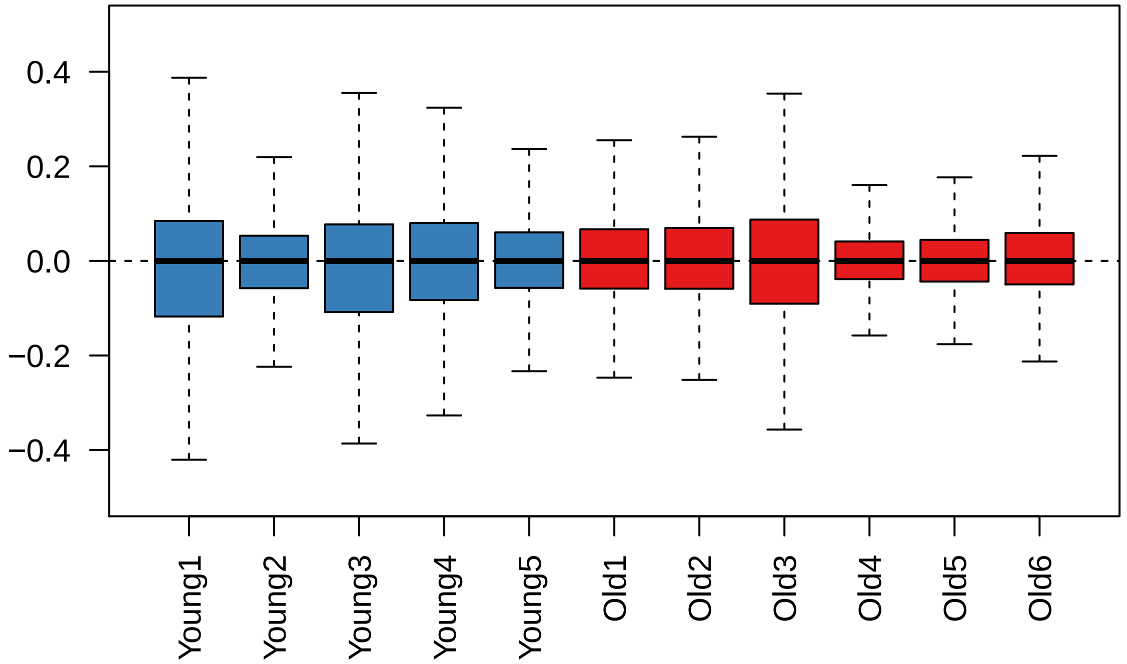


```
plotPCA(filtered, col = colLib)
```

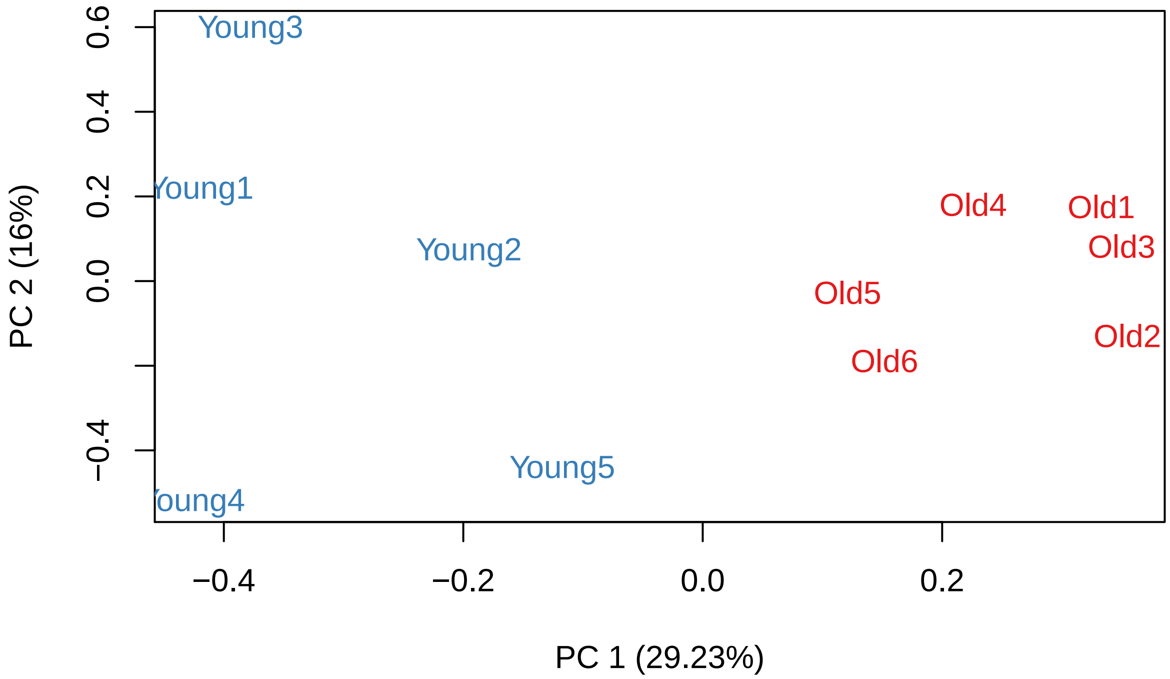


```
groups <- matrix(data = c(1:5, -1, 6:11), nrow = 2, byrow = TRUE)
s <- RUVs(round(filtered), 1:nrow(filtered), k = 1, groups)

plotRLE(s$normalizedCounts, col = colLib, outline = FALSE, ylim = c(-0.5, 0.5), las = 2)
```

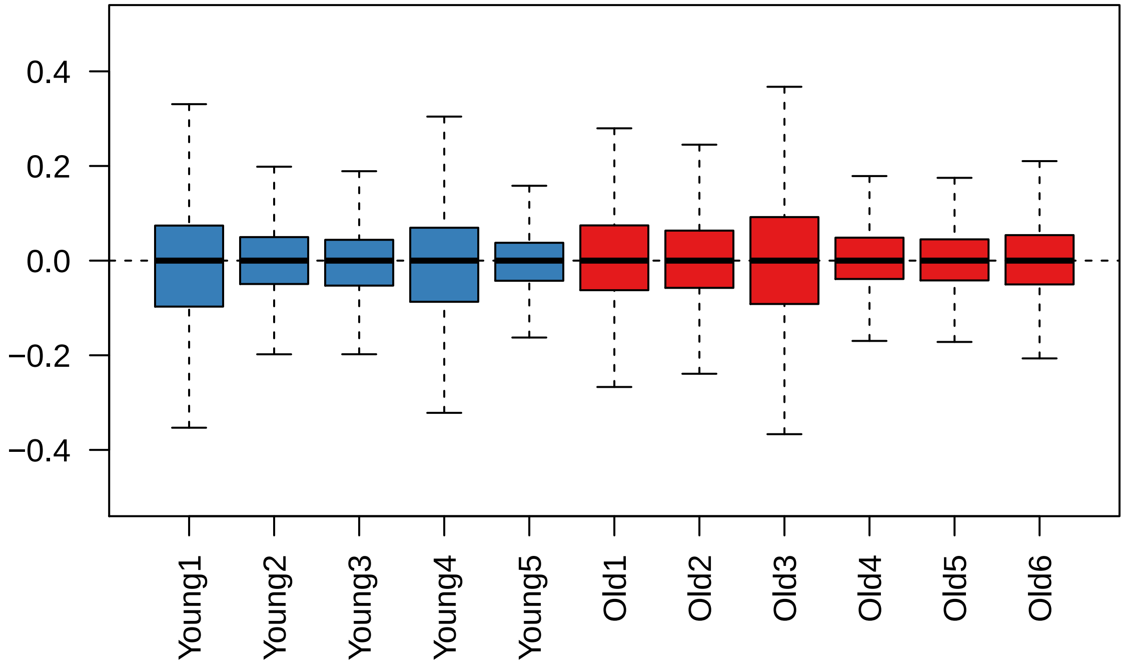


```
plotPCA(s$normalizedCounts, col = colLib)
```

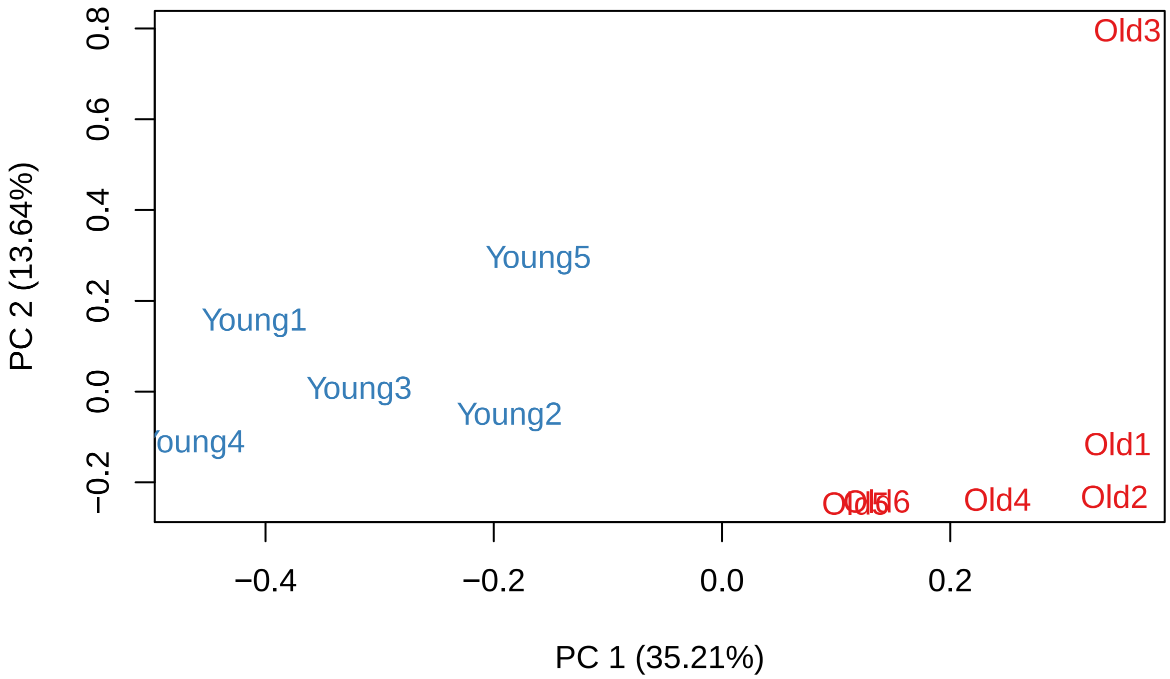

5 GSE53380

5.1 Kat2a KO and NOR, UQ

```
data <- read.table("GEO_datasets/GSE53380.txt", header = TRUE, sep = "\t")
KO_NORRNA <- as.matrix(data[, 2:ncol(data)])
x <- as.factor(c(rep("Control", 6), rep("NOR", 5), rep("KO", 6), rep("KO_NOR", 7)))
colLib <- colors[x]

boxplot(log(KO_NORRNA + 1), las = 2)
```

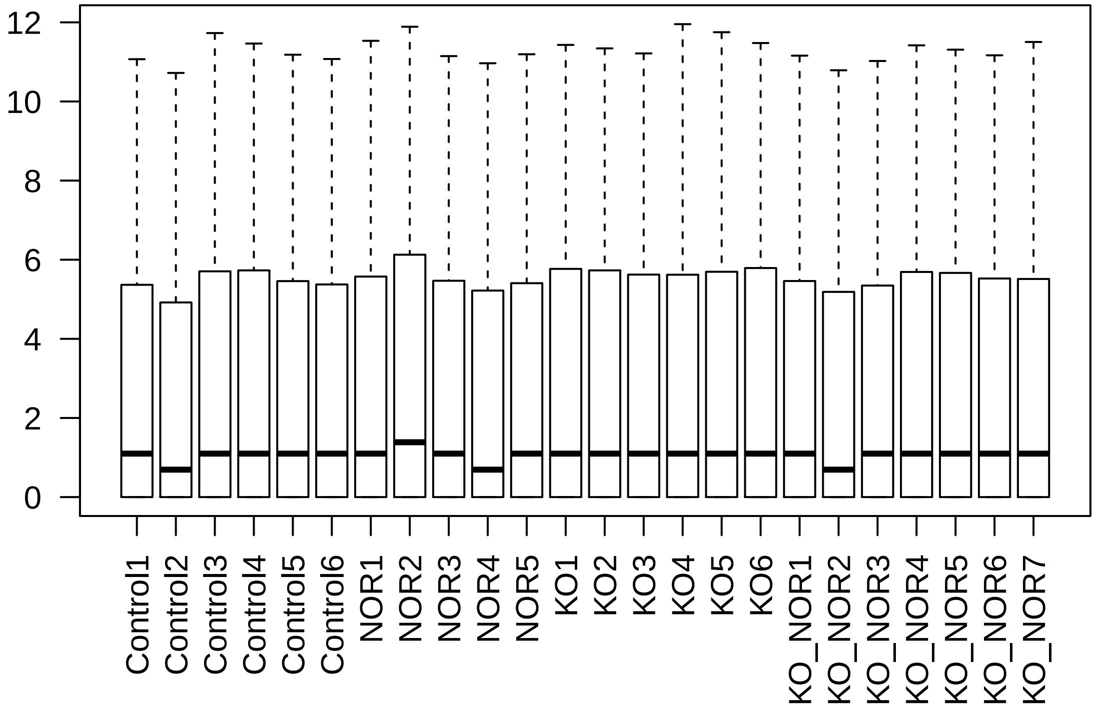


```
filter <- apply(KO_NORRNA, 1, function(x) length(x[which(x > 0)]) > 3)
table(filter)
```

```
## filter
## FALSE  TRUE
## 14203 24152
```

```
filtered <- KO_NORRNA[filter, ]

uq <- betweenLaneNormalization(filtered, which = "upper")
boxplot(log(uq + 1), las = 2)
```

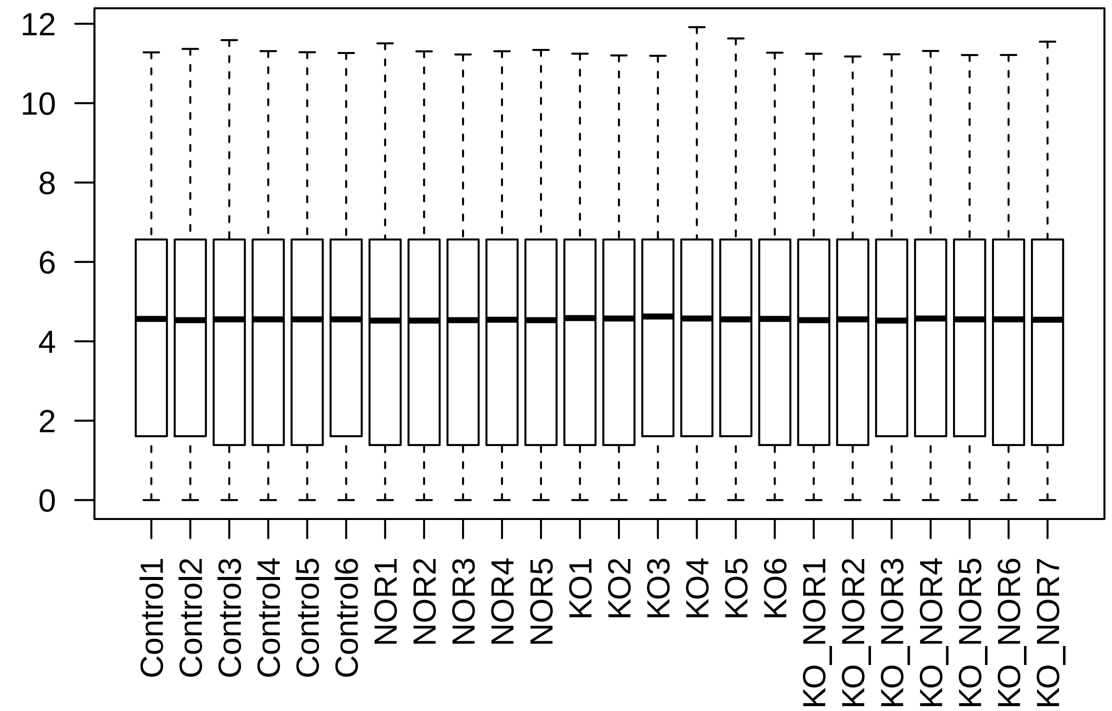


```
plotRLE(uq, col = colLib, outline = FALSE, ylim = c(-0.6, 0.6), las = 2)
```

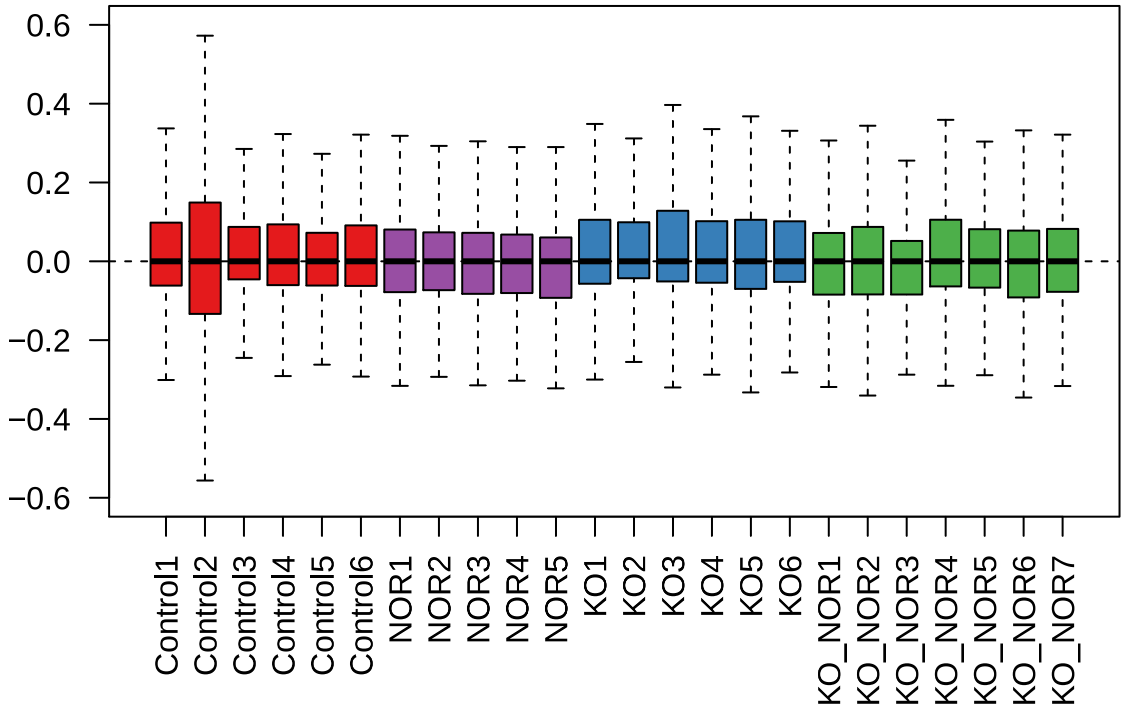


```
plotPCA(uq, col = colLib)
```

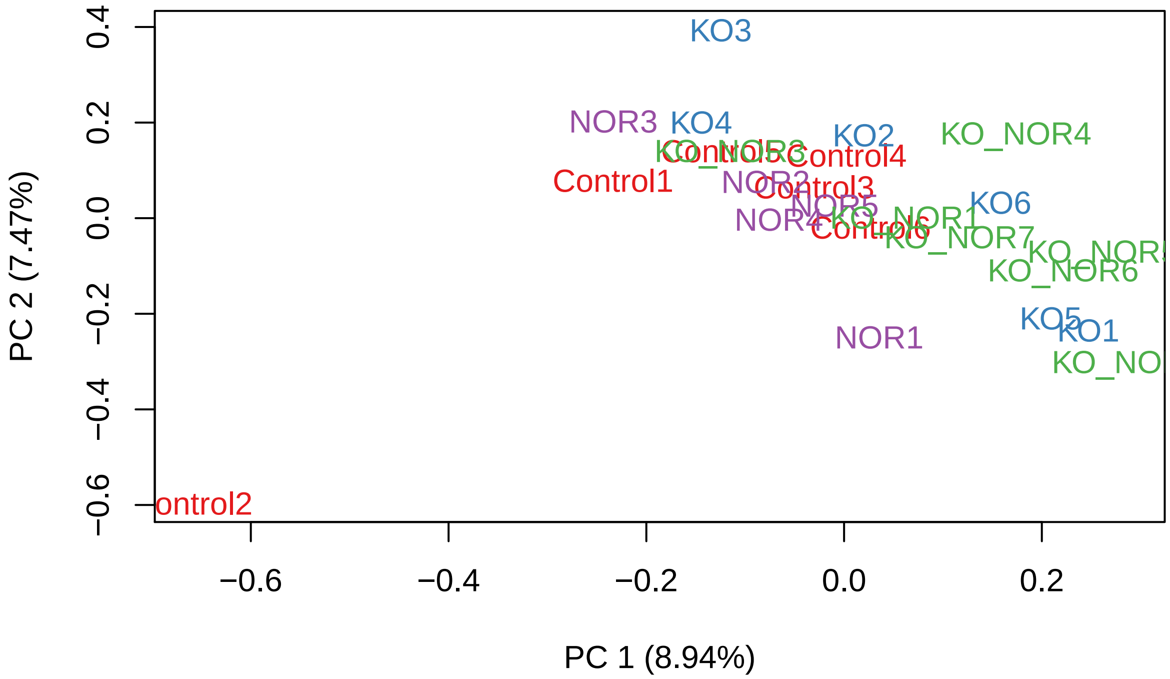


```r
groups <- matrix(data = c(1:6, -1, 7:11, -1, -1, 12:17, -1, 18:24), nrow = 4, byrow = TRUE)
s <- RUVs(uq, 1:nrow(uq), k = 2, groups)

plotRLE(s$normalizedCounts, col = colLib, outline = FALSE, ylim = c(-0.6, 0.6), las = 2)
```

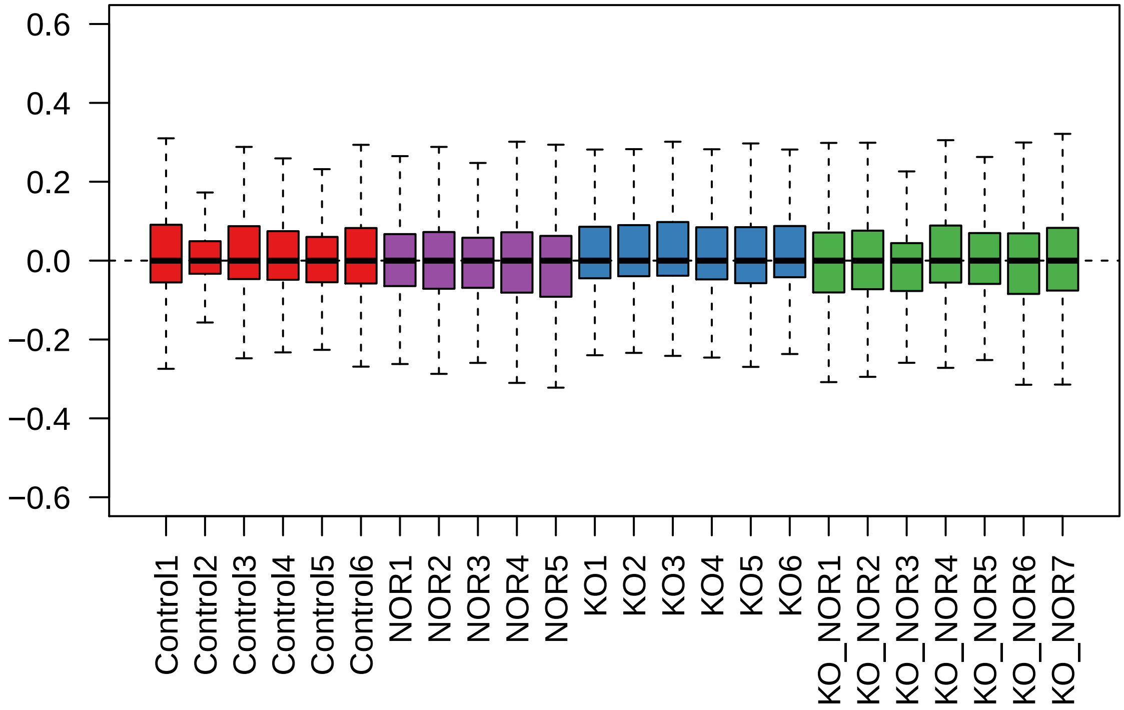


```r
plotPCA(s$normalizedCounts, col = colLib)
```

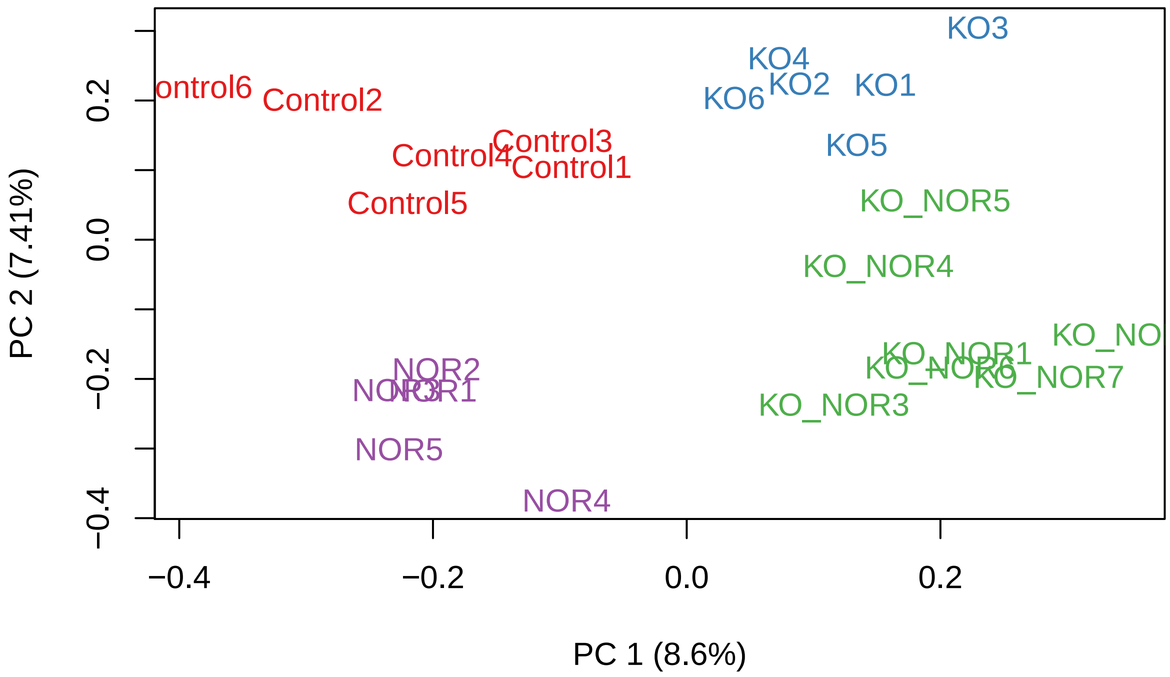

6 GSE65159

6.1 AD mouse model, 2 time points

```
data <- read.table("GEO_datasets/GSE65159.txt", header = TRUE, sep = "\t")
AD_RNA <- as.matrix(data[, 2:ncol(data)])
x <- as.factor(rep(c("Control6wk", "AD6wk", "Control2wk", "AD2wk"), each = 3))
colLib <- colors[x]

boxplot(log(AD_RNA + 1), las = 2)
```

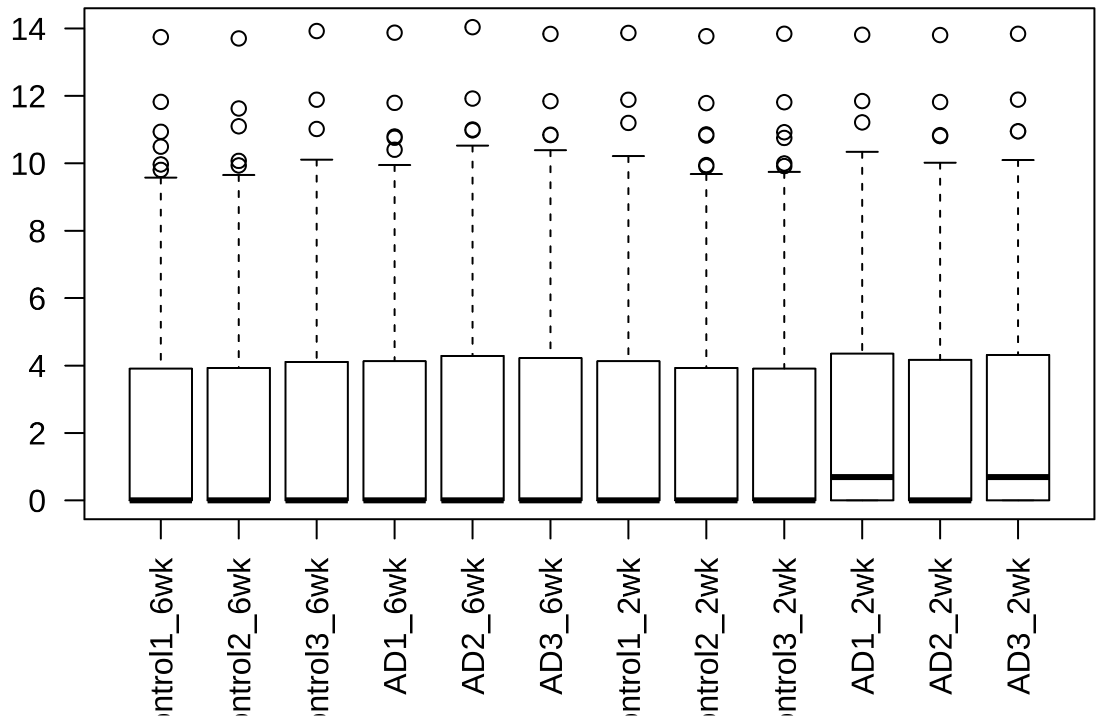


```
filter <- apply(AD_RNA, 1, function(x) length(x[which(x > 0)]) > 3)
table(filter)
```

```
## filter
## FALSE  TRUE
## 18477 19514
```

```
filtered <- AD_RNA[filter, ]

uq <- betweenLaneNormalization(filtered, which = "upper")
boxplot(log(uq + 1), las = 2)
```

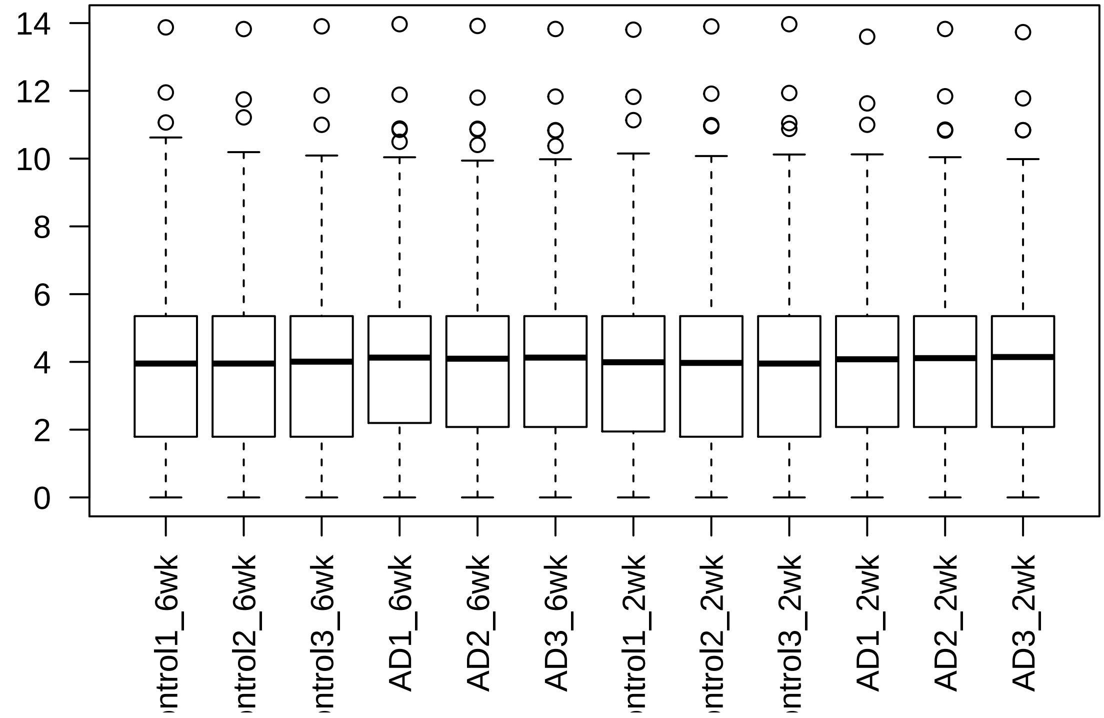


```r
plotRLE(uq, col = colLib, outline = FALSE, ylim = c(-1, 1), las = 2)
```

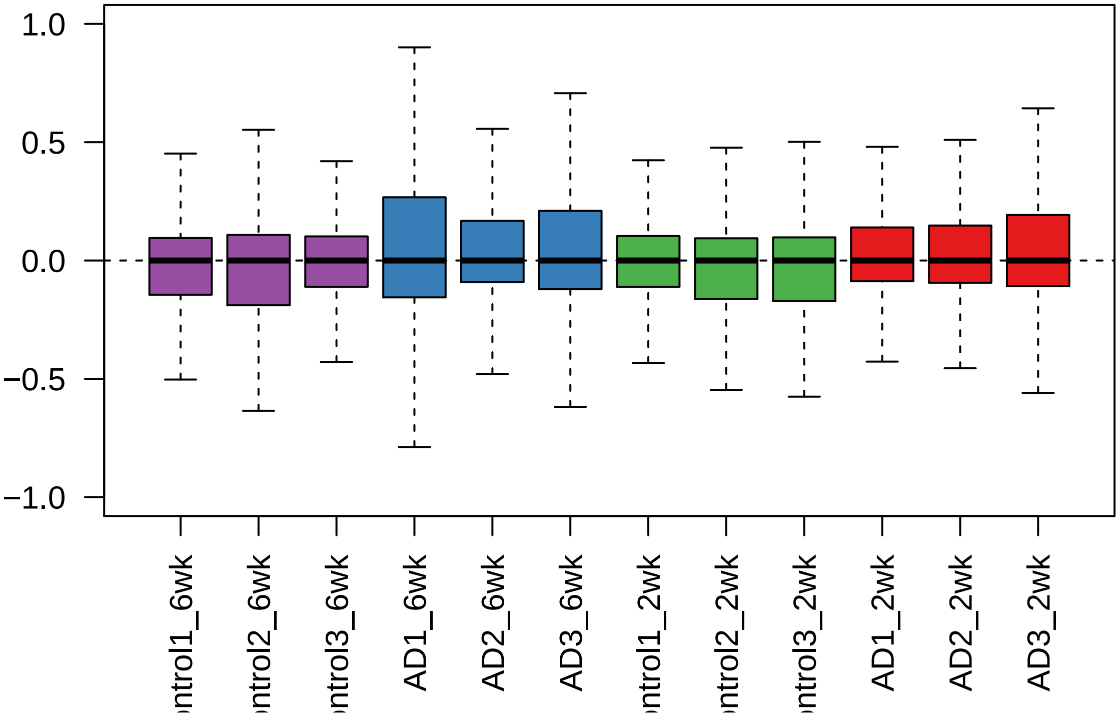


```r
plotPCA(uq, col = colLib)
```

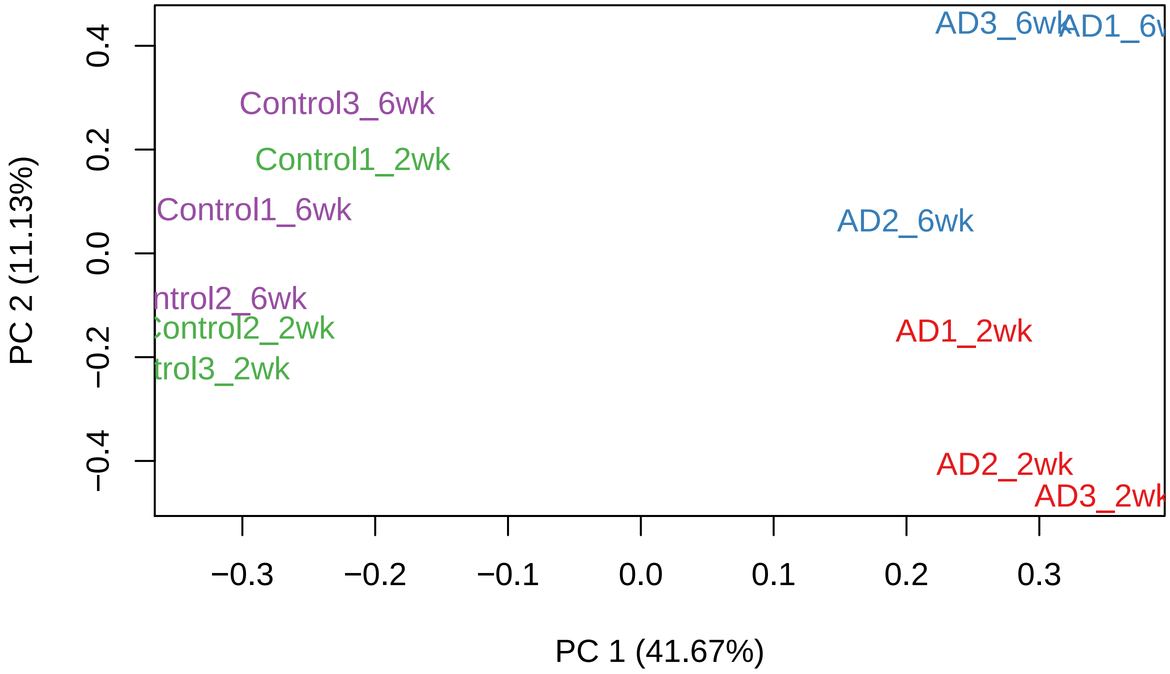


```
groups <- matrix(data = c(1:3, 4:6, 7:9, 10:12), nrow = 4, byrow = TRUE)
s <- RUVs(uq, 1:nrow(uq), k = 1, groups)

plotRLE(s$normalizedCounts, col = colLib, outline = FALSE, ylim = c(-1, 1), las = 2)
```

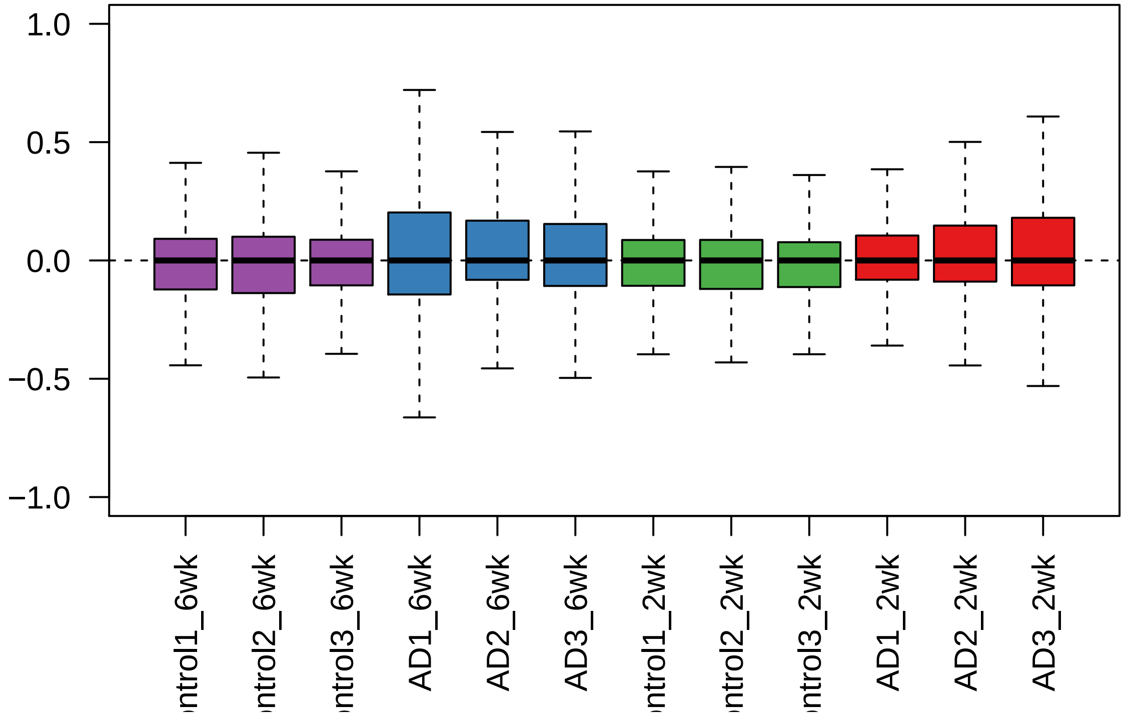


```
plotPCA(s$normalizedCounts, col = colLib)
```

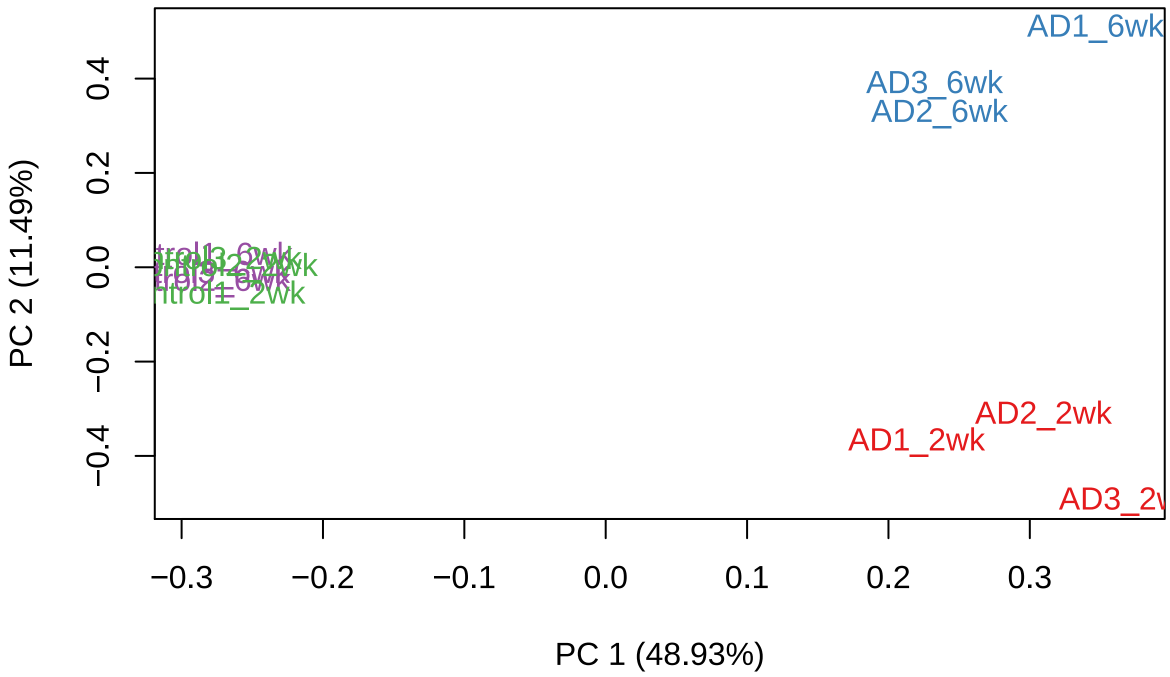

7 GSE58343

7.1 ribosome bound, FPKM PE and SE

```
data <- read.table("GEO_datasets/GSE58343.txt", header = TRUE, sep = "\t")
Ribosome_RNA <- as.matrix(data[, 2:ncol(data)])
x <- as.factor(c(rep("HC", 10), rep("FC", 12)))
colLib <- colors[x]

boxplot(log(Ribosome_RNA + 1), las = 2)
```

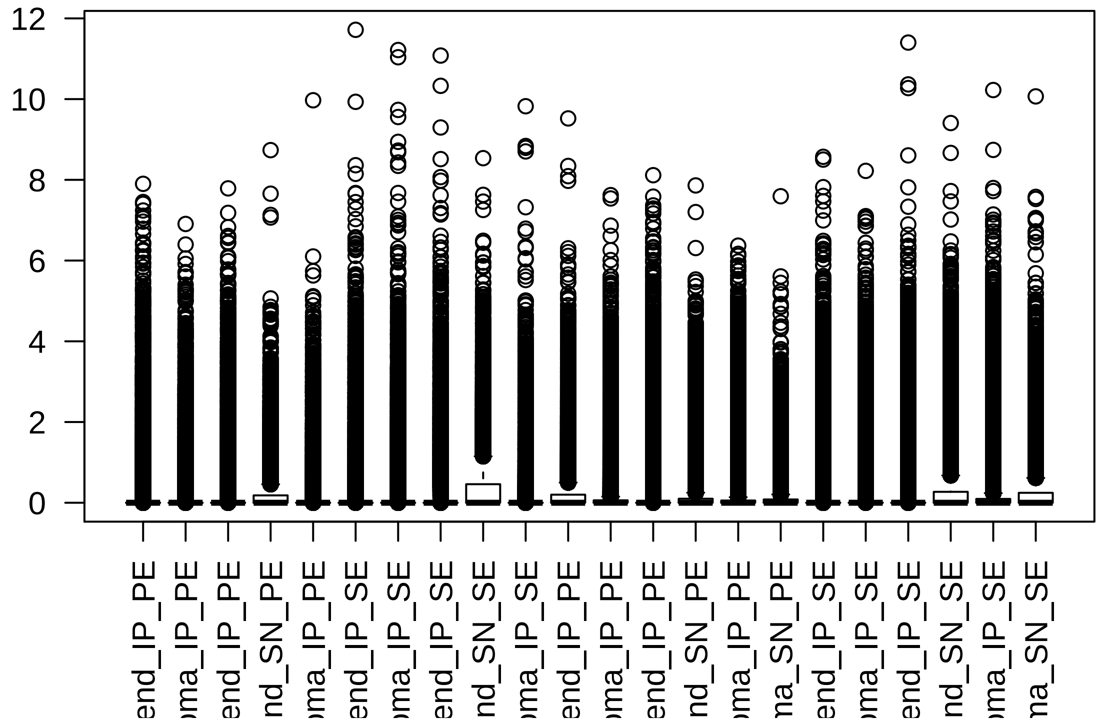


```
filter <- apply(Ribosome_RNA, 1, function(x) length(x[which(x > 0)]) > 3)
table(filter)
```

```
## filter
## FALSE  TRUE
## 16117 13731
```

```
filtered <- Ribosome_RNA[filter, ]

boxplot(log(filtered + 1), las = 2)
```

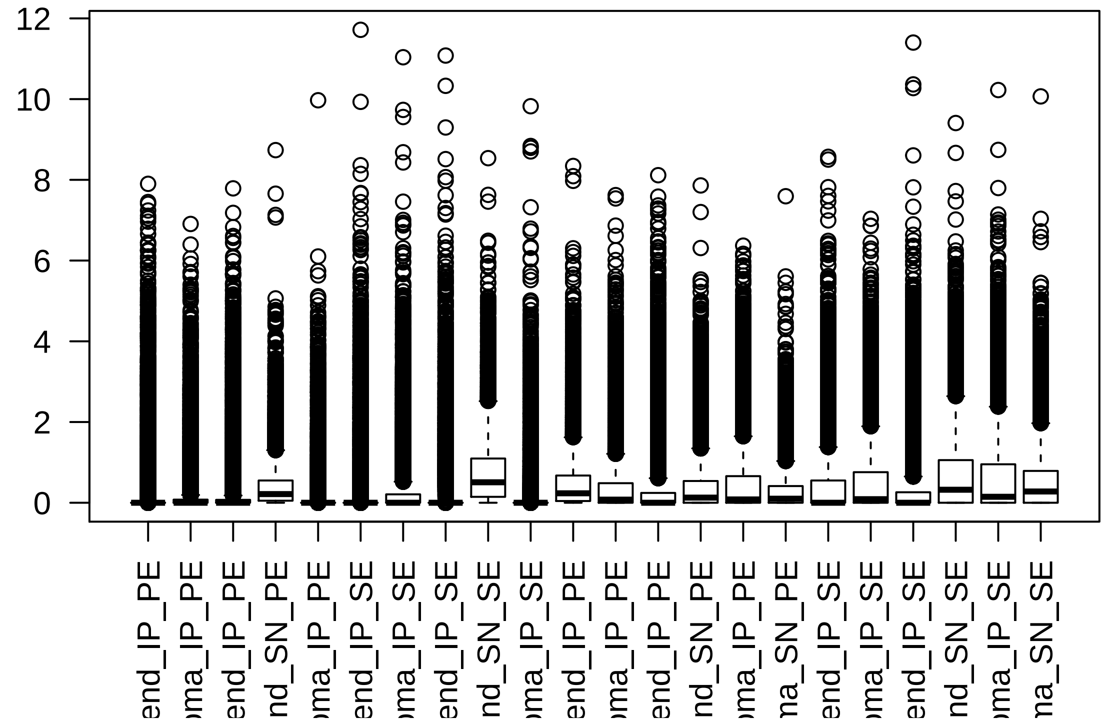


```r
plotRLE(filtered, col = colLib, outline = FALSE, ylim = c(-1, 2), las = 2)
```

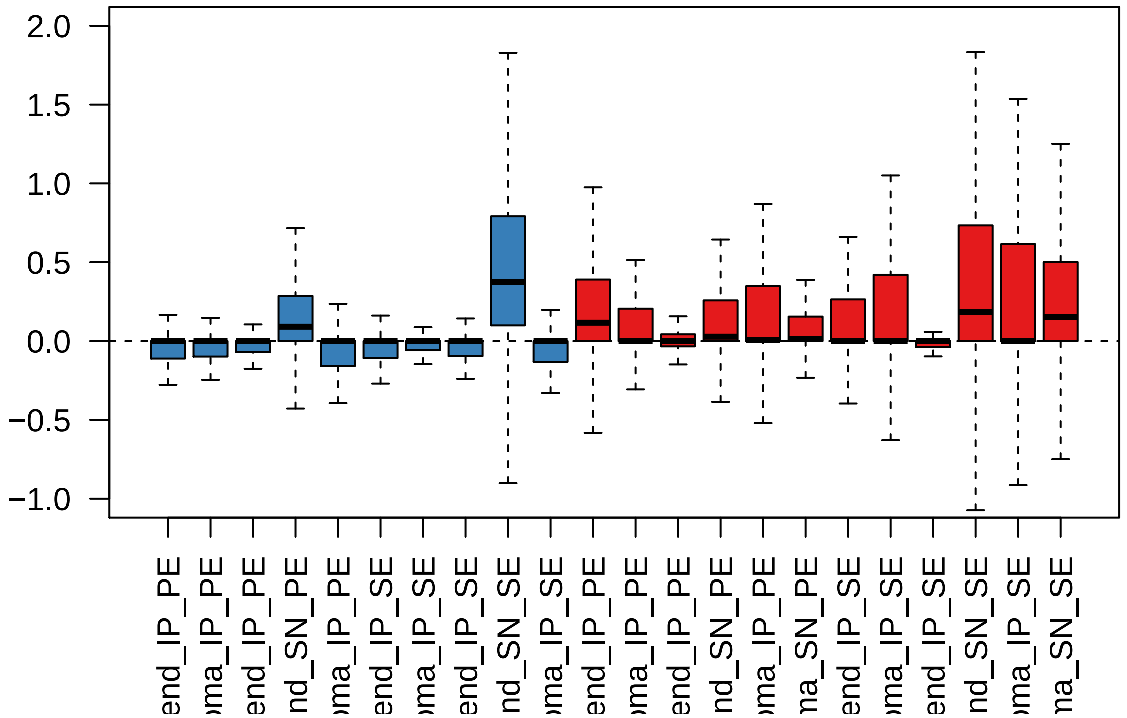


```r
plotPCA(filtered, col = colLib)
```

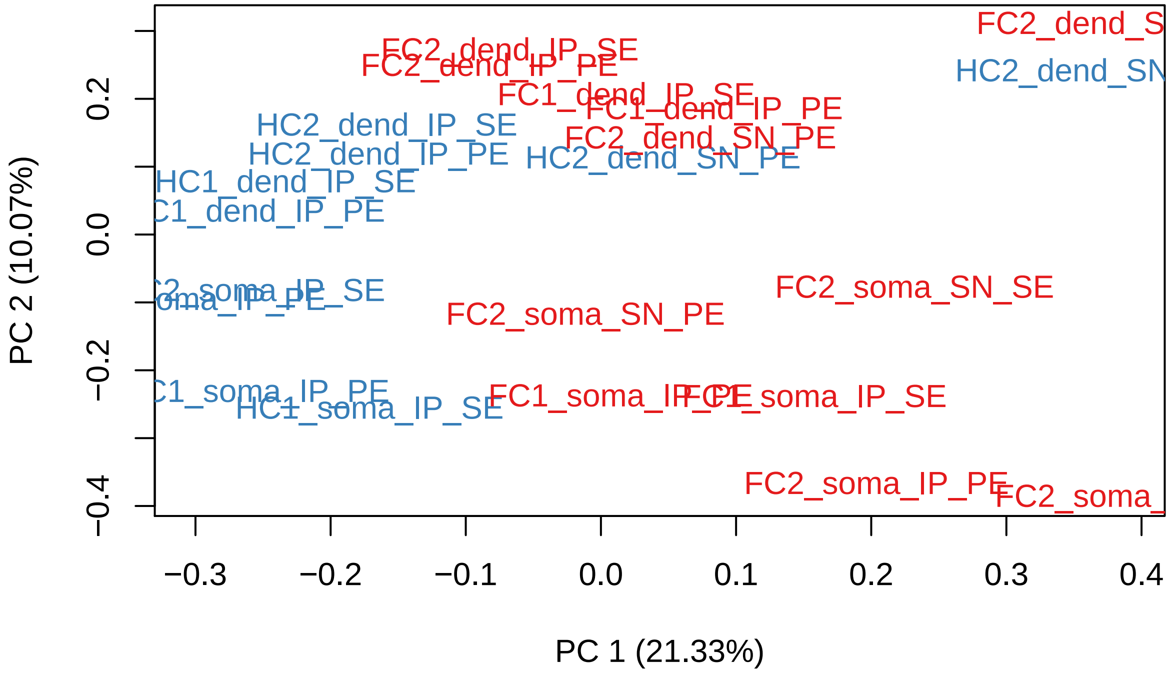


```r
groups <- matrix(data = c(1, 3, 6, 8, 4, 9, -1, -1, 2, 5, 7, 10, 11, 13, 17, 19,
    14, 20, -1, -1, 16, 22, -1, -1, 12, 15, 18, 21), nrow = 7, byrow = TRUE)
s <- RUVs(round(filtered), 1:nrow(filtered), k = 1, groups, round = FALSE)

plotRLE(s$normalizedCounts, col = colLib, outline = FALSE, ylim = c(-1, 2), las = 2)
```

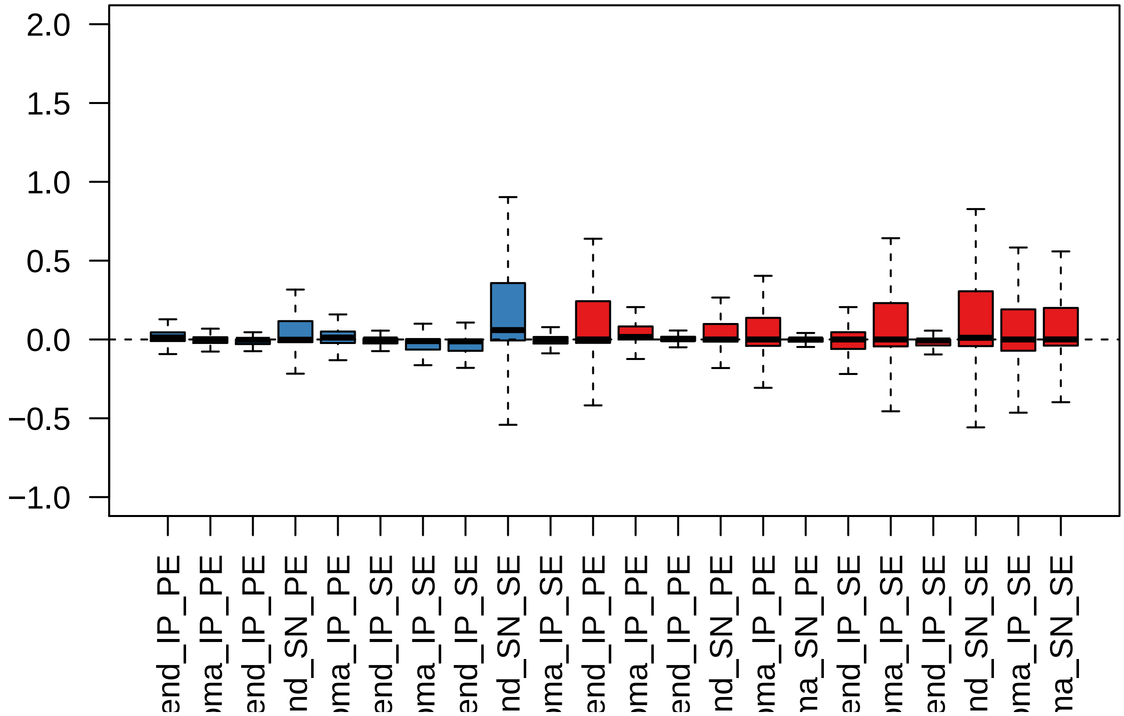


```r
plotPCA(s$normalizedCounts, col = colLib)
```

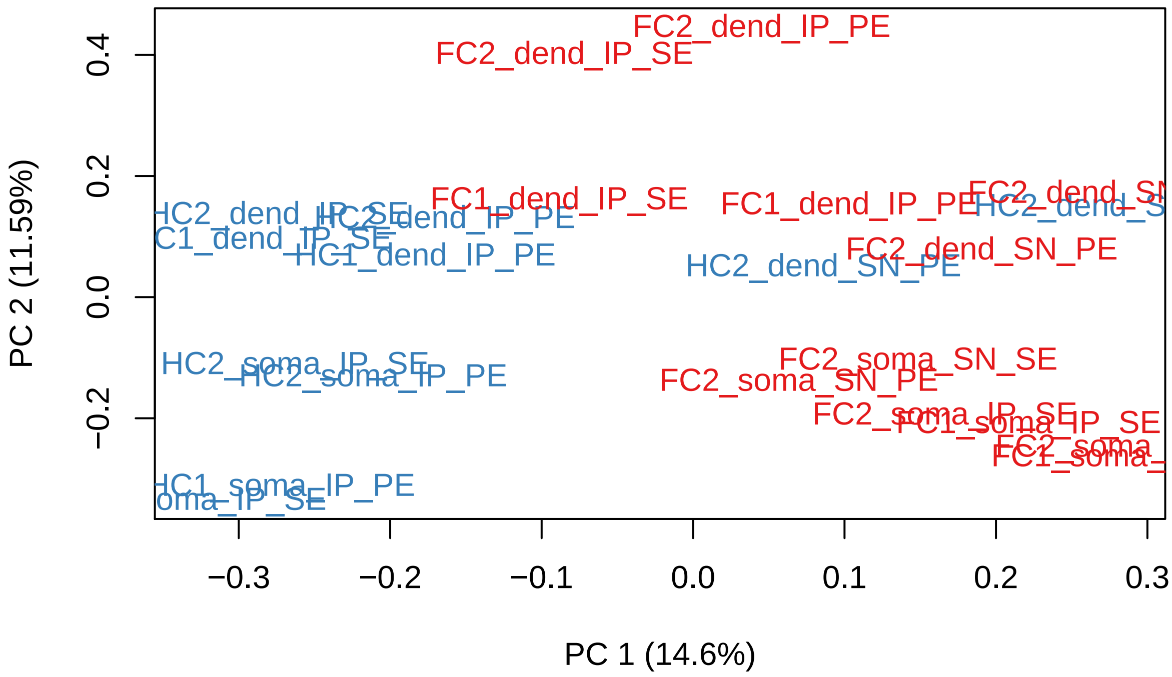

8 Session Info

```
sessionInfo()
```

```
## R version 3.2.0 (2015-04-16)
## Platform: x86_64-apple-darwin13.4.0 (64-bit)
## Running under: OS X 10.10.3 (Yosemite)
##
## locale:
## [1] en_US.UTF-8/en_US.UTF-8/en_US.UTF-8/C/en_US.UTF-8/en_US.UTF-8
##
## attached base packages:
## [1] stats4    parallel  stats     graphics  grDevices utils
## [7] datasets  methods   base
##
## other attached packages:
##  [1] RColorBrewer_1.1-2      RUVSeq_1.2.0
##  [3] edgeR_3.10.2            limma_3.24.5
##  [5] EDASeq_2.2.0            ShortRead_1.26.0
##  [7] GenomicAlignments_1.4.1 Rsamtools_1.20.4
##  [9] GenomicRanges_1.20.4    GenomeInfoDb_1.4.0
## [11] Biostrings_2.36.1       XVector_0.8.0
## [13] IRanges_2.2.2           S4Vectors_0.6.0
## [15] BiocParallel_1.2.2      Biobase_2.28.0
## [17] BiocGenerics_0.14.0     BiocStyle_1.6.0
##
## loaded via a namespace (and not attached):
##  [1] formatR_1.2          futile.logger_1.4.1
##  [3] R.methodsS3_1.7.0    futile.options_1.0.0
##  [5] bitops_1.0-6         R.utils_2.1.0
```

```
##  [7] tools_3.2.0          zlibbioc_1.14.0
##  [9] digest_0.6.8         RSQLite_1.0.0
## [11] annotate_1.46.0      evaluate_0.7
## [13] lattice_0.20-31      DBI_0.3.1
## [15] yaml_2.1.13          DESeq_1.20.0
## [17] genefilter_1.50.0    stringr_1.0.0
## [19] hwriter_1.3.2        knitr_1.10.5
## [21] aroma.light_2.4.0    grid_3.2.0
## [23] AnnotationDbi_1.30.1 survival_2.38-1
## [25] XML_3.98-1.2         rmarkdown_0.6.1
## [27] latticeExtra_0.6-26  geneplotter_1.46.0
## [29] lambda.r_1.1.7       magrittr_1.5
## [31] codetools_0.2-11     MASS_7.3-40
## [33] splines_3.2.0        htmltools_0.2.6
## [35] matrixStats_0.14.0   xtable_1.7-4
## [37] stringi_0.4-1        R.oo_1.19.0
```
